# Establishing a physiologically based pharmacokinetic framework for aldehyde oxidase and dual aldehyde oxidase‐CYP substrates

**DOI:** 10.1002/psp4.13255

**Published:** 2024-10-23

**Authors:** Nihan Izat, Jayaprakasam Bolleddula, Pasquale Carione, Leticia Huertas Valentin, Robert S. Jones, Priyanka Kulkarni, Darren Moss, Vincent C. Peterkin, Dan‐Dan Tian, Andrea Treyer, Karthik Venkatakrishnan, Michael A. Zientek, Jill Barber, J. Brian Houston, Aleksandra Galetin, Daniel Scotcher

**Affiliations:** ^1^ Centre for Applied Pharmacokinetic Research The University of Manchester Manchester UK; ^2^ EMD Serono Research & Development Institute, Inc. Billerica Massachusetts USA; ^3^ Genentech, Inc. South San Francisco California USA; ^4^ GSK R&D Tres Cantos, Madrid Spain; ^5^ Takeda Pharmaceuticals Limited Cambridge Massachusetts USA; ^6^ Janssen Pharmaceutical Companies of Johnson & Johnson Beerse Belgium; ^7^ AbbVie Inc. North Chicago Illinois USA; ^8^ Eli Lilly and Company Indianapolis Indiana USA; ^9^ Takeda Pharmaceuticals Limited San Diego California USA; ^10^ Present address: iTeos Therapeutics Watertown Massachusetts USA; ^11^ Present address: Treeline Biosciences San Diego California USA

## Abstract

Aldehyde oxidase (AO) contributes to the clearance of many approved and investigational small molecule drugs, which are often dual substrates of AO and drug‐metabolizing enzymes such as cytochrome P450s (CYPs). As such, the lack of established framework for quantitative translation of the clinical pharmacologic correlates of AO‐mediated clearance represents an unmet need. This study aimed to evaluate the utility of physiologically based pharmacokinetic (PBPK) modeling in the development of AO and dual AO‐CYP substrates. PBPK models were developed for capmatinib, idelalisib, lenvatinib, zaleplon, ziprasidone, and zoniporide, incorporating in vitro functional data from human liver subcellular fractions and human hepatocytes. Prediction of metabolic elimination with/without the additional empirical scaling factors (ESFs) was assessed. Clinical pharmacokinetics, human mass balance, and drug–drug interaction (DDI) studies with CYP3A4 modulators, where available, were used to refine/verify the models. Due to the lack of clinically significant AO‐DDIs with known AO inhibitors, the fraction metabolized by AO (fm_AO_) was verified indirectly. Clearance predictions were improved by using ESFs (GMFE ≤1.4‐fold versus up to fivefold with physiologically‐based scaling only). Observed fm_i_ from mass balance studies were crucial for model verification/refinement, as illustrated by capmatinib, where the fm_AO_ (40%) was otherwise underpredicted up to fourfold. Subsequently, independent DDI studies with ketoconazole, itraconazole, rifampicin, and carbamazepine verified the fm_CYP3A4_, with predicted ratios of the area under the concentration–time curve (AUCR) within 1.5‐fold of the observations. In conclusion, this study provides a novel PBPK‐based framework for predicting AO‐mediated pharmacokinetics and quantitative assessment of clinical DDI risks for dual AO‐CYP substrates within a totality‐of‐evidence approach.

**Study Highlights**

**WHAT IS THE CURRENT KNOWLEDGE ON THE TOPIC?**

Aldehyde oxidase (AO) substrates have challenges in drug development because of the low confidence in the prediction of in vivo clearance and fraction metabolized (fm) via this enzyme in the preclinical stage. Thus, clinical pharmacology plans contain uncertainty, especially in drug–drug interaction (DDI) risk assessment for dual AO‐cytochrome P450 (CYPs) substrates. Empirical scaling factors (ESF) were reported to improve clearance predictions via in vitro‐in vivo extrapolation (IVIVE).

**WHAT QUESTION DID THIS STUDY ADDRESS?**

Can physiologically‐based pharmacokinetic (PBPK) modeling be utilized as a drug development tool for AO and dual AO‐CYP3A4 substrates?

**WHAT DOES THIS STUDY ADD TO OUR KNOWLEDGE?**

PBPK models of AO‐CYP3A4 dual substrates using ESF‐supported IVIVE can be used in the early assessment of AO‐mediated pharmacokinetics and DDI risks with CYP3A4 modulators. A totality‐of‐evidence approach bridging human mass balance, clinical pharmacokinetic, and CYP3A4‐based clinical DDI data is necessary to improve the predictive performance of PBPK models and increase confidence in drug development.

**HOW MIGHT THIS CHANGE DRUG DISCOVERY, DEVELOPMENT, AND/OR THERAPEUTICS?**

The presented PBPK framework represents a pragmatic approach to mitigate current challenges in the quantitative translation of AO‐mediated metabolism. Depending on the available data, various modeling strategies are proposed for different stages of drug development to inform clinical pharmacology strategies for AO and dual AO‐CYP substrates.

## INTRODUCTION

Aldehyde oxidase (AO) is a cytosolic enzyme contributing to the oxidation of aldehydes and azaheterocycles (e.g., kinase inhibitors), the hydrolysis of amides, and the reduction in N‐ and S‐oxides.^1^ The interest of the pharmaceutical industry in AO has grown in the last decade following examples of failures in clinical development linked to low bioavailability due to rapid metabolism, which was not predicted from preclinical species/in vitro systems.^2^ In addition, AO metabolites formed in vivo that were not found during in vitro screening have led to failures linked with dose‐limiting toxicity^3^ and drug–drug interactions (DDIs).^4^ These challenges are reflected by the limited number of AO substrates in the market (e.g., idelalisib and lenvatinib), and most of these show overlapping specificity with other enzymes such as cytochrome P450s (CYPs).

Currently, there is a lack of harmonized best practices in the pharmaceutical industry for using suitable in vitro tools to predict AO metabolism and fraction metabolized by AO (fm_AO_). Therefore, devising a clinical pharmacology plan for AO substrates is fraught with uncertainty. In addition, no established physiologically based pharmacokinetic (PBPK) modeling frameworks are available for translational prediction of pharmacokinetics and DDI risk for AO and dual AO‐CYP substrates, where accurate prediction of the effect of CYP modulation requires confidence in the estimated AO contribution.^5^

PBPK models are widely applied for the evaluation of CYP^6^‐ and transporter‐mediated DDIs.^7^, ^8^ However, confidence remains low for in vitro–in vivo extrapolation (IVIVE) and PBPK modeling of substrates of non‐CYP enzymes.^9^ Because of the limited successes of IVIVE, the majority of published PBPK models for non‐CYP enzyme substrates relied on middle‐out approaches, and availability of clinical data for clearance refinement/estimation. These efforts have typically refined model parameters manually and on an individual drug basis without a consensus on the best practices,^9^ in contrast to experience gained with CYPs.^6^

Several methods have been suggested to enhance the accuracy of clearance predictions for AO substrates. These include the use of innovative preclinical models such as humanized mice,^10^ application of empirical factors derived via static^5^, ^11^ or PBPK^12^ models to adjust predicted clearance from human hepatocytes (HH),^5^, ^11^ human liver S9,^5^ or human liver cytosols (HLC) data,^5^, ^12^ and accounting for extra‐hepatic AO/additional clearance in PBPK models.^12^, ^13^

However, early predictions of fraction metabolized (fm_i_) still rely on scaled data from in vitro systems. This approach is associated with uncertainty because of limited reproducibility between studies and reported disconnect compared to observed fm_AO_ from human mass balance studies, especially for dual AO‐CYP substrates.^5^ Although the first clinically relevant AO‐mediated DDI has been recently reported,^14^ there is a general lack of established clinical index AO inhibitors for routine DDI studies, which limits the evaluation of the disconnect between in vitro and in vivo fm_AO_.

Toward devising the clinical pharmacology plan for AO and dual AO‐CYP substrates, this study aimed to evaluate the utility of PBPK modeling for these substrates within drug development using a set of case studies. Various modeling strategies were evaluated and the reliability of IVIVE‐linked approaches for the early prediction of AO‐mediated pharmacokinetics and CYP3A4‐mediated DDI risks for dual substrates was assessed. A PBPK modeling framework was presented based on the totality‐of‐evidence from mass balance and in vitro reaction phenotyping, together with clinical pharmacokinetic data and results of clinical DDI studies with CYP3A4 modulators.

## METHODS

### Compound selection

Capmatinib, idelalisib, lenvatinib, zaleplon, ziprasidone, and zoniporide were selected for PBPK model development from our published database^5^ considering the availability of in vivo human pharmacokinetic and mass balance studies, along with CYP3A4‐mediated DDI data for dual AO‐CYP3A4 substrates for model development, refinement, and/or verification purposes. The drugs cover a wide range of in vivo hepatic unbound intrinsic clearance (CL_int,u_; 37–5012 mL/min/kg) and fm_AO_ (0.17–0.74), and with exception of zoniporide, are dual AO‐CYP3A4 substrates.

#### Experimental

In vitro data were generated to inform the PBPK models of the selected drugs (Supplement 1 in Appendix S1; Table S1). Subcellular fractions of human and dog liver were sourced from XenoTech (Kansas City, KS, USA). In vitro CL_int,u_ was measured with substrate depletion and binding assays for all compounds (1 μM) in human liver microsomes (HLM; 0.25–0.5 mg/mL) and HLC (1 mg/mL). HLC pool was prepared from livers perfused with Custodiol HTK (histidine tryptophan ketoglutarate) solution, marketed as having preserved “high AO/XO activity.”^15^ Dog liver cytosols (DLC; 1 mg/mL) were used to investigate cytosolic binding in the absence of AO. Routine in vitro assays were used to quantify unbound fraction in plasma (fu_p_), blood‐to‐plasma ratio (B/P), and apparent permeability (P_app_) through transwell transport assays utilizing canine Mdr1 knockout and empty vector‐transfected MDCKI cells.

#### Development and refinement of PBPK models

**FIGURE 1:**
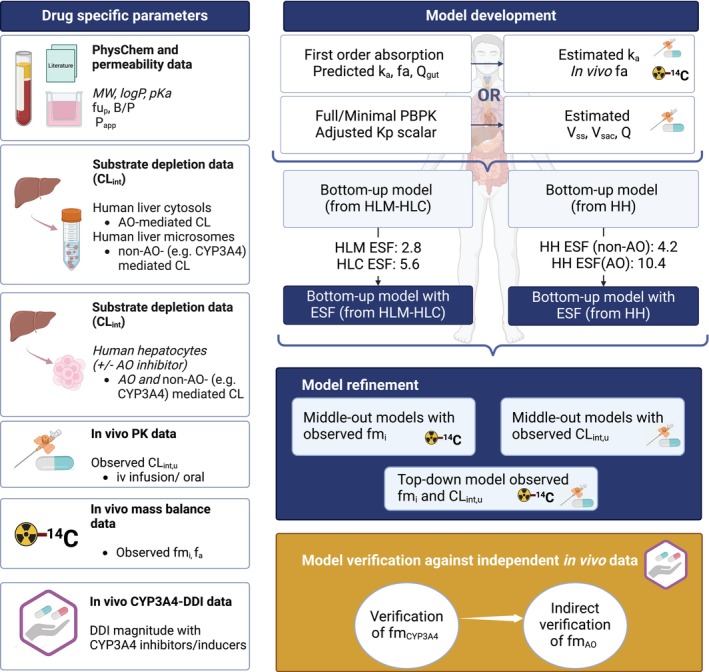
The PBPK model workflow. A stepwise approach was applied to predict or estimate absorption and distribution components parameters. In vitro human liver microsomes (HLM) and cytosols (HLC) data, or literature human hepatocytes (HH) data, were utilized in bottom‐up models. Absorption and distribution parameters were either predicted from the in vitro data or optimized by fitting the model to in vivo data. Empirical scaling factors (ESF) were applied to improve clearance and fraction metabolized (fm_i_) predictions. ESFs for in vitro‐in vivo extrapolation of aldehyde oxidase (AO)‐ and non‐AO‐mediated CL_int,u_ from human liver cytosols and microsomes, were 5.6^5^ and 2.8,^22^ respectively. ESFs for in vitro‐in vivo extrapolation of AO‐ and non‐AO‐mediated CL_int,u_ from human hepatocytes were 10.4^5^ and 4.2^22^, respectively. Bottom‐up models with ESFs were then refined by (i) the observed fm_i_ data from human mass balance studies (middle‐out models with observed fm_i_), or (ii) the CL_int,u_ back‐calculated from observed plasma clearance (middle‐out models with observed CL_int,u_). In the top‐down model, both observed fm_i_ and clearance data were used in model refinement. CYP3A4‐mediated drug–drug interaction studies were used in model verification. Drug‐specific parameters were either generated or derived from the literature (italic). B/P, blood‐to‐plasma ratio; CL_int,u_, unbound intrinsic clearance; CYP3A4, cytochrome P450 3A4; DDI, drug–drug interaction; fa, fraction absorbed; Kp scalar, scalar applied all predicted tissue partition coefficients; logP, the octanol–water partition coefficient; MW, molecular weight; *P*_app_, apparent permeability; PBPK, physiologically‐based pharmacokinetic model; pKa, the acid dissociation constant; *Q*, the inter‐compartment clearance in compartmental modeling; *Q*_gut_, a nominal flow in gut model; *V*_ss_, volume of distribution at steady state; *V*_sac_, volume of single adjusting compartment.

PBPK modeling and simulations were performed following a stepwise approach (Figure 1) using the Simcyp simulator (version 21; Certara, Sheffield, UK). The Simcyp Healthy Volunteers population file was used with the following modifications. AO was defined as a cytosolic enzyme active in the liver (37% CV in abundance based on human liver S9, *n* = 21) and kidney (52% CV in abundance based on human kidney S9, *n* = 22)^16^; relative abundance of AO in kidney to the liver was set to 10%.^16^ Contribution of other extrahepatic AO metabolic clearance was considered negligible based on unquantifiable AO abundance reported in the human intestine, heart, and lung S9.^16^ Number of hepatocytes, cytosolic, and microsomal protein (mg) per gram liver (CV%) were set to 120 × 10^6^ (42%),^17^ 80.7 (30%),^18^ and 40 (27%),^19^ respectively. Cytosolic and microsomal protein (mg) per gram kidney cortex were assumed to be 53.3 (30%) and 26.2 (30%), respectively.^20^

Drug‐specific in vitro data were generated (Section 3) or collated from peer‐reviewed literature, FDA Clinical Pharmacology Review Summaries, and United States Prescribing Information. Details of the modeling for absorption and distribution parameters are provided in Supplement 2 in Appendix S1.

Bottom‐up predictions of AO‐mediated pharmacokinetics using unbound intrinsic clearance (CL_int,u_) data from different in vitro systems were explored. Cytosolic and microsomal CL_int,u_ were assumed to correspond to AO‐ and CYP3A4‐mediated CL, respectively, for dual AO‐CYP3A4 substrates, if the contribution of other metabolizing enzymes was minimal. CL_int,u_ data from human hepatocytes assay in the presence and absence of AO inhibitor hydralazine (25 μM) were collected from Toselli et al.^21^ except for ziprasidone, for which in‐house data (without inhibitor^5^) were used and corrected for incubational binding (fu_hep_), as previously described.^5^ Similarly, CL_int,u_ measured in the presence of hydralazine was attributed to CYP3A4.

CL_int,u_ data were then implemented in PBPK models. Due to the general trend of underprediction of in vivo CL observed for AO drugs, previously reported empirical scaling factors (ESFs)^5^ were used in addition to physiological scalars. ESFs for HLC (5.6)^5^ and HLM (2.8)^22^ were applied to CL_int,u,AO_ and CL_int,u,other_ (e.g., CYP3A4), respectively. For hepatocyte data, ESF of 10.4,^5^ derived from mostly high fm_AO_ substrates, was applied to CL_int,u,AO_, whereas CL_int,u,other_ (obtained in hepatocytes with hydralazine) was scaled by ESF of 4.2,^22^ based on non‐AO drugs.^22^ The renal and biliary clearance were included in the models, when applicable.

Human mass balance and clinical pharmacokinetics data after i.v./oral administration (Supplement 3 in Appendix S1; Tables S2 and S3) were utilized to obtain observed fm_i_ and CL_int,u_, respectively.^23^, ^24^ When observed fm_i_ contained uncertainty (Table S2), minimum and maximum fm_i_ were tested. Virtual subjects (*n* = 1000) were generated using Simcyp, reflecting the subjects in respective clinical study (Table S3). The mean of the simulated physiological scalars, hepatic blood flow, and predicted/optimized fraction absorbed (fa) and fraction escaping gut metabolism (Fg) were then used in the back‐calculation of in vivo total CL_int,u_. The well‐stirred liver model was utilized if intravenous (i.v.) data were available or calculated from oral CL, as described previously.^5^ CL_int,u_ was then scaled back to cytosolic or microsomal CL_int,u_ using physiological scalars and fm_i_. Middle‐out models were developed using either observed fm_i_ or CL_int,u_ to refine ESF‐supported bottom‐up models. In the top‐down model, both parameters were fixed to observed values (Figure 1).

#### Prediction of CYP3A4 DDIs for dual AO‐CYP3A4 substrates and model verification

Previously verified PBPK models for CYP3A4 inhibitors (itraconazole and ketoconazole) and inducers (carbamazepine and rifampicin‐multiple dose) from the Simcyp v.21 compound library were used for DDI simulations (Supplement 4 in Appendix S1 for references). Simulations were performed with a minimum study size of 100 virtual subjects (10–25 trials) following the specific trial designs (Table S3). The predictive performance of PBPK model simulations was assessed by comparing the observed mean of plasma concentration data with the mean, 5th, and 95th percentiles of predictions. Overall model performances were evaluated using the geometric mean fold error (GMFE) and root mean square error (RMSE)^25^ for pharmacokinetic parameters. Predictions were considered successful if *C*_max_ and AUC_inf_ were predicted within twofold of observed values from an independent in vivo study (control condition). Predicted versus observed AUC ratios (AUCR) were compared to verify fm_i_; Guest et al.^26^ acceptance criteria were used considering the weak magnitude of the majority of CYP3A4 DDIs reported for selected drugs. Additionally, AO‐mediated DDI risks with known in vitro AO inhibitors including ethinyl estradiol, cimetidine, raloxifene, icotinib, and erlotinib were explored using the semi‐mechanistic model^27^ (Supplement 5 in Appendix S1; Table S4).

## RESULTS

### Development of bottom‐up PBPK models and their predictive performance

**FIGURE 2:**
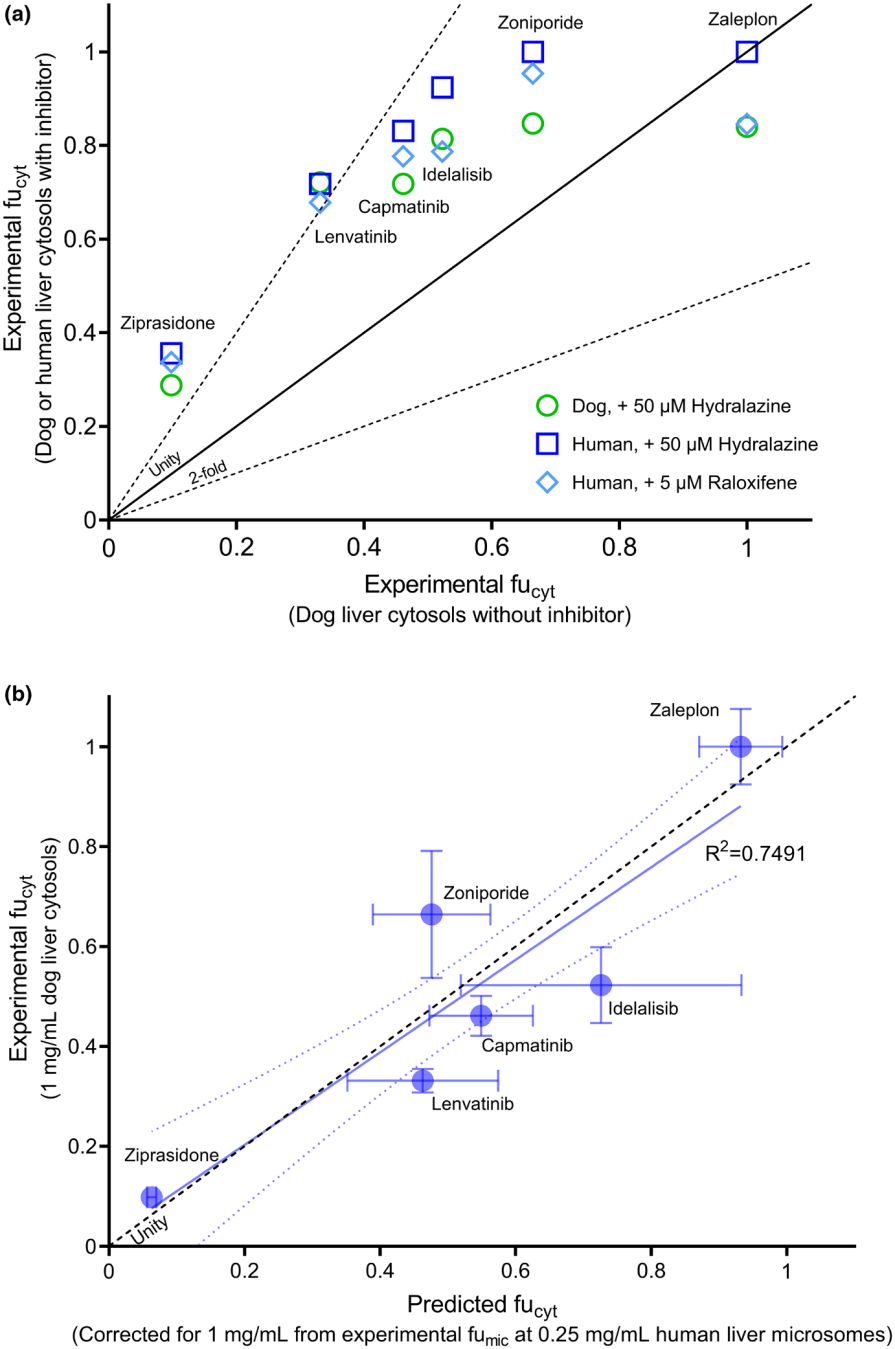
Unbound fractions in cytosolic incubations (fu_cyt_). (a) comparison of fu_cyt_ in dog liver cytosols in the absence of aldehyde oxidase inhibitor vs. in the presence of aldehyde oxidase inhibitors (hydralazine, raloxifene) dog or human liver cytosols. The solid and dashed lines represent the line of unity and twofold difference, respectively. (b) relationship between the predicted fu_cyt_ from experimental fu_mic_ (after correcting for the protein concentration) and fu_cyt_ measured in dog liver in the absence of AO inhibitors (mean ± SD). The solid and dashed blue lines represent the best‐fit line and its 95% confidence intervals, respectively. Line of unity is shown with black dashed‐line.

Plasma binding, B/P, *P*_app_, microsomal and cytosolic CL_int_, and incubational binding data were experimentally measured for drugs in the dataset (Tables S5–S9; Figure S1). Cytosolic CL_int_ ranged from 1 to 16 μL/min/mg for lenvatinib and zoniporide, respectively. fu_cyt_ was higher in the presence of AO inhibitors (50 μM hydralazine/5 μM raloxifene) in both dog and human cytosols, compared with the absence of AO inhibitor in dog (Figure 2a). The experimental fu_mic_ was strongly correlated with fu_cyt_ in dog cytosols (no AO inhibitor) (Figure 2b).

**FIGURE 3:**
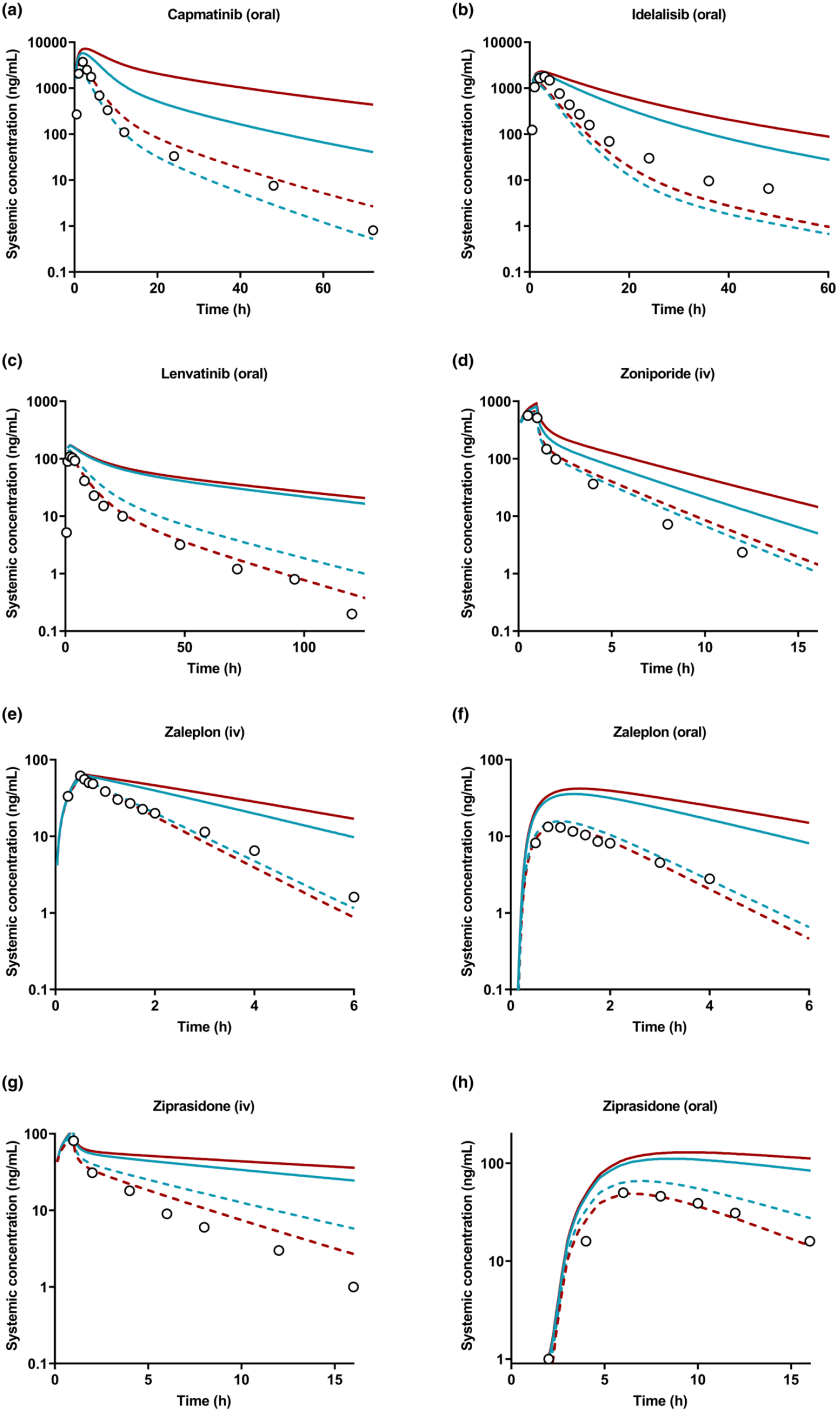
Simulation of the pharmacokinetics by bottom‐up models in the presence (dashed lines) and absence (solid lines) of empirical scaling factors using human liver microsomes and cytosols (blue) or human hepatocytes (red) data of (a) capmatinib [oral 600 mg], (b) idelalisib [oral; 150 mg], (c) lenvatinib [oral; 10 mg], (d) zoniporide [i.v. infusion; 80 mg], (e) zaleplon [i.v. infusion; 5 mg], (f) zaleplon [oral; 5 mg], (g) ziprasidone [i.v. infusion; 5 mg], (h) ziprasidone [oral; 20 mg]. Circle symbols represent observed data.

Table 1 summarizes all PBPK model input parameters. Initially, absorption and distribution components of PBPK models were confirmed by using fixed observed CL_iv/oral_; simulated plasma concentration–time profiles agreed with observed mean profiles and pharmacokinetic parameters were within twofold of observed (data not shown). Subsequently, various modeling approaches ranging from bottom‐up to middle‐out were investigated to predict elimination and compared to a top‐down approach (Figures S2–S10). Bottom‐up PBPK models using the HLM and HLC data, or HH data, underpredicted CL_iv/oral_ for all AO/AO‐CYP3A4 compounds (Figure 3; Tables S10 and S11). The overprediction of AUC_inf_ was evident for all compounds (GMFE = 3.5 and 5.7 by HLM‐HLC and HH data, respectively; Table S12). *C*_max_ were predicted within twofold of observed using either of the in vitro inputs (GMFE = 1.4–1.6). When database ESFs were applied in addition to physiological scalars, CL_iv/oral_ predictions were improved (GMFE and AFE ≤1.4‐fold; Table S12), and the simulated pharmacokinetic profiles of all compounds were in good agreement with the observed data after i.v./oral administration (Figure 3). However, bottom‐up predictions of fm_i_ varied depending on the in vitro system used, for example, predicted fm_AO_ of capmatinib was 0.1–0.2 using HH, and 0.4–0.5 using HLM‐HLC data (with‐without ESFs) (Figure S11).

**TABLE 1:** Input parameters used in physiologically based pharmacokinetic model development.

	Capmatinib	Idelalisib	Lenvatinib	Zaleplon	Ziprasidone	Zoniporide
MW (g/mol)	412.43	415.42	426.85	305.34	412.94	320.35
Compound type	Ampholyte	Diprotic base	Ampholyte	Neutral	Diprotic base	Diprotic base
logP	3.04^44^	2^45^	3.3^46^	1.3^12^	4.53^12^	1.15^12^
pKa_1_	12.8^44^	1.6^45^	12.37^44^	n/a	8.24^12^	3.4^47^
pKa_2_	4.55^44^	3.4^45^	5.05^46^	n/a	6.31^12^	7.2^47^
fu_p_	0.0516	0.0536	0.0247	0.569	0.0029	0.288
B/P	1.37, 2.56, 3.58, 3.95 (at 5000, 1000, 50,10 ng/mL)	0.7	0.645	0.91	0.55 (assumed from measured 0.455)	0.86
Plasma protein	HSA	AGP	HSA	HSA	AGP	AGP
Absorption model	First‐Order	First‐Order	First‐Order	First order	First order	n/a
P_app_ (10^−6^ cm/s)^a^	32	16.2	29.8	38.9	3.15	1.72
fa	0.67^48^	0.965	0.989	0.994	0.744	n/a
ka (1/h)	0.623^b^	1.20	1.77	2.10	0.417	n/a
t_lag_ (h)	n/a	n/a	n/a	0.25^49^ (30%)	4 (30%)	n/a
fu_gut_ (assumed)	fu_p_	fu_p_	fu_p_	fu_p_	fu_p_	n/a
Q_gut_ (L/h) (predicted)	14.4	11.9	14.1	15.0	6.21	n/a
Distribution model	Minimal PBPK	Minimal PBPK	Minimal PBPK	Minimal PBPK	Full PBPK	Full PBPK
Method	Parameter estimation	Parameter estimation	Parameter estimation	Method 2	Method 1	Method 2
Kp scalar	1	1	1	1.7 (optimized)	0.56 (optimized)	0.64 (optimized)
V_ss_ (L/kg)	0.620	0.811	1.33	1.16	1.01	1.70
V_sac_ (L/kg)	0.218	0.108	0.492	0.00001	n/a	n/a
Q (L/h)	1.81	0.347	1.53	0	n/a	n/a
CL_iv/oral_ ^c^ (L/h) [CV%]	42.3 [60] (weighted mean and CV)	15.3 [28.2] (oral)	9.9 [34.3] (oral)	52.51 [22.5] (i.v., female) 71.58 [20.2] (i.v., male)	23.04 [14] (i.v.)	95.35 [5.7] (i.v.)
CL_Renal_ ^c^ (L/h)	Negligible	0.459	0.040	Negligible	Negligible	16.2
HLM CL_int_ (μL/min/mg protein)	30.4	9.73	7.43	5.95	71.9	5.19
fu_mic_	0.826	0.828	0.627	0.964	0.210	0.642
HLC CL_int_ (μL/min/mg protein)	4.04	3.82	0.982	4.12	4.67	16.5
fu_cyt_ ^d^	0.461	0.523	0.331	1.000	0.098	0.664
HH CL_int. non‐AO_ (μL/min/10^6^ hepatocytes)^e^	7.8^21^	3.9^21^	0.5^21^	0.8^21^	21.8^5^,^f^	1.6^21^
HH CL_int,AO_ (μL/min/10^6^ hepatocytes)^e^	0.7^21^	1.6^21^	0.3^21^	1.9^21^		5.3^21^
fu_hep_ ^g^	0.671	0.836	0.137	0.830	0.261	0.890

Abbreviations: AGP, α(1)‐acid glycoprotein; B/P, blood‐to‐plasma ratio; CL_int_, intrinsic clearance; CL_iv/oral_, observed plasma clearance after intravenous (i.v.) or oral administration; CL_Renal_, observed renal clearance; fu_cyt_, incubational unbound fraction in cytosols; fu_hep_, incubational unbound fraction in human hepatocytes; fu_p_, unbound fraction in plasma; HH, human hepatocytes; HLC, human liver cytosols; HLM, human liver microsomes; HSA, human serum albumin; Kp scalar, scalar applied all predicted tissue partition coefficients; logP, the octanol–water partition coefficient; MW, molecular weight; *P*_app_, apparent permeability; pKa, the acid dissociation constant; *Q*, the inter‐compartment clearance in compartmental modeling; *Q*_gut_, a nominal flow in gut model; *V*_ss_, volume of distribution at steady state; *V*_sac_, volume of single adjusting compartment.

^a^ Effective permeability (*P*_eff_ × 10^−4^ cm/s) was predicted from *P*_app_ (×10^−6^ cm/s) via a user‐defined model [$\log\left(P_{\mathrm{eff}}\right)=\log\left(P_{\mathrm{app}}\right)\times 0.6431-0.34$].

^b^ Parameter estimated.

^c^ Obtained from in vivo pharmacokinetic studies, which are listed in Table S2.

^d^ Measured in dog liver cytosols in the absence of aldehyde oxidase inhibitor.

^e^ HH CL_int_,_u_ [with 25 μM hydralazine] was assumed as non‐AO CL_int,u_ (e.g., CYP3A4), the difference between total CL_int,u_ and non‐AO CL_int,u_ was assumed as CL_int,u,AO_. Values were converted to μL/min/mg protein via specific physiological scaling factors for each compound (from *n* = 1000 virtual individuals) prior to simulations.

^f^ No reaction phenotyping data with an aldehyde oxidase inhibitor were available.

^g^ Predicted from measured fu_hep_ ^11^ or fu_mic_ ^12^, ^21^ based on Hallifax and Houston algorithm^34^, ^50^ according to step‐wise approach.^5^

### Utility of human mass balance and clinical pharmacokinetic data

**FIGURE 4:**
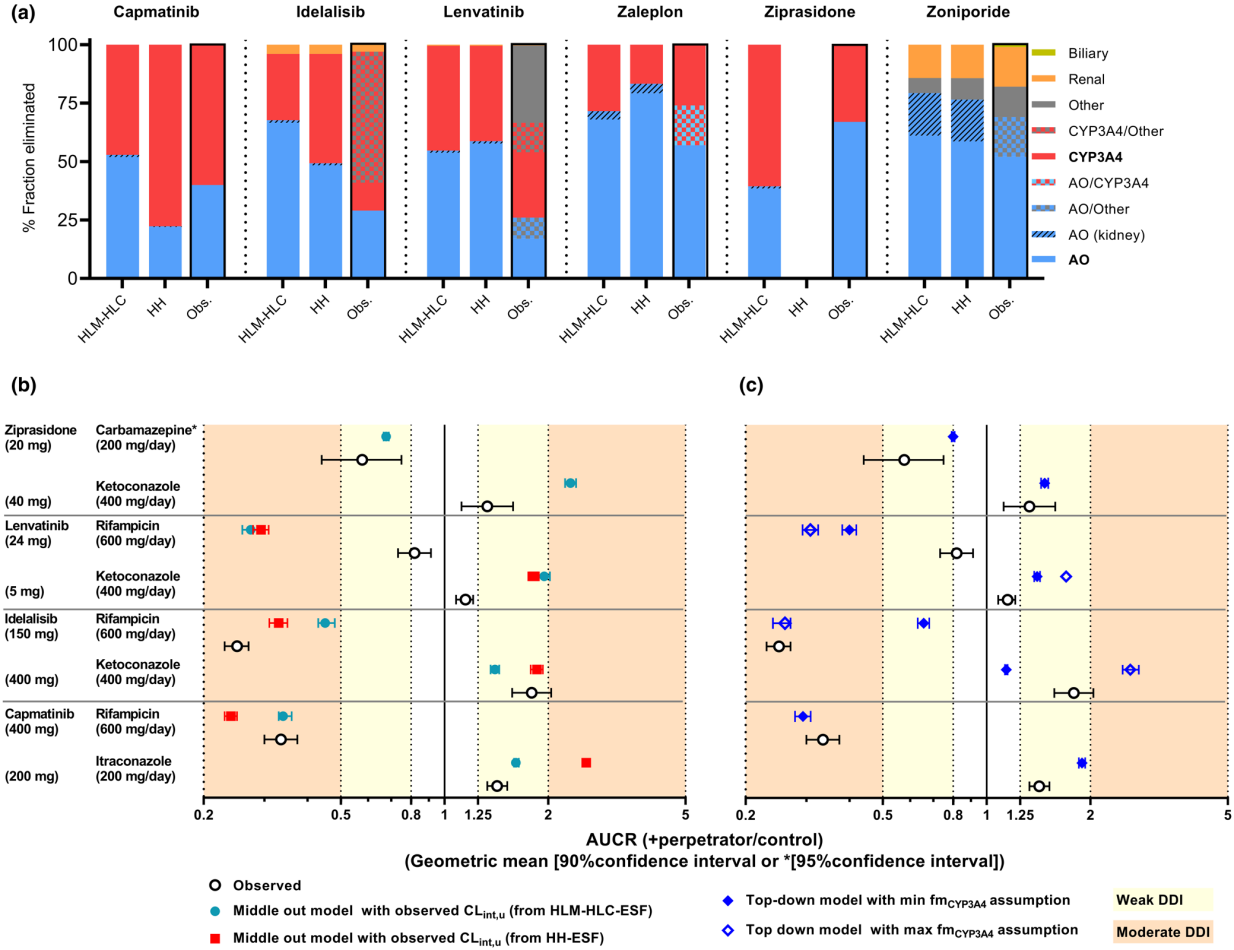
Physiologically‐based pharmacokinetic model bottom‐up predictions of fm (a) and drug–drug interaction studies with CYP3A4 inhibitors/inducers using middle‐out (b) and top‐down (c) models. (a) observed (human mass balance) % fraction eliminated compared with predictions using middle‐out PBPK models with predicted fm_i_ from either ESF‐supported human liver microsomes and cytosols (HLM‐HLC) data, or ESF‐supported human hepatocyte data (mean of predictions were plotted when multiple simulations were performed). (b) Observed and predicted geometric mean ratio of area under the time–concentration curve (AUCR) for clinical drug–drug interaction studies, simulated using middle‐out models with observed CL_int,u_ and predicted fm_i_. (c) Observed and predicted AUCR for clinical drug–drug interaction studies simulated using the top‐down model using observed fm_i_, incorporating minimum and maximum fm_CYP3A4_ assumptions, if available. CL_int,u_, unbound intrinsic clearance; DDI, drug–drug interaction; fm_CYP3A4_, fraction metabolized by cytochrome P40 3A4; HH, human hepatocytes; HLC, human liver cytosols; HLM, human liver microsomes.

Refinement of the fm_i_ values in the ESF‐supported bottom‐up models with the observed data from human mass balance studies resulted in the comparable predictive performance of AO‐mediated pharmacokinetics, as the total CL_int,u_ input remained unchanged. The GMFE for all predicted pharmacokinetic parameters via ESF‐supported bottom‐up models was 1.2–1.4, comparable to GMFE (1.2–1.3) with top‐down estimation of CL_int,u_. Predicted fm_i_ via ESF‐supported bottom‐up models were within twofold of the observed mean fm_i_ for majority of compounds. Exception was overprediction of fm_AO_ of idelalisib and lenvatinib by microsomal and cytosolic data (≤2.5‐fold) and underprediction of fm_AO_ of capmatinib by hepatocytes data (twofold). Up to 18% renal AO contribution was predicted for zoniporide due to its high observed CL_int,u_ (Figure 4a).

### PBPK model performances in predicting the risk of CYP3A4‐mediated drug–drug interactions

Pharmacokinetics observed in the control condition (without CYP3A4 inhibitors) served as independent data for PBPK model verification (Tables S13–S15). The predicted Fg was 0.98–1 for drugs with CYP3A4 DDI data, thus intestinal interaction was not considered relevant (Tables S10 and S11). Bottom‐up and middle‐out PBPK models using either microsomal‐cytosolic or hepatocytes CL_int,u_ data (with ESF) predicted pharmacokinetic parameters within twofold of observed in all cases. The top‐down model with observed CL_int,u_ performed well for all drugs (92% simulations within twofold error; *n* = 12) in control condition.

Predicted fm_CYP3A4_ values were within 1.5‐fold of the observed mean from mass balance studies except for ziprasidone via microsomal‐cytosolic, and zaleplon via hepatocyte data (Figure 4a). PBPK models using predicted fm_AO_ and fm_CYP_ from in vitro data successfully predicted 50%–62% of CYP3A4 DDIs within Guest et al. criteria, and in the correct DDI classification regardless of whether CL_int,u_ was based on IVIVE or refined by reverse translation (Table S15). In case of the top down model, 75% of simulations were within Guest et al. acceptance criteria^26^ except for lenvatinib‐ketoconazole (with maximum fm_CYP3A4_) and lenvatinib‐rifampicin interaction (Tables S13–S15).

The top‐down model with observed fm_i_ correctly predicted low‐moderate DDIs for capmatinib and ziprasidone. For idelalisib, observed DDI risk was weak‐to‐moderate with CYP3A4 inhibitor and inducer, respectively. Predictions based on minimum observed fm_CYP3A4_ of idelalisib underpredicted DDI risk. Therefore, it is crucial to consider the uncertainty in mass balance data as the models with maximum fm_CYP3A4_ predicted correctly moderate DDI between idelalisib and rifampicin. CYP3A4 DDIs for lenvatinib were overpredicted by all the models (Figure 4b,c). Predicted versus observed pharmacokinetic profiles with/without CYP3A4 perpetrators using individual models are shown in Figures S12–S18.

## DISCUSSION

A comprehensive assessment of DDIs and benefit/risk of investigational agents across populations and clinical contexts of use is a key component of drug development, regulatory review, and labelling. For AO substrates, there is a lack of established clinical pharmacology strategy and tools (e.g., strong inhibitors/inducers), coupled with limited success in PBPK modeling.^9^ Efficient and inclusive drug development of AO substrates is therefore hampered by a lack of confidence in state‐of‐the‐art model‐informed drug development approaches that are commonplace for substrates of CYP enzymes and clinically relevant transporters. In this study, PBPK models of six AO/dual AO‐CYP3A4 substrates were developed using bottom‐up approaches with either microsomal‐cytosolic or hepatocyte data. Models were then refined/verified by integrating clinical mass balance, pharmacokinetic, and CYP3A4 DDI data to assess the comparative performance of different quantitative translational approaches.

### Performance of IVIVE‐linked PBPK models for prediction of AO‐mediated clearance

Bottom‐up models demonstrated the underprediction of observed CL, which has been partly attributed to reduced AO enzyme activity following tissue processing.^28^ Although we generated the metabolic activity data in the high AO/XO activity labeled lot, the overall bias in CL predictions from this lot was comparable with the bias noted in our extensive literature database.^5^ Additionally, the AO abundance in this lot was comparable to different commercial HLC lots containing both University of Wisconsin‐ and HTK‐perfused donor livers (unpublished data). The possibility of AO activity in microsomes due to cytosolic contamination^29^ is assumed negligible. Therefore, system‐specific ESFs previously established^5^ were applied in PBPK models for both AO‐ and non‐AO mediated CL.

Measurement of cytosolic binding (fu_cyt_) is rarely reported.^30^ Considering that dogs do not express functionally active AO, dog liver cytosol was explored as an in vitro tool for the evaluation of nonspecific cytosolic binding.^31^ In parallel, we evaluated binding in human cytosol in the presence of a potent AO inhibitor (hydralazine/raloxifene^32^, ^33^) to minimize metabolic loss during the binding experiment. Nonspecific binding decreased in the presence of AO inhibitors in dog and human cytosols. This apparent inhibitor effect on fu_cyt_ was more pronounced for drugs with higher binding (e.g., ziprasidone, fu_cyt,dog_ = 0.098) as opposed to low‐binding drugs (e.g., zaleplon, fu_cyt,dog_ = 1). This effect may be due to competitive binding between the AO inhibitors and substrate in cytosol, although the main binding site is unlikely to be AO, as the protein is not expressed in dog. Given this possible experimental artifact, the measured fu_cyt_ values in dog were used for the purpose of PBPK modeling, with the assumption that nonspecific binding in human and dog liver cytosol was similar for these compounds. Additionally, fu_cyt_ measured in DLC were strongly correlated with predicted fu_mic_ in human (after correcting for total protein concentration), suggesting that extrapolation from fu_mic_ can be a surrogate approach to correct for nonspecific cytosolic binding (Figure 2b).^5^, ^34^

The application of ESFs previously determined using a large AO database of 22 drugs^5^ successfully improved predictions of pharmacokinetics for all drugs investigated (>91% within twofold). Database ESFs^5^, ^22^ provide an excellent initial refinement of predicted AO‐mediated clearance, which needs to be verified once clinical pharmacokinetic data are available. In our study, prediction of CL_iv/oral_ was comparable between the ESF‐supported bottom‐up models using IVIVE of either microsomal‐cytosolic or hepatocytes data (GMFE 1.2–1.4) and middle‐out/top‐down models using observed CL_int,u_ (GMFE 1.2).

AO abundance was reported to be unquantifiable in human intestine, heart, and lung S9,^16^ and extrahepatic AO‐activity was estimated to be <1% of the liver.^35^ Thus, PBPK models only considered extra‐hepatic AO expression in kidney (10% of the liver abundance)^16^ and this assumption may affect the accuracy of model‐predicted PK and DDIs. Predicted contribution of renal AO to CL was ≤3% across drugs investigated, except for zoniporide. Clearance of zoniporide exceeds the hepatic blood flow (observed CL_int,u,AO_ > 1000 mL/min/kg), and is predominantly AO‐mediated, with renal AO predicted to contribute ~20% to total metabolic clearance. Contribution of renal AO leading to over‐prediction of total fm_AO_ may occur for such high extraction compounds, although these are often de‐prioritized in drug development.

### Indirect evaluation of fm_AO_ accuracy by prediction of CYP3A4 DDI

Further verification of the PBPK models of dual AO and CYP3A4 substrates was done by predicting CYP3A4 induction by rifampicin and carbamazepine, and CYP3A4 inhibition by ketoconazole and itraconazole to confirm fm_CYP3A4_. Inhibition of AO by ketoconazole and itraconazole was expected to be negligible based on reported in vitro data of these CYP3A4 inhibitors on AO relative to CYP3A4 (Table S16). Evidence regarding PXR‐ or CAR‐mediated transcriptional upregulation of AO is lacking and therefore AO is not considered inducible by known CYP3A inducers such as rifampicin and carbamazepine.

Difficulties in accurately measuring fm_AO_ ^5^ contribute to the uncertainty in fm_CYP3A4_ and prediction of corresponding DDIs for dual AO‐CYP3A4 substrates. For early assessment of DDI risk via bottom‐up models, we adopted the conservative approach by assigning clearance via human liver cytosols and microsomes to AO and CYP3A4 metabolism, respectively, assuming other pathways were negligible. Application of specific ESFs to AO‐ and non‐AO‐mediated CL_int,u_ affected the fm_AO_ versus fm_CYP3A4_ predictions (Figure S11). For instance, predicted fm_AO_ of ziprasidone via microsomal and cytosolic data increased from 0.24 to 0.38 with ESFs, which reduced the overprediction of fm_CYP3A4_ and CYP3A4 DDI risk. In most cases, predicted fm_AO_ by the models using ESF‐supported microsomal and cytosolic data were above the observed fm_AO_ range from mass balance studies, except for ziprasidone (Figure 4a).

Overall, bottom‐up/middle‐out PBPK models using predicted fm_i_ from ESF‐supported microsomal‐cytosolic data were more reliable in predicting CYP3A4 DDIs (*n* = 8 simulations; 63% within acceptance criteria^26^) than models using hepatocyte data (*n* = 6 simulations; 50% within acceptance criteria^26^). Underestimation of in vivo fm_AO_ (i.e., overestimation of fm for non‐AO routes) may occur in reaction phenotyping assays in hepatocytes due to faster decline of AO activity compared with other enzymes following tissue processing.^36^ Incomplete AO inhibition in vitro by hydralazine and non‐selectivity toward CYPs with higher concentrations^37^, ^38^ may also contribute to mispredictions of fm_AO_ via reaction phenotyping, as illustrated in capmatinib case by 4‐fold lower fm_AO_ compared to observed. Despite applying specific ESFs for AO and non‐AO‐ mediated CL_int,u_, predicted fm_AO_ of capmatinib was still 2‐fold lower than the observed (0.4). Thus, fm_CYP3A4_ of capmatinib and CYP3A4 DDIs between capmatinib and itraconazole were overpredicted (Figure 4a,b). Notably, icotinib has recently been proposed as selective in vitro AO inhibitor, offering a possibility to estimate fm_AO_ in closer alignment with observed values compared to hydralazine.^38^

The effects of ketoconazole, rifampicin, and carbamazepine on capmatinib and ziprasidone were predicted well with models refined by fm_CYP3A4_ from mass balance studies. Prediction of idelalisib CYP3A4 DDI was confounded by high uncertainty in fm_CYP3A4_ (0.12–0.68) and pharmacokinetic nonlinearity of idelalisib at 400 mg (ketoconazole study), although PBPK model was verified for its clinical dose (150 mg). For lenvatinib, rifampicin and ketoconazole DDIs were overpredicted even with observed minimum fm_CYP3A4_ of 0.28, confirming that CYP3A4 only marginally contributes to elimination of lenvatinib. Mass balance data for lenvatinib came from cancer patients, and disease‐related changes may have affected the abundance/ activity of enzymes, and the fm_i_ estimates to some extent. In instances of uncertainty regarding the identification of AO‐mediated primary and secondary metabolites from mass‐balance data, verification of observed fm_i_ against clinical DDI data, which is currently limited to non‐AO pathways (e.g., CYP3A4), is important. However, this approach may be insufficient when the non‐AO pathway is poorly defined (e.g., zoniporide). Therefore, conducting PBPK simulations using minimum and maximum fm_i_ assumptions is recommended for robust DDI risk assessment (Figure 4c).

### Current perspectives on assessing AO‐mediated DDI risks

**FIGURE 5:**
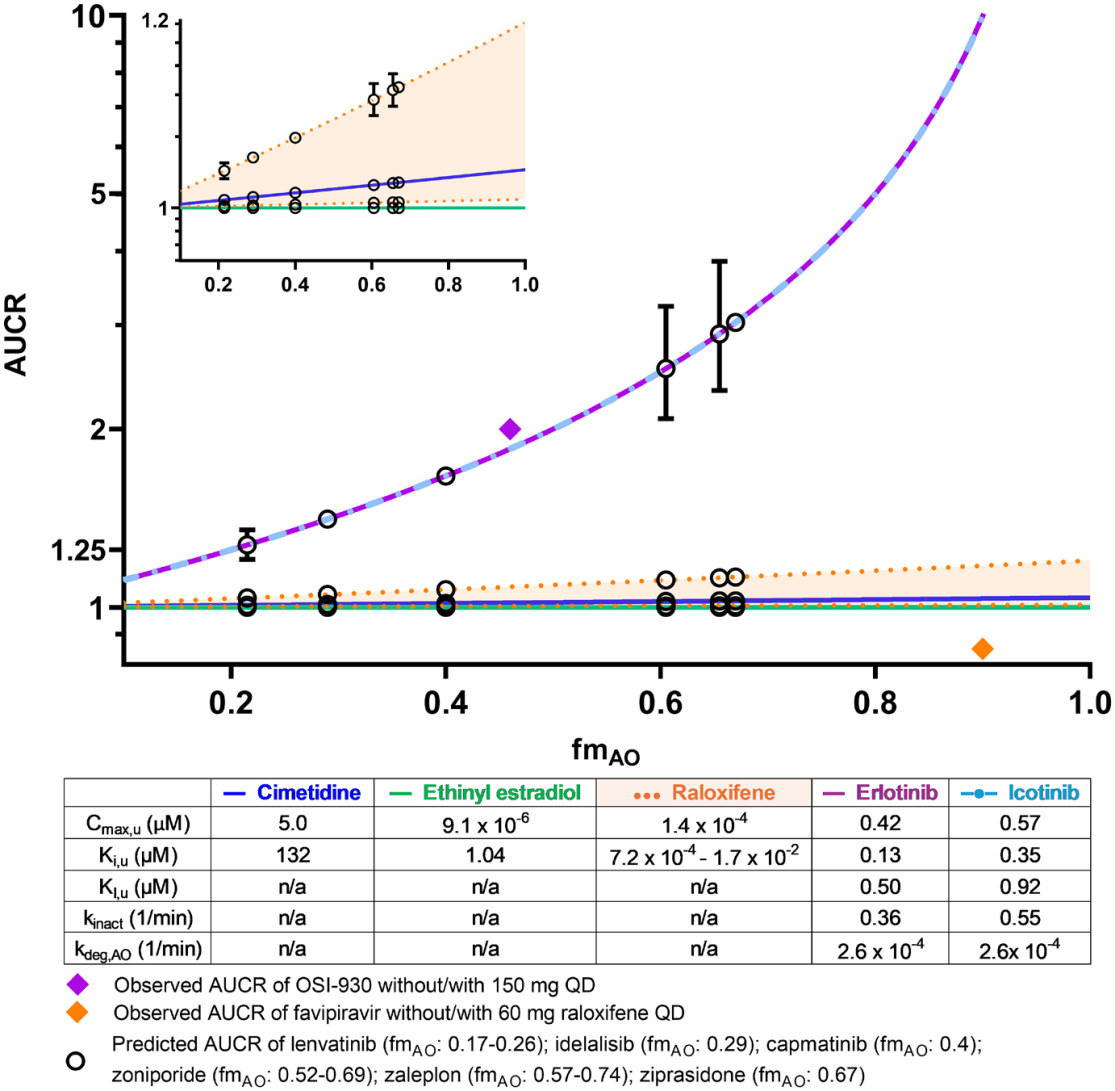
The predicted area under the time–concentration curve ratio (AUCR) via the semi‐mechanistic model to evaluate the interaction risk between aldehyde oxidase inhibitors (cimetidine, ethinyl estradiol, raloxifene, icotinib and erlotinib) and victim compounds with a range of fm_AO_ based on the parameters in Table S4 and limited observed AUCR reported. Error bars represent the minimum and maximum predicted AUCR around the mean when minimum and maximum fm_AO_ assumptions were applied due to the uncertainty in mass balance data. The shaded region for raloxifene represents the range of predicted AUCR when minimum and maximum reported *K*_i,u_ value was used. Due to the lack of *k*_deg,AO_ in the literature, the average of reported P450 *k*_deg_ values (0.00026 min^−1^) was used, as done previously.^38^ However, *k*_deg,AO_ value should be refined by monitoring the recovery of AO to improve the ability to predict AO time‐dependent inhibition in the future. Further information on studied AO inhibitors and available clinical evidence is provided in Table S17.

Recent report of the first clinical AO‐mediated DDI with erlotinib^14^ highlighted the need to explore the initial AO DDI risk for six substrates investigated. An exposure margin was predicted based on the ratio of *C*_max,u_ and K_i,u_ of the known AO inhibitors at the high therapeutic doses. Weak to moderate AO DDI risk was predicted for investigated AO substrates in the presence of time‐dependent AO inhibitors such as erlotinib and icotinib (Figure 5), which agreed with the observed DDI between erlotinib and OSI‐930 (fm_AO_ = 0.46; AUCR = 2^14^). High incidence of adverse effects by erlotinib and icotinib after multiple doses^39^, ^40^ may preclude their routine use in clinical DDI studies in healthy subjects (summary of AO DDIs is shown in Table S17). For the remaining in vitro inhibitors, even at the highest reported *C*_max,u_/K_i,u_, ethinyl estradiol, cimetidine, and raloxifene are not expected to cause clinically significant AO DDIs (Figure 5). Clinical DDI studies with cimetidine (AO and CYP3A4 inhibitor) also demonstrated no/weak DDIs for dual AO‐CYP3A4 substrates ziprasidone (fm_AO_ = 0.67; AUCR = 1.06^41^) and zaleplon (fm_AO_ = 0.57–0.74; AUCR = 1.85^42^), and raloxifene had minimal effect on favipiravir exposure (fm_AO_ = 0.9; AUCR = 0.85^43^). Therefore, conducting dedicated clinical DDI studies with these inhibitors is unlikely to be a useful component of the clinical pharmacology plan for most AO substrates.

**FIGURE 6:**
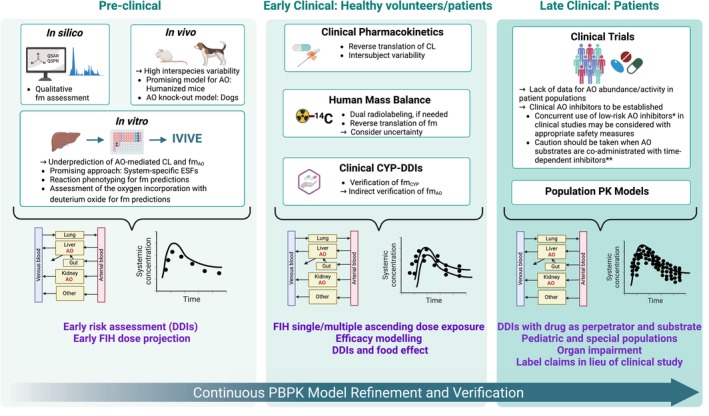
Challenges and opportunities in PBPK modeling framework for aldehyde oxidase (AO) and dual AO and cytochrome P450 (CYPs) substrates within drug development. CL, clearance; DDI, drug–drug interaction; FIH, first in human; fm, fraction metabolized *e.g. ethinyl estradiol, **e.g. erlotinib.

Until confirmatory clinical evidence for additional high fm_AO_ probes becomes available, we propose that concurrent use of these low‐risk competitive inhibitors (e.g., ethinyl estradiol) in clinical development may be considered with appropriate safety measures (Figure 6). This view aligns with ICH M12^27^ recommendations to avoid excluding patients who require such concomitant medications in clinical studies and gain information about the interaction potential early in drug development. However, caution should be taken when AO substrates are co‐administrated with high‐risk time‐dependent inhibitors (e.g., erlotinib).

### Implications for clinical pharmacology strategy

The results of PBPK modeling should be taken together with clinical considerations such as the safety profile of the investigational agent that is a potential DDI victim, its pharmacokinetic variability, exposure–safety relationships, and overall understanding of therapeutic index to inform decisions regarding DDI risk management and clinical pharmacology development planning. With collection of concomitant medication usage data (including start and stop dates) in Phase II/III clinical trials that incorporate informative sparse sampling for the investigational agent (victim), a population pharmacokinetic approach that considers the co‐administered AO inhibitor as a time‐varying covariate can provide complementary verification of expected DDI risk to support expectations from the PBPK model (Figure 6).

In conclusion, this study assessed the reliability of different PBPK modeling approaches for AO and dual AO‐CYP substrates and provided a modeling framework to accommodate varying availability of data at different stages of drug development. The applicability of PBPK as a tool for AO‐mediated DDI risk assessment relies on the availability of external fm_AO_ estimates from human mass balance studies, as selective in vivo AO inhibitors are currently limited to verify the in vitro fm_AO_ predictions. In case of dual AO‐CYP substrates, an alternative approach is to use CYP DDI data for the indirect verification of fm_AO_. Sensitivity analysis considering the uncertainty in fm_i_ estimates is important for prospective predictions of DDIs. Totality‐of‐evidence from in vitro reaction phenotyping, human mass balance and pharmacokinetic data, together with the clinical CYP‐based DDI data should support the development of robust PBPK models for AO and dual AO‐CYP substrates.

## AUTHOR CONTRIBUTIONS

N.I., J. Bolleddula, R.S.J., D.‐D.T., K.V., A.G., and D.S. wrote the manuscript; N.I., J. Bolleddula, R.S.J, D.M., V.C.P., A.T., D.‐D.T., K.V., M.A.Z., J.B.H., A.G., and D.S. designed the research; N.I., P.C., L.H.V., A.G., and D.S. performed the research; N.I., J. Bolleddula, P.C., L.H.V., R.S.J., P.K., D.M., V.C.P., A.T., D.‐D.T., K.V., M.A.Z., J. Barber, J.B.H., A.G., and D.S. analyzed the data.

## FUNDING INFORMATION

This work was supported by a consortium of pharmaceutical companies (AbbVie, Amgen, Eli Lilly, EMD Serono, Genentech, GSK, Johnson & Johnson, Roche, Servier, Takeda) within the Centre for Applied Pharmacokinetic Research at The University of Manchester.

## CONFLICT OF INTEREST STATEMENT

The authors declared no competing interests for this work.

## Supporting information


Appendix S1
