# Supplementary material for: Establishing a physiologically based pharmacokinetic framework for aldehyde oxidase and dual aldehyde oxidase‐CYP substrates
Source: CPT Pharmacometrics Syst Pharmacol. 2024 Oct 23;14(1):164–78. doi: 10.1002/psp4.13255 (PMC11706420; doi:10.1002/psp4.13255)
Supplement: Supplementary file 1 — Appendix S1 [file PSP4-14-164-s001.pdf]

SUPPLEMENTARY MATERIAL

Establishing a physiologically-based pharmacokinetic framework for aldehyde oxidase and dual aldehyde oxidase-CYP substrates

Nihan Izat [1]; Jayaprakasam Bolleddula [2, a]; Pasquale Carione [3]; Leticia Huertas Valentin [4]; Robert S. Jones [3]; Priyanka Kulkarni [5]; Darren Moss [6]; Vincent C. Peterkin [7]; Dan-Dan Tian [8]; Andrea Treyer [6]; Karthik Venkatakrishnan [2]; Michael A. Zientek [9, b]; Jill Barber [1]; J. Brian Houston [1]; Aleksandra Galetin [1]; Daniel Scotcher [1]

[1] Centre for Applied Pharmacokinetic Research, The University of Manchester, Manchester, UK
[2] EMD Serono Research & Development Institute, Inc., Billerica, Massachusetts
[3] Genentech, Inc., South San Francisco, California
[4] GSK R&D, Tres Cantos, Madrid, Spain
[5] Takeda Pharmaceuticals Limited, Cambridge, Massachusetts
[6] Janssen Pharmaceutical Companies of Johnson & Johnson, Beerse, Belgium
[7] AbbVie Inc., North Chicago, Illinois
[8] Eli Lilly and Company, Indianapolis, Indiana
[9] Takeda Pharmaceuticals Limited, San Diego, California

[a] Current address of J. Bolleddula is iTeos Therapeutics, Watertown, MA 02472 USA
[b] Current address of M.A.Z. is Treeline Biosciences, San Diego, CA 92121, USA

CONTENT **PAGES**

CONTENT	PAGES
Supplement 1. Materials and methods for conducted assays	3-12
Supplement 2. Details of PBPK model development (absorption and distribution)	13
Supplement 3. *In vivo* pharmacokinetic and human mass balance data	14-16
Supplement 4. References for Simcyp v21 default compound files	17-19
Supplement 5. Exploratory analysis to evaluate potential AO-mediated DDI risks	20-21
Supplement 6. Experimental results	22-27
Supplement 7. Observed vs. predicted pharmacokinetics from individual simulations	28-49
Supplement 8. Model verification and CYP3A4-mediated drug-drug interaction predictions	50-64
Supplement 9. Inhibitory effects of AO and CYP3A4 inhibitors	65-67
Supplementary Material References	68-71

Supplement 1. Materials and methods for conducted assays

Content:

Materials
Determination of plasma protein binding (fu_p)
Determination of blood-to-plasma ratio (B/P)
In vitro permeability (P_{app})
Cytosolic clearance incubations (CL_{int})
Microsomal clearance incubations (CL_{int})
Cytosolic binding (fu_{cyt})
Microsomal binding (fu_{mic})
Supplementary Table 1. Liquid Chromatography with tandem mass spectrometry (LC-MS-MS) conditions for each experiment conducted in various sites for compounds sourced from the same vendor.

Materials:

Capmatinib and idelalisib were purchased from TargetMol Chemicals Inc. (Boston, MA, USA), lenvatinib, zaleplon, ziprasidone and zoniporide were obtained from Sigma-Aldrich (St. Louis, MO, USA). Subcellular fractions of the human liver were sourced from XenoTech (Kansas City, KS, USA). Human liver microsomes (HLM) (n=200, mixed gender, and race) from the lot 1710084 and 1910096 (differing in 2 donors) were used for activity and microsomal binding assays, respectively. Human liver cytosols (HLC) (n=20, mixed gender, and race) from the lot 2210072 was used for cytosolic assays. Cytosolic binding was also tested using dog liver cytosols (DLC) from the lot 0610276 (n=4, male). The human biological samples were sourced ethically, and their research use was in accord with the terms of the informed consents under an IRB/EC approved protocol.

Determination of plasma protein binding (fu_p):

Human plasma (male, K2EDTA) was obtained from BioIVT (Baltimore, MD, USA). A pool of n=20 donors was prepared in-house (batch: 16062022). The plasma protein binding of compounds in human was determined by equilibrium dialysis using the rapid equilibrium dialysis (RED) device (Thermo Fisher, Waltham MA, USA) with a molecular weight cut-off of 6-8 kDa. Frozen blank human plasma was thawed and then spiked with the test or reference (warfarin and propranolol) compound stock solution to a final concentration of 1 µM (0.5 % DMSO). 300 µL of the fortified plasma samples were subjected to dialysis against 500 µL PBS buffer at pH 7.4, for 6 h at 37 °C in triplicate. After dialysis, 200 µl aliquots of the buffer and the plasma compartments were transferred into micronic tubes. To minimize the potential for non-specific binding to the micronic tubes the buffer samples were collected on to 50µl of 10 % bovine serum albumin (BSA). For the assessment of test compound stability in plasma, 300 µl aliquots of the spiked plasma solutions were transferred into micronic tubes and incubated in an air heated incubator at 37°C without shaking for 4 and 20 hours. The remaining spiked protein solutions were frozen and used to measure the initial start concentration (C_0) to calculate the recovery. Sample analysis was achieved by LC-MS/MS and absolute quantification of the drug levels in the buffer and plasma compartments was achieved via a matrix matched calibration curve (in the presence of an internal standard tolbutamide). All buffer and plasma sample sets were matrix matched

for analysis. The samples were centrifuged (15 minutes, 3400 g) and supernatants were transferred to a fresh plate for analysis. The unbound concentration (C_u), and the total concentration (C_{ED}) were determined by measurement of the concentration of the test compound in the buffer and plasma compartments of the dialysis cells, respectively. The fraction unbound was calculated using Eq. S1.

$fu_{plasma} = C_u/C_{ED}$ (Eq. S1)

The percentage recovery was calculated as the ratio of the sum of the amount in the buffer (A_u) and plasma (A_{ED}) compartments to the amount at time zero (A_0) (Eq. S2). The percentage stability was calculated as the ratio of the concentration of the test compound at the end of the incubation (C_t) time and C_0 (Eq. S3).

$\%\, recovery = ((A_u + A_{ED}) / A_0) \times 100$ (Eq. S2)

$\%\, stability = C_t/C_0$ (Eq. S3)

Determination of blood to plasma ratio (B/P):

Whole human blood was collected on the day of the experiment from healthy volunteers. The collection protocol was approved by an independent ethical committee and all donors signed the consent forms. All samples were registered in the Janssen Biobank.

Prewarmed blood aliquots were mixed with the compounds (final concentrations shown in Suppl. Table 5) with a final organic DMSO solvent concentration of ≤0.1% (v/v). After gentle mixing to avoid haemolysis, blood aliquots were immediately collected for protein precipitation (1:1:8 ratios of blood sample:DMSO:ACN) and centrifugation (10 minutes, 3800 g) and the supernatant was used as a 0-minute reference blood sample. At the same time, blood aliquots were taken and centrifuged (10 minutes, 1200 g) to obtain the plasma portion. Plasma aliquots were then immediately collected for protein precipitation (1:1:8 ratios of plasma sample:DMSO:ACN) and centrifugation (10 minutes, 3800 g) and the supernatant was used as a 0-minute reference plasma sample. The remainder of the blood fraction was incubated (30 min at 37 °C). Following incubation, 30-minute blood and plasma samples were taken and prepared as described for the reference 0-minute samples.

Mean compound concentration values (n=3 replicates) were calculated for both the blood and plasma samples. A blood-to-plasma concentration ratio was then determined using Eq. S4.

$R_b = C_b/C_p$ (Eq. S4)

where R_b is the blood-to-plasma ratio, C_b is the concentration of compound in blood at 30 min (ng/mL) and C_p is the concentration of compound in plasma (ng/mL).

***In vitro* permeability (P_{app}):**

MDCKI cells with canine Mdr1 knockout and empty vector transfection (cMdr1-KO MDCK-EV) were created at VectorBuilder (IL, USA) and stored in liquid nitrogen until used. Cells were cultured using Dulbecco's Modified Eagle Medium (DMEM, high glucose, GlutaMAX™, with pyruvate and phenol red) supplemented with penicillin-streptomycin mixture (final concentrations 100 U/mL penicillin and 100 µg/mL streptomycin). At day 0, the cells were seeded on 96-well cell culture inserts (pore size 0.4 µm, insert area 0.11 cm^2, Merck Millipore Cat# PSHT004R1) at 37400 cells/well. The media were replaced one day after seeding and one day before the experiment on day 4. Concentrations of the test compounds in the dosing solutions were determined prior to the assay. The cell monolayers were washed two times with experimental buffer (Hank's Balanced Salt Solution supplemented with 20 mM glucose and 25 mM HEPES) and both apical and basolateral compartments were pre-incubated in the experimental buffer for at least 10 minutes prior to initiating the incubation.

The experiment was started by applying the experimental buffer containing either 5 µM of test or reference compound (1 µM of ^{3}H atenolol, ^{3}H-propranolol, ^{3}H-digoxin, and 10 µM ^{14}C-mannitol) to the donor compartments (either the apical or basolateral compartments depending on the direction measured). If not already present in the condition, ^{14}C-mannitol was also included in all incubations to confirm the cellular integrity of the monolayers. Plates were incubated at 37 °C in a humidified incubator containing 5% CO_2. Conditions were performed in triplicate within the same experiment. At the end of the incubation period (120 min), aliquots from the acceptor and donor compartments were analysed by LC-MS/MS for absolute quantification of compounds and to check recovery. For reference compounds that contained radioactivity, liquid scintillation analysis was performed using a Perkin Elmer Tricarb 4910TR liquid scintillation counter (Waltham, MA, USA) using counting windows of 0-156 keV (^{14}C) or 0-18.6 (^{3}H). Transport across the monolayers expressed as the apparent permeability coefficients (P_{app}, cm/s) were calculated from the amount transported in the acceptor compartment

divided by the surface area (0.11 cm^2) of the insert, the initial measured concentration in the donor solution (C_0) vial and time (sec), according to Eq. S5

$$P_{app} = \frac{Amount\ transported}{Surface\ area \times C_0 \times Time} \text{ (Eq. S5)}$$

The efflux ratios of the compounds were calculated according to Eq. S6.

$$Efflux\ ratio = {P_{app,BA}}/{P_{app,AP}} \text{ (Eq. S6)}$$

where $P_{app,BA}$ is the apparent permeability (cm/s) from the basolateral to apical compartments and $P_{app,AB}$ is the apparent permeability (cm/s) from the apical to basolateral compartment.

Recovery values (given in %) in experimental plates after incubation were determined using Eq. S7

$$\%\ Rec_{end} = \frac{A_{end,apical} + A_{end,basolateral}}{A_0} \times 100 \text{ (Eq. S7)}$$

where % Rec_{end} is the percent recovery in the experimental plate at the end of the incubation, $A_{end,apical}$ is the amount of compound measured in the apical compartment at the end of the incubation, $A_{end,basolateral}$ is the amount of compound measured in the apical compartment at the end of the incubation and A_0 is the amount of compound measured in the dosing solution.

P_{app}-P_{eff} correlation using the power regression model[1] for the cMdr1-KO MDCK-EV assay (at pH 7.4/7.4 data for basic and neutral and 6.5/7.4 for acidic compounds) was established using in-house in vitro P_{app} (x 10^{-6} cm/s) data (unpublished) and literature P_{eff} (x 10^{-4} cm/s) data (n=15 compounds; Eq.S8)

$$\log(P_{eff}) = \log(P_{app}) \times 0.6431 - 0.34\ (Eq. S8)$$

Cytosolic clearance incubations (CL_{int}):

Cytosolic incubations were conducted in triplicate, with an additional assay with one replicate for each compound (except zaleplon) generated at a separate site either using an automated (Tecan Freedom Evo 200, MCA, Switzerland) or manual settings. Human liver cytosols (HLC) were stored at −80°C and rapidly thawed just before use at 37°C. HLC (final concentration, 1 mg/mL) was added to Eppendorf tubes in 96-well plate format and prewarmed at 37°C for 5 min. 45 µL of 50 mM potassium phosphate buffer with/without hydralazine (final concentration of 50 uM) was added to HLC. The final

concentration of organic solvent in the incubation media was ≤0.1%. Reactions were initiated by the addition of compound stock solutions (final concentration, 1 µM in 500 µL). At each time point (0, 5, 15, 30, 60 and 120 min), samples from the incubation mixture were added to quench solutions (ACN or ACN:MeOH containing pruvanserin or midazolam as internal standard, 80:20, v:v). The mixture was centrifuged at 3500-4000 rpm for 10-30 min (Beckman Coulter Allegra 25R Centrifuge; Brea, CA, USA). Each well was assayed for parent compound and internal standard, using a suitable LC/MS/MS method. Positive control carbazeran (1 µM) was run at the same time to ensure system integrity (data not shown). Incubations were repeated in the presence of an AO inhibitor (50 µM hydralazine) at one site (n=3).

Microsomal clearance incubations (CL_{int}):

Human liver microsomes were stored at −80°C and rapidly thawed just before use at 37°C. HLM concentrations in the incubations were 0.25 mg/mL (capmatinib and ziprasidone) or 0.5 mg/mL (idelalisib, lenvatinib, zaleplon and zoniporide). All compounds were mixed with HLM and 50 mM phosphate buffer. 450 µL of the mixture was added to 1.2mL cluster tubes in a 96-well block in duplicate and warmed in a water bath (Thermo Scientific Precision™,Waltham MA, USA) at 37°C for 5 min. Reactions were initiated by the addition of 50 µL from 10 mM of NADPH stock in 50 mM phosphate buffer (final NADPH concentration, 1 mM). The final incubation contained 1 µM test compound in 500 µL total volume with 0.02% DMSO. At each time point (5, 10, 20, 30, and 60 min), 60 µL of incubation mixture was added to 120 uL of quench solutions (MeOH:ACN, 1:1, v:v) containing internal standard (0.5 µM carbutamide). The mixture was centrifuged at 3000 rpm for 20 min (Eppendorf 5810R, Hamburg, Germany). Supernatant was injected to HPLC-MS/MS with for data analysis. Positive control verapamil (0.5 µM) was run down LC-MS/MS at the same time to ensure system integrity.

Data analysis for substrate depletion assays:

Data from the microsomal and cytosolic incubations were analyzed using GraFit 7 (Erithacus Software, Horley, UK) to determine the elimination rate constant (k), which was used in CL_{int} calculation (Eq. S9). A nonlinear single exponential fit was used for all compounds. A minimum of 15% substrate

depletion at the end of the incubation period was considered for data analysis except for lenvatinib in cytosolic and zoniporide in microsomal incubations, where only >10% depletion was observed. In cytosolic incubations, if 15% of depletion was achieved, 60 min was set as the endpoint except for lenvatinib.

$$CL_{int} = \frac{k \times volume\ of\ incubation}{amount\ of\ protein\ in\ assay} \text{ (Eq S9)}$$

Cytosolic binding (fu_{cyt}):

The fraction unbound in human liver cytosols (fu_{cyt}) of compounds was determined by equilibrium dialysis using the RED device (Thermo Fisher, Waltham MA, USA) with a molecular weight cut-off of 8 kDa. Dog or human liver cytosols at 1 mg/mL with 1 µM of compounds (300 µL, n=3 per compound) were added to the donor compartment in 100 mM potassium phosphate buffer with final DMSO concentration of 0.1 %. 50 µM hydralazine or 5 µM raloxifene was added in buffers containing human liver cytosols to inactivate AO, whereas dog was used as a natural AO knock-out model. 500 µL of 100 mM potassium phosphate buffer (pH 7.35) was added to the receiver chamber for each replicate. The RED device was then sealed with an adhesive foil and incubated on an orbital shaker at 450 rpm (VWR Symphony) at 37°C with 5 % CO2. After reaching equilibrium for 4 hours, aliquots from both the receiver (30µL) and donor (30µL) sides of the membrane were matrix matched, and then quenched with 180 µL of cold ACN with 100 nM propranolol (internal standard). The samples were placed in a Beckman Coulter Allegra x 12R Centrifuge (Brea, CA) for 15 minutes at 3700 rpm. After centrifugation, the supernatant of each sample was diluted 1:2 and analyzed by LC-MS/ MS. Matrix stability and recovery were assessed in triplicates and followed identical sample preparation.

Microsomal binding (fu_{mic}):

The fraction unbound in human liver microsomes (fu_{mic}) of compounds was determined by equilibrium dialysis using the high-throughput dialysis method [2] using membranes from HTDialysis, LLC (Gales Ferry, CT) having 12 to 14 kDa molecular mass cutoff. Deactivated HLM (by leaving the HLM incubation media on the bench overnight at ambient temperature) at 0.25 mg/mL with 2 µM of compounds (a final concentration of DMSO as 1%) was added to the donor compartment in 50 mM phosphate buffer containing 1 M $MgCl_2$. (150 µL, n=4 per compound). 150 µL of Dulbecco's PBS

buffer (pH 7.4) was added to the receiver chamber for each replicate. After reaching equilibrium for 6 h on a plate shaker (200 rpm) at 37°C in a humidified CO_2 incubator (5% CO_2), aliquots from both the receiver (45 µL) and donor (15 µL) sides of the membrane were matrix matched and analyzed by LC-MS/MS. Matrix stability (n=3) and % of recovery (n=4) were assessed.

Data analysis for binding in microsomal and cytosolic incubations:

Fraction unbound in microsomal (fu_{mic}) and cytosolic (fu_{cyt}) incubations were calculated via Eq. S10-11 based on the ratio of the peak areas of analyte and internal standard.

$$Peak\ Area\ Ratio = (Peak\ Area\ of\ Analyte)/(Peak\ Area\ of\ Internal\ S\tan d\ ard) \quad (\text{Eq.} S10)$$

$$fumic\ or\ fucyt = (Peak\ Area\ Ratio\ in\ receiver\ compartment)/$$
$$(Peak\ Area\ Ratio\ in\ donor\ compartment \quad (\text{Eq. S11})$$

The fu_{mic} values for idelalisib, lenvatinib, zaleplon and zoniporide were corrected for the protein concentration (0.5 mg/mL) using Hallifax and Houston algorithm (Hallifax and Houston, 2006). Matrix stability and recovery were assessed using Eq. S12 and Eq. S13.

$$\%stability = (Average\ peak\ area\ in\ matrix\ at\ the\ end\ of\ incubation)/$$
$$(Average\ peak\ area\ at\ T = 0) \times 100 \quad (\text{Eq. S12})$$

$$\%recovery = (A + B)/C \times 100 \quad (\text{Eq. S13})$$

where A is the average peak area ratio of all matrix samples, B is the average peak area ratio of all buffer samples multiplied with the buffer dilution factor and C is the average peak area ratio of T_0 samples.

Table S1. Liquid Chromatography with tandem mass spectrometry (LC-MS-MS) conditions for each experiment conducted in various sites for compounds sourced from the same vendor.

Assay(s)	LC-MS/MS system	HPLC condition	Data analysis	Compound	Positive electrospray ionisation: Transition	Cone voltage (declustering potential) (V)	Collision voltage/energy (eV)	Retention time (min)
**Plasma binding**<br><br>**B/P**<br><br>**Permeability**	Sciex Triple Quad 5500 MS (Framingham, MA, USA) equipped with a Shimadzu Nexera2 autosampler (Kyoto, Japan).	C18 UPLC column, Water (0. 1% Formic acid):ACN in gradient elution	Absolute quantification	Capmatinib<br>Idelalisib<br>Lenvatinib<br>Zaleplon<br>Ziprasidone<br>Zoniporide<br>Internal standard: Tolbutamide	413 > 382<br>416 > 176<br>427 > 370<br>306 > 236<br>413 > 194<br>321> 262<br>271>91	176<br>151<br>176<br>151<br>141<br>96<br>106	33<br>35<br>37<br>37<br>39<br>19<br>43	0.71<br>0.87<br>0.54<br>0.89<br>0.78<br>0.66<br>0.98
**Cytosolic incubations**	Sciex 6500+ Triple Quad equipped with Waters Acquity UPLC H-Class (Waters Corporation, USA)	Waters ACQUITY UPLC BEH C18 Column, 130Å, 1.7 µm, 2.1 mm x 50 mm, eluent A: 10 mM ammonium formate + 0.1% formic acid in water, eluent B: acetonitrile; gradient elution: flow 0.75 mL/min, 0 min – 90% A, 0.47 min – 5% A, 0.65 min – 5% A, 0.66 min – 90% A, 1 min – 90% A	The ratio of the peak areas of analyte and internal standard	Capmatinib<br>Idelalisib<br>Lenvatinib<br>Zaleplon<br>Ziprasidone<br>Zoniporide<br>Internal standard: Pruvanserin	413.3>382.2<br>416.2>176.1<br>427.3>370.1<br>306.0>236.1<br>413.1>194.1<br>321.1>262.0<br>377.14>209.4	88<br>65<br>80<br>85<br>84<br>54<br>56	35<br>39<br>37<br>39<br>38<br>23<br>31	0.46<br>0.51<br>0.45<br>0.51<br>0.47<br>0.40<br>0.47
**Cytosolic incubations-Site 2**	Sciex QTRAP6500 MS equipped with Waters Acquity i-Class UPLC	Waters Acquity UPLC Acquity HSS T3 column (30x2.1mmx1.8µm); Mobile Phase A1: Acetonitrile; Mobile Phase B1: 10mMAmmonium Formate+0.1%Formic Acid; Flow rate: 0.4 ml/min; Injection volume: 10µl; Weak solvent: Water with 10% Acetonitrile + 0.1% Formic Acid; Strong solvent: Acetonitrile; Gradient: 1.2 min in gradient elution changing percentage of Acetonitrile and Ammonium Formate.	The ratio of the peak areas of analyte and internal standard	Capmatinib<br>Idelalisib<br>Lenvatinib<br>Ziprasidone<br>Zoniporide<br>Internal standard: Midazolam	413.3>382.1<br>416.1>176.1<br>427.1>370.0<br>413.1>194.0<br>321.1>303.1<br>326.1>291	90<br>80<br>90<br>90<br>80<br>96	37<br>36<br>39<br>39<br>29<br>37	0.63<br>0.66<br>0.62<br>0.63<br>0.77<br>0.78

**Microsomal incubations**	SCIEX Triple Quad 5500 MS equipped with Agilent 1290 Infinity Binary Pump (Santa Clara, CA, USA)	Waters X-Bridge C8 30 x 2.1 mm 5µ column, Water (0. 1% Formic acid):ACN in gradient elution: flow 0.8 mL/min, 0 min – 95% A, 0.3 min – 2% A, 0.8 min – 95% A, 1 min – 95% A	The ratio of the peak areas of analyte and internal standard	Capmatinib	413.2>354.133	65	45	0.43
				Idelalisib	416.1>203.948	65	77	0.45
				Lenvatinib	427.1>282.972	65	65	0.42
				Zaleplon	306.1>219.079	65	45	0.48
				Ziprasidone	413.2>166.011	65	62	0.46
				Zoniporide	322.2>234.1	65	30	0.41
				Internal standard: Carbutamide	272.2/108.1	65	35	0.49
**Cytosolic binding**	Sciex Q-Trap 6500+ mass spectrometer equipped with Shimadzu Nexera X2 UPLC	Kinetex C18 column (2.6 mm, 100Å, 30 x 2.1 mm) (Torrance, CA).<br><br>Water (0. 1% Formic acid):ACN in gradient elution: flow 1.0 mL/min, 0 min-97% A, 0.3 min – 97% A, 1.3 min – 70% A, 2.3 min- 5% A, 2.8 min- 5% A, 3.55 min-97% A, 4.3 min- 97% A	The ratio of the peak areas of analyte and internal standard	Capmatinib	413.2->381.9	90	37	1.08
				Idelalisib	416.2->176.0	80	36	1.31
				Lenvatinib	427.2->369.9	90	39	1.08
				Zaleplon	306.2->236.0	90	39	1.38
				Ziprasidone	413.1->194.0	90	39	1.24
				Zoniporide	413.1->194.0	50	22	0.93
				Internal standard: Propranolol	260.1->118.1	60	27	1.19
**Microsomal binding**	API Sciex 5000 coupled with CTC HTS PAL (Zwingen, Switzerland) and Agilent 1100/1290	Kinetex XB 2.6µ C18 100Å (50 x 2.1 mm ID)<br>5mM Ammonium Acetate 0.75% formic acid:0.1%formic ACN	The ratio of the peak areas of analyte and internal standard	Capmatinib	413 > 382	100	34	0.70
				Idelalisib	416 > 176	100	35	0.77
				Lenvatinib	427 > 370	70	37	0.67
				Zaleplon	306 > 236	80	37	0.77
				Ziprasidone	413 > 194	55	39	0.70
				Zoniporide	321 > 262	50	21	0.61
				Internal standard: Tolbutamide	271 > 172	60	12	0.83

Supplement 2. Details of PBPK model development (absorption and distribution)

Molecular weight (MW), the octanol–water partition coefficient (logP), and the acid dissociation constant (pKa) were collated from the literature. Unbound fraction in plasma (fu_p) and blood-to-plasma ratio (B/P) were measured. The main plasma-binding protein was assumed to be albumin for ampholyte and neutral compounds, and α-1 acid glycoprotein for the basic compounds. For compounds administrated orally, first-order absorption model parameters (e.g. absorption rate constant [ka], fraction absorbed [fa], and a nominal flow in the gut model [Q_{gut}]) were predicted from *in vitro* permeability data using in-house apparent-effective permeability (P_{app}–P_{eff}) correlation (Supplement 1, Eq.S8)[3, 4] or estimated by fitting the model to *in vivo* data (See Supplement 3). Lag time (t_{lag}), was adjusted to fit the observed data, when necessary. In model fitting, weighted least-squares regression was performed using the Nelder-Mead algorithm (maximum of 500 iterations). Unbound fraction in gut (fu_{gut}) was assumed to be equal to fu_p.[4]

Tissue:plasma partition coefficients (Kp) were predicted via Rodgers and Rowlands[5] method, or Poulin-Theil/Berezhkovskiy method[6, 7] (for lipophilic compounds) using whole-body PBPK model using a global scalar for all Kp to recover the observed volume of distribution at steady state (V_{ss}). *In vivo* clearance was fixed and simulated profiles were compared with observed data to verify absorption and distribution components. In cases, where full PBPK model could not predict C_{max} and AUC_{inf} within 2-fold, a minimal PBPK model was used representing a non-physiological central compartment excluding the liver and portal vein coupled with a non-physiological non-central compartment, facilitating adjustment of systemic compartment concentration profile (SAC). When necessary, the volume of distribution at steady state (V_{ss}), volume of SAC (V_{sac}) and the inter-compartment clearance in compartmental modelling (Q), were estimated by fitting the models to observed pharmacokinetic data. Once absorption and distribution parameters were optimized, bottom-up predictions of AO-mediated pharmacokinetics using unbound intrinsic clearance (CL_{int}) data from i*n vitro* human liver microsomes and cytosols (generated) or human hepatocytes (literature data) were utilized.

Supplement 3. *In vivo* pharmacokinetic and human mass balance data

Content:

Supp. Table 2. Fraction metabolised (fm/fe) values obtained from human mass balance studies

Suppl. Table 3. *In vivo* pharmacokinetic and DDI studies used in PBPK model development, refinement, and verification

Table S2. Fraction metabolised (fm/fe) values obtained from human mass balance studies

Compound	fm_{AO}	fm_{CYP3A4}	fm_{other}	fe_{renal}	Ref	Comments
**Capmatinib**	0.40	0.6	-	-	[8]	Measured in excreta and assuming negligible contribution of xanthine oxidase<br>Assuming renal clearance is negligible
**Idelalisib**	0.29	0.12, 0.68	0, 0.56	0.03	[9, 10]	Measured in excreta, uncertainty in fm_{CYP3A4} due to unidentified metabolic pathways<br>Assuming negligible contribution of UGTs (0.03)
**Lenvatinib**	0.17, 0.26	0.28, 0.41	0.33, 0.54	0.004	[11]	Measured in excreta, uncertainty due to low recovery of metabolites<br>Studied in cancer population
**Zaleplon**	0.57, 0.74	0.26, 0.43	-	-	-	Measured in excreta assuming secondary metabolite is formed from AO or CYP3A4 metabolite<br>Assuming renal clearance is negligible
**Ziprasidone**	0.67	0.33	-	-	[12]	Measured in excreta and ignoring unquantified contribution of GSH-mediated chemical reduction<br>Assuming renal clearance is negligible
**Zoniporide**	0.52, 0.69	-	0.13, 0.30	0.17; 0.01 (biliary)	[13]	Measured in excreta assuming secondary metabolite is formed from AO or CYP3A4 metabolite

Table S3. ***In vivo*** **pharmacokinetic and DDI studies used in PBPK model development, refinement, and verification**

	Aim	Disease State	Dose (mg)	Dose of perpetrator	Route of administration	Number of subjects	Number of trials	Age range	Female fraction	Ref.
**Capmatinib**										
**Model development**	Hepatic impairment	Healthy	200	-	Oral	9	20	43-62	0.2	14
**Model development**	ADME	Healthy	600	-	Oral	6	20	46-58	0	8
**Model verification**	CYP inhibition (itraconazole)	Healthy	200	200 (MD)	Oral	23	10	21-55	0	15
	CYP induction (rifampicin)		400	600 (MD)	Oral	24	10	27-55	0.192	
**Idelalisib**										
**Model development**	Hepatic impairment	Healthy	150	-	Oral	10	10	35-65	0.33	16
**Model verification**	CYP inhibition[a] (ketoconazole)	Healthy	400	400 (MD)	Oral	11	10	18-65	0	10, 17
**Model verification**	CYP induction (rifampicin)	Healthy	150	600 (MD)	Oral	11	10	28-54	0.4	18
**Lenvatinib**										
**Model development**	Hepatic impairment	Healthy	10	-	Oral	8	20	50-61	0.375	19
**Model verification**	CYP inhibition (ketoconazole)	Healthy	5	400 (MD)	Oral	16	10	19-53	0.17	20
**Model verification**	CYP induction[b] (rifampicin)	Healthy	24	600 (MD)	Oral	15	10	20-49	0.27	21
**Zaleplon**										
**Model development**	Pharmacokinetics	Healthy	5	-	iv and oral	10	10	19-32	0 or 1	22
**Ziprasidone**										
**Model development**	Pharmacokinetics	Healthy	5 (iv), 20 (oral)	iv	iv and oral	12 (iv), 13 (oral)	10	19-37	0	23
**Model verification**	CYP inhibition (ketoconazole)	Healthy	40	200-400 (MD)	Oral	13	10	18-31	0.54	24
**Model verification**	CYP induction (carbamazepine)	Healthy	20	100-200 (MD)	Oral	9	20	23-35	0.1	25
**Zoniporide**										
**Model development**	Pharmacokinetics, ADME	Healthy	80	-	IV	4	25	18-55	0	13

Abbreviations: iv, intravenous.

[a]Idelalisib DDI study with ketoconazole was conducted with the idelalisib dose higher than the dose-linearity range (400 mg), simulations were performed with the age range of 18-65 years as no information was available.[10, 17]

[b]P-gp transporter kinetic parameters for lenvatinib (substrate) were not available. Thus, simulations of idelalisib DDI study with rifampicin do not reflect P-gp interaction [21]

Zaleplon DDI study with ketoconazole was not used as study details were not available.

Supplement 4. References for Simcyp v21 default compound files

Itraconazole:

- Chen Y, Ma F, Lu T, Budha N, Jin JY, Kenny JR, Wong H, Hop CE, Mao J (2016) Development of a Physiologically Based Pharmacokinetic Model for Itraconazole Pharmacokinetics and Drug-Drug Interaction Prediction. Clinical Pharmacokinetics 55(6) 735-749.
- Chen Y, Cabalu TD, Callegari E, Einolf H, Liu L, Parrott N, Peters SA, Schuck E, Sharma P, Tracey H, Upreti VV, Zheng M, Zhu AZX, Hall SD (2019) Recommendations for the Design of Clinical Drug-Drug Interaction Studies with Itraconazole using a Mechanistic PBPK Model. CPT: Pharmacometrics & Systems Pharmacology 8(9) 685-695.
- Dickinson GL, Phillips DL, Posada MM, Chaudhary A, Hall SD (2017) Physiologically based pharmacokinetic modeling to understand the observed drug–drug interaction of LY2623091 with CYP3A inhibitors itraconazole and diltiazem. International Journal of Pharmacokinetics 2(4).
- Fotaki N and Klein S (2013) Mechanistic understanding of the effect of PPIs and acidic carbonated beverages on the oral absorption of itraconazole based on absorption modeling with appropriate in vitro data. Molecular Pharmaceutics 10(11):4016-4023.
- Gardin A, Shakeri-Nejad K, Feller A, Huth F, Neelakantham S, Dumitras S (2019) Siponimod pharmacokinetics, safety, and tolerability in combination with the potent CYP3A4 inhibitor itraconazole in healthy subjects with different CYP2C9 genotypes. European Journal of Clinical Pharmacology 75(11) 1565-1574.
- Isoherranen N, Kunze KL, Allen KE, Nelson WL, Thummel KE (2014) Role of itraconazole metabolites in CYP3A4 inhibition.Drug Metabolism and Disposition 32(10):1121-1131.
- Jaminion F, Bentley D, Wang K, Wandel C, Derks M, Diack C (2020) PKPD and cardiac single cell modeling of a DDI study with a CYP3A4 substrate and itraconazole to quantify the effects on QT interval duration. Journal of Pharmacokinetics and Pharmacodynamics 47(5) 447-459.
- Ke AB, Zamek-Gliszczynski MJ, Higgins JW, Hall SD (2014) Itraconazole and clarithromycin as ketoconazole alternatives for clinical CYP3A inhibition studies. Clinical Pharmacology and Therapeutics 95(5) 473-476.
- Prieto Garcia L, Janzén D, Kanebratt KP, Ericsson H, Lennernäs H, Lundahl A (2018) Physiologically Based Pharmacokinetic Model of Itraconazole and Two of Its Metabolites to Improve the Predictions and the Mechanistic Understanding of CYP3A4 Drug-Drug Interactions. Drug Metabolism & Disposition 46(10) 1420-1433.
- Riddell K, Patel A, Collins G, Zhou Y, Schramek D, Kremer BE, Ferron-Brady G (2021) An Adaptive Physiologically Based Pharmacokinetic-Driven Design to Investigate the Effect of Itraconazole and Rifampicin on the Pharmacokinetics of Molibresib (GSK525762) in Healthy Female Volunteers. Journal of Clinical Pharmacology 61(1) 125-137.

Ketoconazole:

- Boulenc X, Nicolas O, Hermabessière S, Zobouyan I, Martin V, Donazzolo Y, Ollier C (2016) CYP3A4-based drug-drug interaction: CYP3A4 substrates' pharmacokinetic properties and ketoconazole dose regimen effect. European Journal of Drug Metabolism and Pharmacokinetics 41(4) 45-54.
- Cristofoletti R, Charoo NA, Dressman JB (2016b) Exploratory Investigation of the Limiting Steps of Oral Absorption of Fluconazole and Ketoconazole in Children Using an In Silico Pediatric Absorption Model. Journal of Pharmaceutical Sciences 105(9):2794-2803.
- Johansson S, Löfberg B, Aunes M, Lunde H, Frison L, Edvardsson N, Cullberg M (2016) In Silico Predictions and In Vivo Results of Drug-Drug Interactions by Ketoconazole and Verapamil on AZD1305, a Combined Ion Channel Blocker and a Sensitive CYP3A4 Substrate. Clinical Pharmacology in Drug Development 5(5) 364-373.

- Ke AB, Zamek-Gliszczynski MJ, Higgins JW, Hall SD (2014) Itraconazole and clarithromycin as ketoconazole alternatives for clinical CYP3A inhibition studies. Clinical Pharmacology and Therapeutics 95(5) 473-476.
- Pathak S, Ruff A, Kostewicz ES, Patel N, Turner DB, Jamei M (2017) Model-based Analysis of Biopharmaceutical Experiments to Improve Mechanistic Oral Absorption Modelling - An Integrated in Vitro in Vivo Extrapolation (IVIV_E) Perspective using Ketoconazole as a Model Drug. Mol Pharmaceut 14(12): 4305-4320.
- Pithavala YK, Tong W, Mount J, Rahavendran SV, Garrett M, Hee B, Selaru P, Sarapa N, Klamerus KJ (2012) Effect of ketoconazole on the pharmacokinetics of axitinib in healthy volunteers. Investigational New Drugs 30(1) 273-281.
- Yang Z, Vakkalagadda B, Shen G, Ahlers CM, Has T, Christopher LJ, Kurland JF, Roongta V, Masson E, Zhang S (2013) Inhibitory effect of ketoconazole on the pharmacokinetics of a multireceptor tyrosine kinase inhibitor BMS-690514 in healthy participants: assessing the mechanism of the interaction with physiologically-based pharmacokinetic simulations. Journal of Clinical Pharmacology 53(2) 217-227.
- Zhao P, Ragueneau-Majlessi I, Zhang L, Strong JM, Reynolds KS, Levy RH, Thummel KE, Huang SM (2009) Quantitative evaluation of pharmacokinetic inhibition of CYP3A substrates by ketoconazole: a simulation study. Journal of Clinical Pharmacology 49(3) 351-359.

Rifampicin:

- Agrawal S and Panchagnula R (2005) Implication of biopharmaceutics and pharmacokinetics of rifampicin in variable bioavailability from solid oral dosage forms. Biopharmaceutics and Drug Disposition 26(8):321-334.
- Badhan RKS, Gittins R, Al Zabit D (2019) The optimization of methadone dosing whilst treating with rifampicin: A pharmacokinetic modeling study. Drug and Alcohol Dependence 200: 168-180.
- Dawson J, Dedigama M, Elliot DJ, Sorich M, Mangoni AA, Rowland A (2016) Prolonged induction of warfarin metabolism and a paradoxical INR response in a mitral valve replacement patient receiving rifampicin for infective endocarditis. Biomedical Research and Clinical Practice 1(2) 62-65.
- Gandelman K, Zhu T, Fahmi OA, Glue P, Lian K, Obach RS, Damle B (2011) Unexpected effect of rifampin on the pharmacokinetics of linezolid: in silico and in vitro approaches to explain its mechanism. Journal of Clinical Pharmacology 51: 229-236.
- Kapetas AJ, Sorich MJ, Rodrigues AD, Rowland A (2019) Guidance for Rifampin and Midazolam Dosing Protocols To Study Intestinal and Hepatic Cytochrome P450 (CYP) 3A4 Induction and De-induction. The AAPS Journal 21 (5) 78.
- Olafuyi O, Coleman M, Badhan RKS (2017) Development of a paediatric physiologically based pharmacokinetic model to assess the impact of drug-drug interactions in tuberculosis co-infected malaria subjects: A case study with artemether-lumefantrine and the CYP3A4-inducer rifampicin. European Journal of Pharmaceutical Sciences 106: 20-33.
- Pahwa S, Alam K, Crowe A, Farasyn T, Neuhoff S, Hatley O, Ding K, Yue W (2017) Pretreatment With Rifampicin and Tyrosine Kinase Inhibitor Dasatinib Potentiates the Inhibitory Effects Toward OATP1B1- and OATP1B3-Mediated Transport. Journal of Pharmaceutical Sciences 106(8) 2123-2135.
- Rasool MF, Khalid S, Majeed A, Saeed H, Imran I, Mohany M, Al-Rejaie SS, Alqahtani F (2019) Development and Evaluation of Physiologically Based Pharmacokinetic Drug-Disease Models for Predicting Rifampicin Exposure in Tuberculosis and Cirrhosis Populations. Pharmaceutics 11(11):578.

- Rekić D, Röshammar D, Mukonzo J & Ashton M (2011) In silico prediction of efavirenz and rifampicin drug-drug interaction considering weight and CYP2B6 phenotype. British Journal of Clinical Pharmacology 71(4) 536-543.
- Riddell K, Patel A, Collins G, Zhou Y, Schramek D, Kremer BE, Ferron-Brady G (2021) An Adaptive Physiologically Based Pharmacokinetic-Driven Design to Investigate the Effect of Itraconazole and Rifampicin on the Pharmacokinetics of Molibresib (GSK525762) in Healthy Female Volunteers. Journal of Clinical Pharmacology 61(1) 125-137.
- Varma M, Lin J, Bi Y-A, Rotter C, Fahmi O, Lam JL, El-Kattan AF, Goosen TC Lai Y (2013) Quantitative Prediction of repaglinide-rifampicin complex drug interactions using dynamc and static mechanistic models: delineating differential CYP3A4 induction and OATP1B1 inhibition potential of rifampicin. Drug Metabolism and Disposition 41: 966-974.
- Westphal K, Weinbrenner A, Zschiesche M, Franke G, Knoke M, Oertel R, Fritz P, von Richter O, Warzok R, Hachenberg T, Kauffmann HM, Schrenk D, Terhaag B, Kroemer HK, Siegmund W (2000) Induction of P-glycoprotein by rifampin increases intestinal secretion of talinolol in human beings: a new type of drug/drug interaction. Clinical Pharmacology and Therapeutics 68:345-355.
- Yamazaki S, Costales C, Lazzaro S, Eatemadpour S, Kimoto E, Varma MV (2019) Physiologically-Based Pharmacokinetic Modeling Approach to Predict Rifampin-Mediated Intestinal P-Glycoprotein Induction.CPT Pharmacometrics and Systems Pharmacology 8(9) 634-642.

Carbamazepine:

- Schuck E, Ferry J, Gidal B, Hussein Z (2020) Changes in perampanel levels during de-induction: Simulations following carbamazepine discontinuation. Acta Neurologica Scandinavica 142(2) 131-138.

Supplement 5. Exploratory analysis to evaluate potential AO-mediated DDI risks

Content:

Semi-mechanistic model for AO-mediated DDI risk predictions

Supp. Table 4. Summary of inhibitor parameters used in the semi-mechanistic static model to evaluate the risk of AO-mediated DDIs

Semi-mechanistic model for AO mediated DDI risk predictions:

The potential risk of AO-mediated DDI by ethinyl estradiol, raloxifene, and erlotinib was explored using the semi-mechanistic static model with listed parameters in Suppl. Table 4. The most conservative approach is used for all compounds with maximum $C_{max,u}$ and minimum $K_{i,u}$ data available.

The semi-mechanistic static model was utilized to predict the AUCR AO substrates with varying fm_{AO} (0.1-1) via Eq. S14-16[26] by only considering the interaction via hepatic AO. For erlotinib and icotinib, both reversible inhibition and time-dependent inhibition were considered.

$$AUCR\ [+inhibitor/control]) = \frac{1}{[A_h \times B_h] \times fm + (1 - fm)}$$ Eq. S14

$$Reversible\ inhibition\ (A_h) = \frac{1}{1 + \frac{[I]}{K_i}}$$ Eq. S15

$$Time - dependent\ inhibition\ (B_h) = \frac{k_{deg}}{k_{deg} + \frac{[I] \times k_{inact}}{[I] + K_{I,u}}}$$ Eq. S16

[I] is the unbound concentration of inhibitors in the liver and was designated as the unbound maximum systemic concentration of inhibitors as a surrogate.[27] Due to the lack of AO k_{deg} values in the literature, the arithmetic means of all reported P450 k_{deg} (0.00026 min^{-1}) was used.[28, 29]

Table S4. Summary of inhibitor parameters used in the semi-mechanistic static model to evaluate the risk of AO-mediated DDIs

Parameter	Cimetidine	Ethinyl estradiol	Raloxifene	Erlotinib	Icotinib
Inhibition constant (K_i) (nM)[a]	155000[30]	1100[31]	0.87-51	140[32]	359[32]
Unbound fraction in incubation to calculate $K_{i,u}$ (fu_{inc})	0.85[a]	0.94[c 31, 33, 34]	0.83[d 35, 36]	0.958[29]	0.98[e 32]
Time-dependent inhibition constant (K_I) (nM)	-	-	-	719[32]	1180[32]
fu_{inc} to calculate $K_{i,u}$	-	-	-	0.693[29]	0.78[f 32]
Maximal inactivation rate (k_{inact}) (1/min)	-	-	-	0.360[32]	0.546[32]
Dose (mg)	300[37]	0.05 (QD)[38]	60 (QD)[39]	150 (QD)[40]	200 (BID)[41]
Maximum plasma concentration (C_{max}) (nM)	6251[37]	0.607[38]	2.86[39]	6060[40]	5158[41]
Unbound fraction in plasma (fu_p)	0.8[b]	0.015[42]	0.05[43]	0.07[29]	0.11[44]
Molecular weight (g/mol)	252[33]	296[42]	510[43]	393[33]	391[33]

[a]Predicted fu_{inc} at 2 mg/mL cytosolic protein from logP data[33, 34]

[b]Simcyp v.21 Compound Repository

[c]Predicted fu_{inc} at 0.05 mg/mL cytosolic protein from logP data[33, 34]

[d]Corrected for the 0.05 mg/mL cytosolic protein from the measured fu_{inc} at 1.5 mg/mL cytosolic protein[34, 36]

[e]Predicted fu_{inc} at 0.06 mg/mL cytosolic protein from logP data[33, 34]

[f]Predicted fu_{inc} at 0.6 mg/mL cytosolic protein from logP data[33, 34]

Supplement 6. Experimental results

Content:

Suppl. Table 5. Generated plasma binding data for study and reference compounds

Suppl. Table 6. Generated blood-to-plasma ratio data for study and reference compounds

Suppl. Table 7. Generated *in vitro* permeability data (cMdr1-KO MDCK-EV) for study and reference compounds

Suppl. Table 8. Generated *in vitro* cytosolic and microsomal intrinsic clearance ($CL_{int,\ in\ vitro}$) data for study compounds

Suppl. Figure 1. Substrate depletion profiles obtained in human liver cytosols (HLC) and microsomes (HLM) incubations for study compounds a) capmatinib, b) idelalisib, c) lenvatinib, d) zaleplon, e) ziprasidone, f) zoniporide.

Suppl. Table 9. Generated cytosolic and microsomal binding data for study compounds

Table S5. Generated plasma binding data for study and reference compounds

	fu_p[a] (6 h incubation)	% Recovery	% Stability
	(n=3; mean, CV%)		(n=1; at 20h)
**Capmatinib**	0.0516 (28.6)	94.8 (4.42)	78.8
**Idelalisib**	0.0536 (13.6)	123 (8.90)	96.2
**Lenvatinib**	0.0247 (4.59)	119 (6.53)	108
**Zaleplon**	0.569 (14.4)	95.0 (1.67)	104
**Ziprasidone**	0.0029 (12.6)	124 (7.48)	96.2
**Zoniporide**	0.288 (11.8)	107 (13.2)	105
**Warfarin (reference)**	0.0064 (1.26)	107 (1.94)	101
**Propranolol (reference)**	0.137 (4.98)	96.1 (3.65)	94.8

[a] Mean values were used as PBPK model input parameters

Table S6. Generated blood-to-plasma ratio data for study and reference compounds

	Concentration of compounds (ng/mL)	B/P[a]	Mean (CV%)[b]
**Capmatinib**	10	3.95	Concentration dependent B/P profile
	50	3.58 (28.4)	
	1000	2.56 (24.6)	
	5000	1.370	
**Idelalisib**	50	0.740	0.700 (8.1)
	1000	0.660	
**Lenvatinib**	50	0.640	0.645 (1.1)
	1000	0.650	
**Zaleplon**	10	0.99	0.91 (9.8)
	50	0.98 (19.7)	
	250	0.98	
	1000	1	
**Ziprasidone**	50	0.44	0.455 (4.7)
	1000	0.47	
**Zoniporide**	50	0.84	0.86 (3.3)
	1000	0.88	
**Verapamil (reference)**	50	0.75	n/a
	1000	0.78	
**Chloroquine (reference)**	50	3.46	n/a
	1000	2.40	

[a] Mean (% CV) values from duplicate measurements

[b] Mean values were used as PBPK model input parameters

Table S7. Generated *in vitro* permeability data (cMdr1-KO MDCK-EV) for study and reference compounds

	P_{app}[b] (x 10^{-6} cm/s) Apical to basolateral	% Recovery	Papp (x 10^{-6} cm/s) Basolateral to apical	% Recovery	Efflux ratio[d]
			(n=3; mean, CV%)		
**Capmatinib**	32.0 (1.88)	79.6 (8)	29.5 (3.05)	82.2 (5)	0.921
**Idelalisib**	16.2 (11.7)	89.6 (3.7)	14.7 (2.04)	86.6 (2.7)	0.907
**Lenvatinib**	29.8 (7.38)	88.6 (12.7)	21.3 (14.6)[c]	93.0 (5.4)	0.715
**Zaleplon**	38.9 (1.80)	89.3 (1.9)	32.5 (9.54)	90.8 (5.3)	0.836
**Ziprasidone**	3.15 (3.81)	62.9 (6.7)	0.843 (5.81)	61.1 (5.7)	0.268
**Zoniporide**	1.72 (25.0)	66.2 (24)	1.22 (18.0)	64.5 (6.9)	0.708
**3H-atenolol**[a]	1.47 (25.2)	n/a	1.45 (8.97)	n/a	0.985
**3H-propranolol**[a]	31.4 (4.14)	n/a	17.7 (9.6)	n/a	0.563
**3H-digoxin**[a]	1.66 (34.3)	n/a	1.43 (28)	n/a	0.864
**14C-mannitol**[a]	0.793 (76.4)	n/a	0.378 (63.2)	n/a	0.476

[a]Control compound

[b]Mean values were used as PBPK model input parameters

[c]n=2 due to mannitol >3 x 10^6 cm/s in one replicate

[d]Ratio of mean values

Table S8. Generated *in vitro* cytosolic and microsomal intrinsic clearance ($CL_{int,\ in\ vitro}$) data for study compounds.

	$CL_{int,\ in\ vitro}$ (µL/min/mg cytosolic protein) Site-1<br>*Triplicate*	$CL_{int,\ in\ vitro}$ with 50 µM hydralazine* (µL/min/mg cytosolic protein) Site-1<br>*Triplicate*	$CL_{int,\ in\ vitro}$ (µL/min/mg cytosolic protein) Site-2<br>*Single replicate*	$CL_{int,\ in\ vitro}$[e] (µL/min/mg cytosolic protein) (Mean, CV%)	$CL_{int,\ in\ vitro}$[e] (µL/min/mg microsomal protein)<br>*Duplicate*
**Capmatinib**	3.80[a]	<LOQ[c]	4.27	4.04 (8.34)	30.4
**Idelalisib**	3.27	<LOQ[c]	4.37	3.82 (20.4)	9.73
**Lenvatinib**	0.982[b]	<LOQ[d]	<LOQ[c]	0.982	7.43[a]
**Zaleplon**	4.12	<LOQ[c]	n/a	4.12	5.95
**Ziprasidone**	4.67	3.92[b]	<LOQ[c]	4.67	71.9
**Zoniporide**	20.0	3.54[b]	13.9	16.5 (21.7)	5.19[a]

[a]One replicate showed more than 15% substrate depletion (10% for lenvatinib and zoniporide) at the end of incubation

[b]Two replicates showed more than 15% substrate depletion at the end of incubation

[c]Less than 15% substrate depletion (10% for lenvatinib) at the end of the incubation period

[d]Less than 1% substrate depletion at 60 min and 6%-13% at 120 min.

[e]Mean value from site-1 and 2 was used as PBPK model input parameter

*The contribution of other cytosolic enzymes (e.g. XO) was ignored for most compounds because substrate depletion was unquantifiable or negligible (zoniporide). For ziprasidone, hydralazine only caused a modest inhibition (17%). Although it was known that ziprasidone also undergoes glutathione (GSH)-mediated chemical reduction, it was challenging to disseminate two pathways, as reports show another AO inhibitor menadione (100 µM) caused almost complete inhibition (90%) in contrast to hydralazine (12%).[45] Therefore the GSH-mediated reduction was ignored. The lack of enzyme activity was also apparent in recombinant systems with high variability between different systems with up-to 6-fold lower activity in recombinant systems compared to HLC[46] with variable relative expression factor (HLC vs. recombinant AO) ranging between 0.001-1.7.[46] Therefore, confidence remains low to gauge recombinant AO in fm_{AO} predictions.

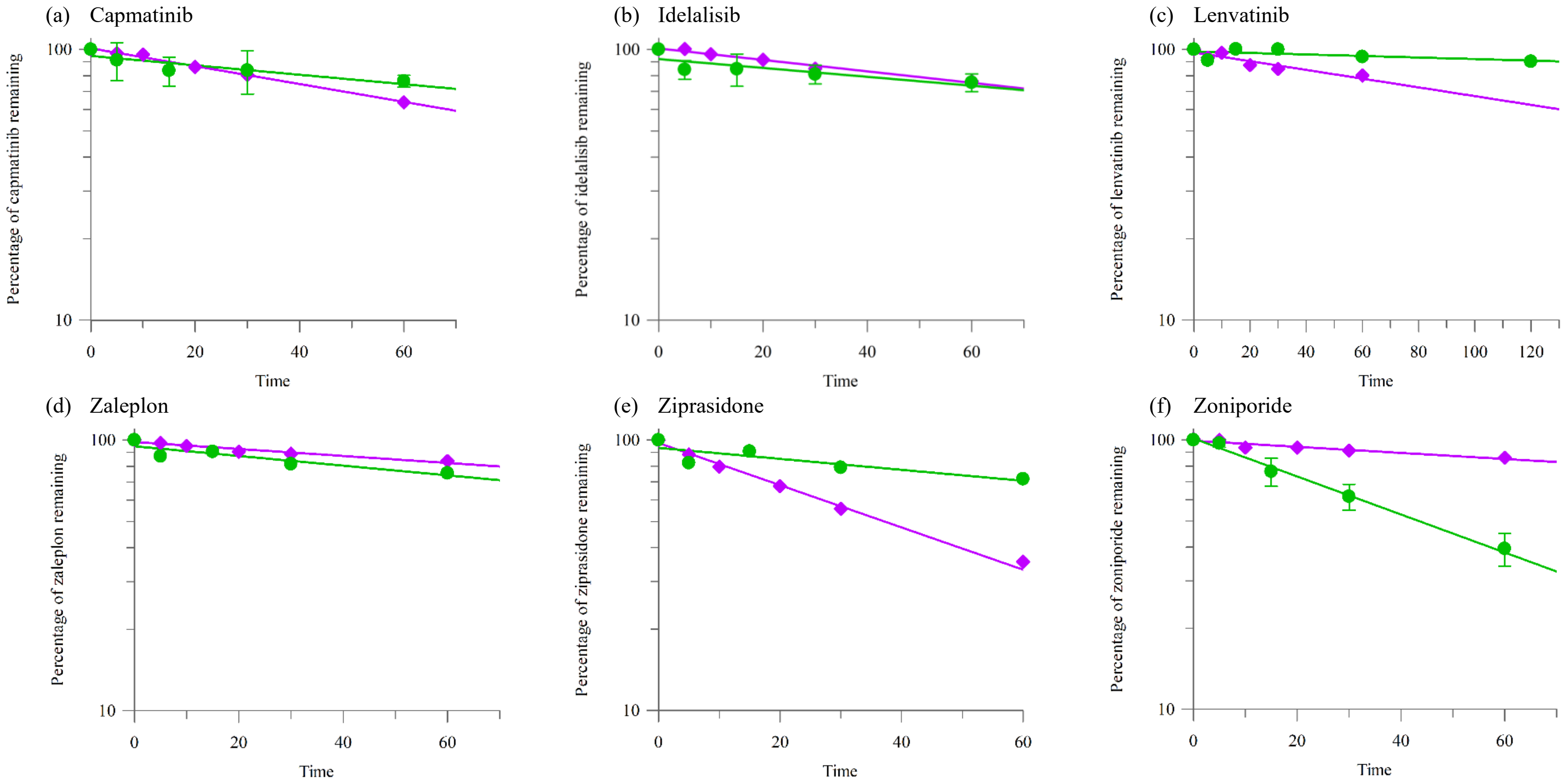

Figure S1. Substrate depletion profiles obtained in human liver cytosols (HLC; green circles) and microsomes (HLM; purple diamonds) incubations for study compounds a) capmatinib, b) idelalisib, c) lenvatinib, d) zaleplon, e) ziprasidone, f) zoniporide.

Table S9. Generated cytosolic and microsomal binding data for study compounds

	Experimental fu_{cyt} (mean, CV%) [1 mg/mL; n=3]				Experimental fu_{mic} (mean, CV%) [0.25 mg/mL; n=4]	Predicted fu_{mic} for 0.5 mg/mL[b]	Predicted fu_{mic} for 1 mg/mL[b]
	Dog Liver Cytosol[a]	Dog Liver Cytosol with 50 uM Hydralazine	Human Liver Cytosol with 50 uM Hydralazine	Human Liver Cytosol with 5 uM Raloxifene			
**Capmatinib**	0.461 (8.67)	0.718 (2.47)	0.831 (2.65)	0.777 (8.48)	0.826 (4.83)[a]	0.707	0.549
**Idelalisib**	0.523 (14.5)	0.814 (5.11)	0.924 (3.35)	0.787 (5.70)	0.897 (10.13)	0.828[a]	0.726
**Lenvatinib**	0.331 (7.16)	0.721 (7.47)	0.718 (1.95)	0.678 (3.49)	0.764 (10.25)	0.627[a]	0.463
**Zaleplon**	1 (7.56)	0.84 (3.63)	1 (1.10)	0.846 (1.87)	0.981 (1.80)	0.964[a]	0.932
**Ziprasidone***	0.098 (6.02)	0.288 (3.81)	0.356 (2.53)	0.337 (4.82)	0.210 (9.29)[a]	0.118	0.063
**Zoniporide**	0.664 (19.2)	0.847 (1.57)	1 (0.40)	0.954 (5.40)	0.778 (7.94)	0.642[a]	0.476

[a] Mean values used as PBPK model input parameter

[b]Corrected for protein concentration using Hallifax&Houston algorithm [34] using experimental fu_{mic} at 0.25 mg/mL

Supplement 7. Observed vs. predicted pharmacokinetics from individual simulations

Content:

Suppl. Figure 2. Observed vs. predicted pharmacokinetics by the bottom-up model from human liver microsomes and cytosols

Suppl. Figure 3. Observed vs. predicted pharmacokinetics by the bottom-up model from hepatocytes

Suppl. Figure 4. Observed vs. predicted pharmacokinetics by the bottom-up model with empirical scaling factors from human liver microsomes and cytosols

Suppl. Figure 5. Observed vs. predicted pharmacokinetics by the bottom-up model with empirical scaling factors from human hepatocytes

Suppl. Figure 6. Observed vs. predicted pharmacokinetics by using the middle-out model from human liver microsomes and cytosols with observed fraction metabolized

Suppl. Figure 7. Observed vs. predicted pharmacokinetics by using the middle-out model from human hepatocytes with observed fraction metabolized

Suppl. Figure 8. Observed vs. predicted pharmacokinetics by the middle-out model from human liver microsomes and cytosols with observed intrinsic clearance data

Suppl. Figure 9. Observed vs. predicted pharmacokinetics by the middle-out model from human hepatocytes with observed intrinsic clearance data

Suppl. Figure 10. Observed vs. predicted pharmacokinetics by the top-down model with observed fraction metabolized data and observed intrinsic clearance data

Suppl. Figure 11. Observed vs. predicted fraction eliminated

Suppl. Table 10. Summary of observed vs. predicted pharmacokinetic parameters of study compounds with bottom-up PBPK models using the data from human liver microsomes and cytosols and refined models.

Suppl. Table 11. Summary of observed vs. predicted pharmacokinetic parameters of study compounds with bottom-up PBPK models using the data from human hepatocytes (with and without hydralazine) and refined models.

Suppl. Table 12. Evaluation of model performances

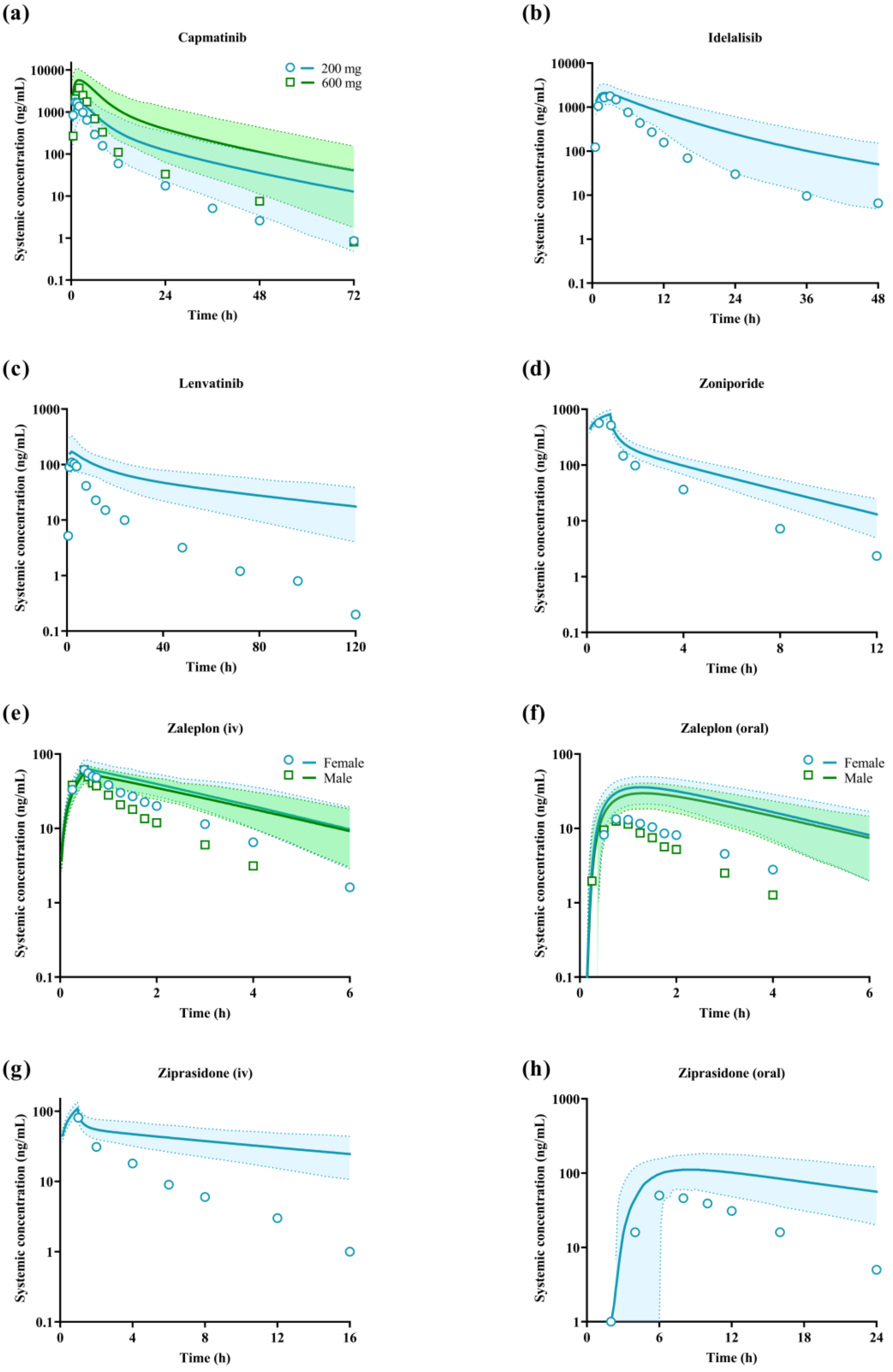

Figure S2. Simulation of the pharmacokinetics using the bottom-up model from human liver microsomes and cytosols of a) capmatinib [oral; 200 and 600 mg], b) idelalisib [oral; 150 mg], c) lenvatinib [oral; 10 mg], d) zoniporide [iv infusion; 80 mg], e) zaleplon [iv infusion; 5 mg in female and male], f) zaleplon [oral; 5 mg in female and male], g) ziprasidone [iv infusion; 5 mg], h) ziprasidone [oral; 20 mg]. Solid lines represent the predicted profile. Symbols (circle and square) represent observed data, if available. Area between the dotted lines representing the 5th and 95th percentile of predictions is shaded with matching colours to predicted lines. In the simulations of oral ziprasidone, 5th percentile included 0 for time points below 6h.

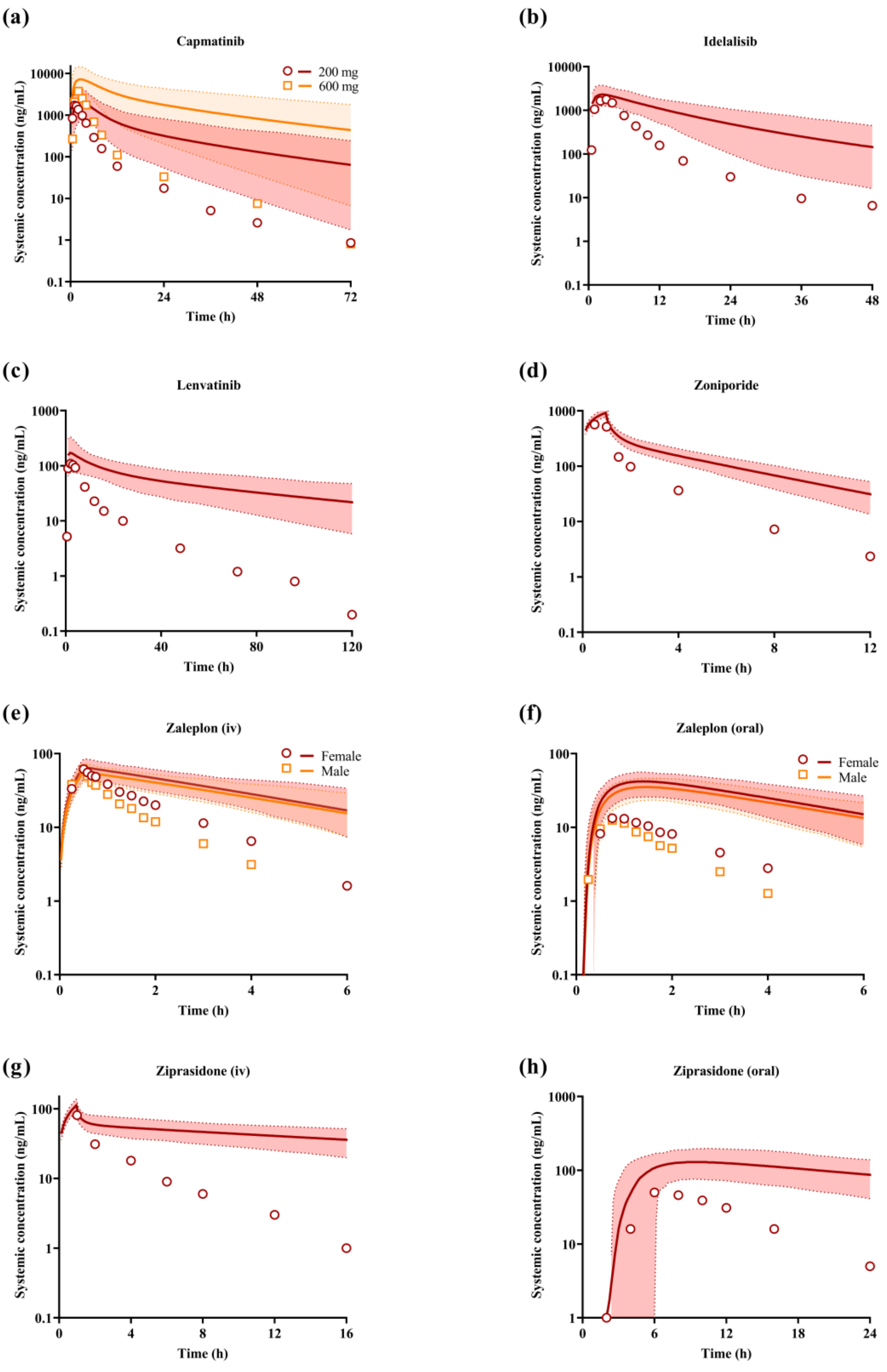

Figure S3. **Simulation of the pharmacokinetics using the bottom-up model from human hepatocytes of a) capmatinib [oral; 200 and 600 mg], b) idelalisib [oral; 150 mg], c) lenvatinib [oral; 10 mg], d) zoniporide [iv infusion; 80 mg], e) zaleplon [iv infusion; 5 mg in female and male], f) zaleplon [oral; 5 mg in female and male], g) ziprasidone [iv infusion; 5 mg], h) ziprasidone [oral; 20 mg]. Solid lines represent the predicted profile. Symbols (circle and square) represent observed data, if available. Area between the dotted lines representing the 5th and 95th percentile of predictions is shaded with matching colours to predicted lines. In the simulations of oral ziprasidone, 5th percentile included 0 for time points below 6h.**

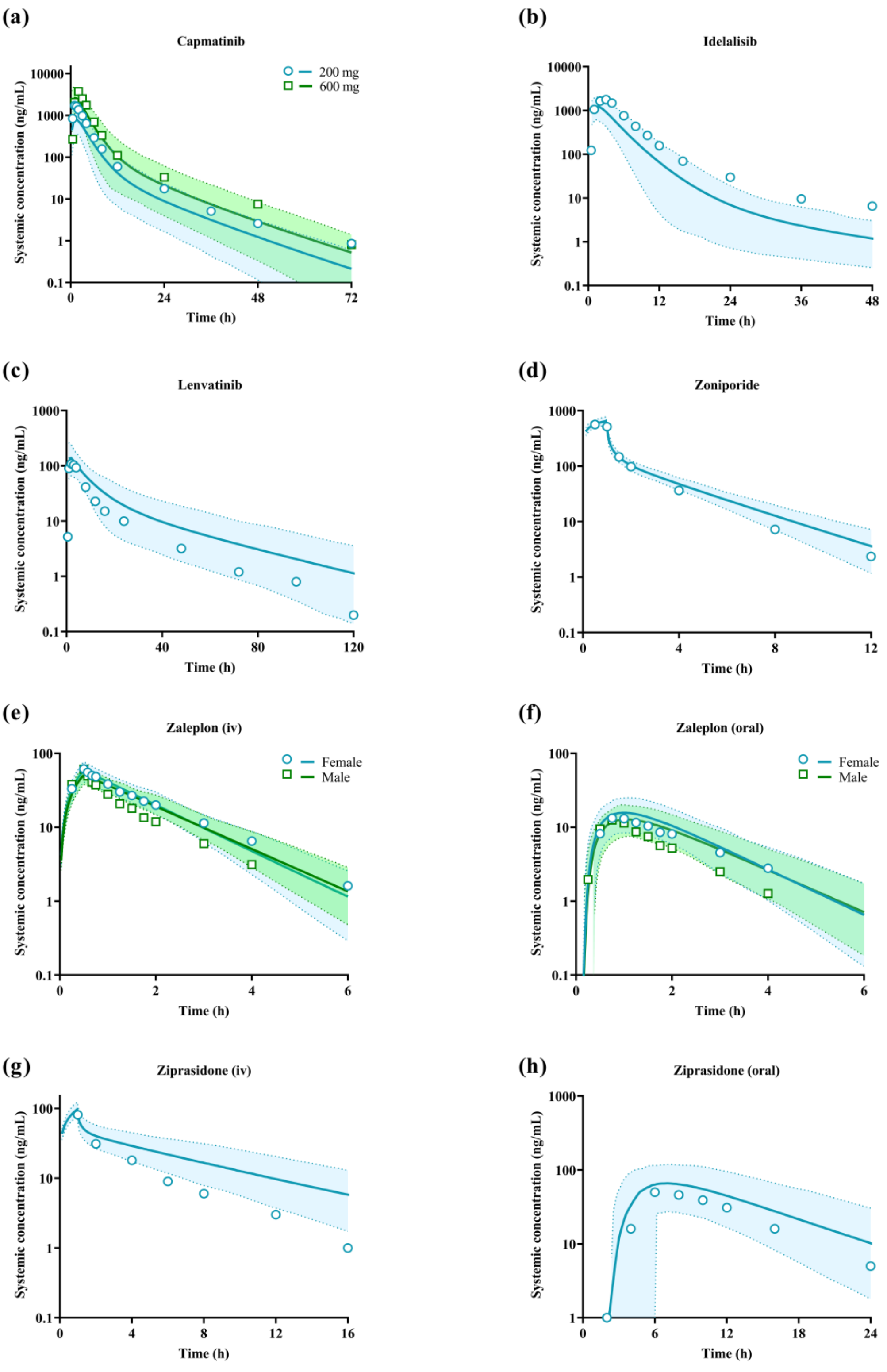

Figure S4. Simulation of the pharmacokinetics using the bottom-up model with empirical scaling factors from human liver microsomes and cytosols of a) capmatinib [oral; 200 and 600 mg], b) idelalisib [oral; 150 mg], c) lenvatinib [oral; 10 mg], d) zoniporide [iv infusion; 80 mg], e) zaleplon [iv infusion; 5 mg in female and male], f) zaleplon [oral; 5 mg in female and male], g) ziprasidone [iv infusion; 5 mg], h) ziprasidone [oral; 20 mg]. Solid lines represent the predicted profile. Symbols (circle and square) represent observed data, if available. Area between the dotted lines representing the 5th and 95th percentile of predictions is shaded with matching colours to predicted lines. In the simulations of oral ziprasidone, 5th percentile included 0 for time points below 6h.

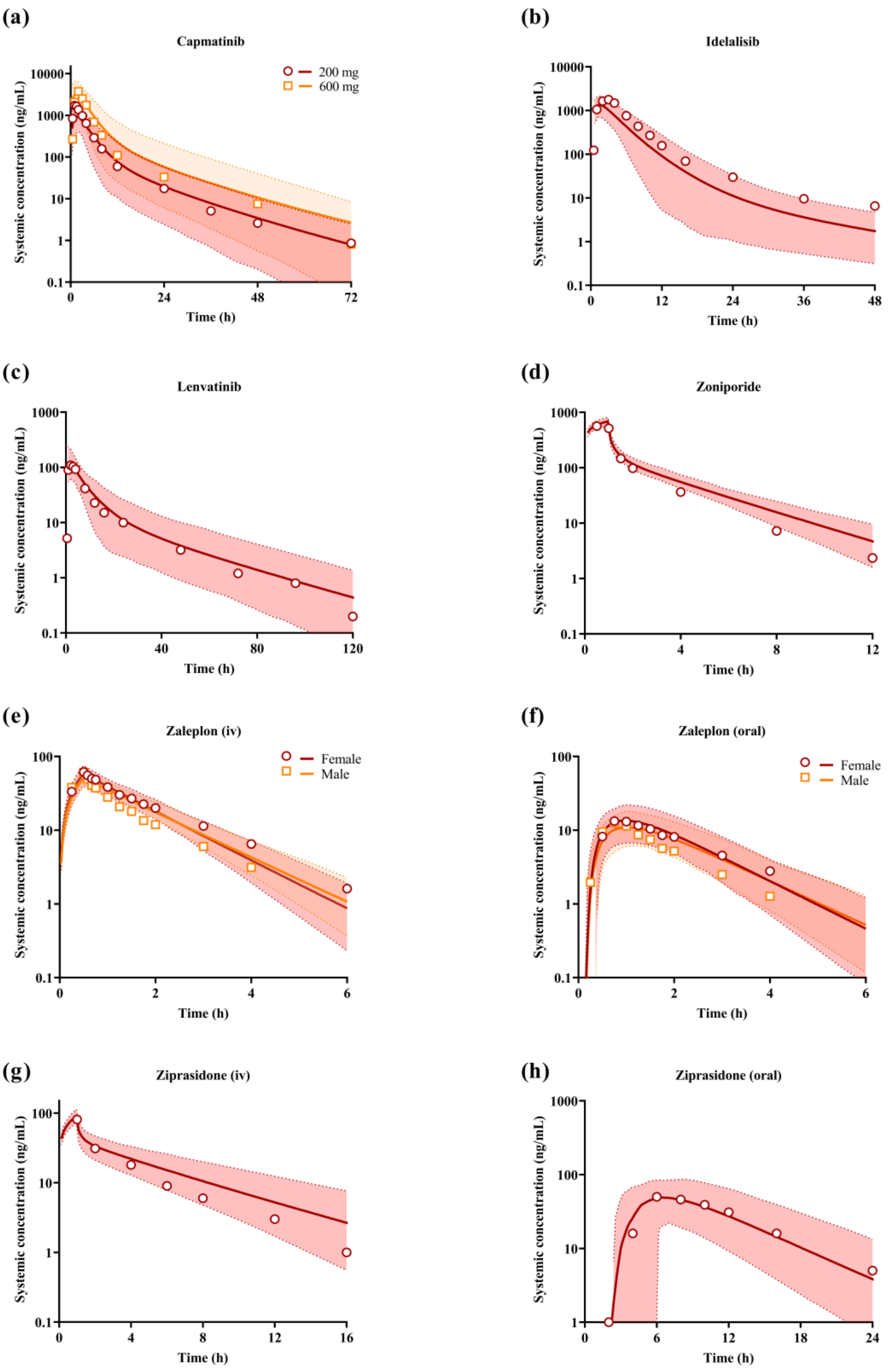

Figure S5. Simulation of the pharmacokinetics using the bottom-up model with empirical scaling factors from human hepatocytes of a) capmatinib [oral; 200 and 600 mg], b) idelalisib [oral; 150 mg], c) lenvatinib [oral; 10 mg], d) zoniporide [iv infusion; 80 mg], e) zaleplon [iv infusion; 5 mg in female and male], f) zaleplon [oral; 5 mg in female and male], g) ziprasidone [iv infusion; 5 mg], h) ziprasidone [oral; 20 mg]. Solid lines represent the predicted profile. Symbols (circle and square) represent observed data, if available. Area between the dotted lines representing the 5th and 95th percentile of predictions is shaded with matching colours to predicted lines. In the simulations of oral ziprasidone, 5th percentile included 0 for time points below 6h.

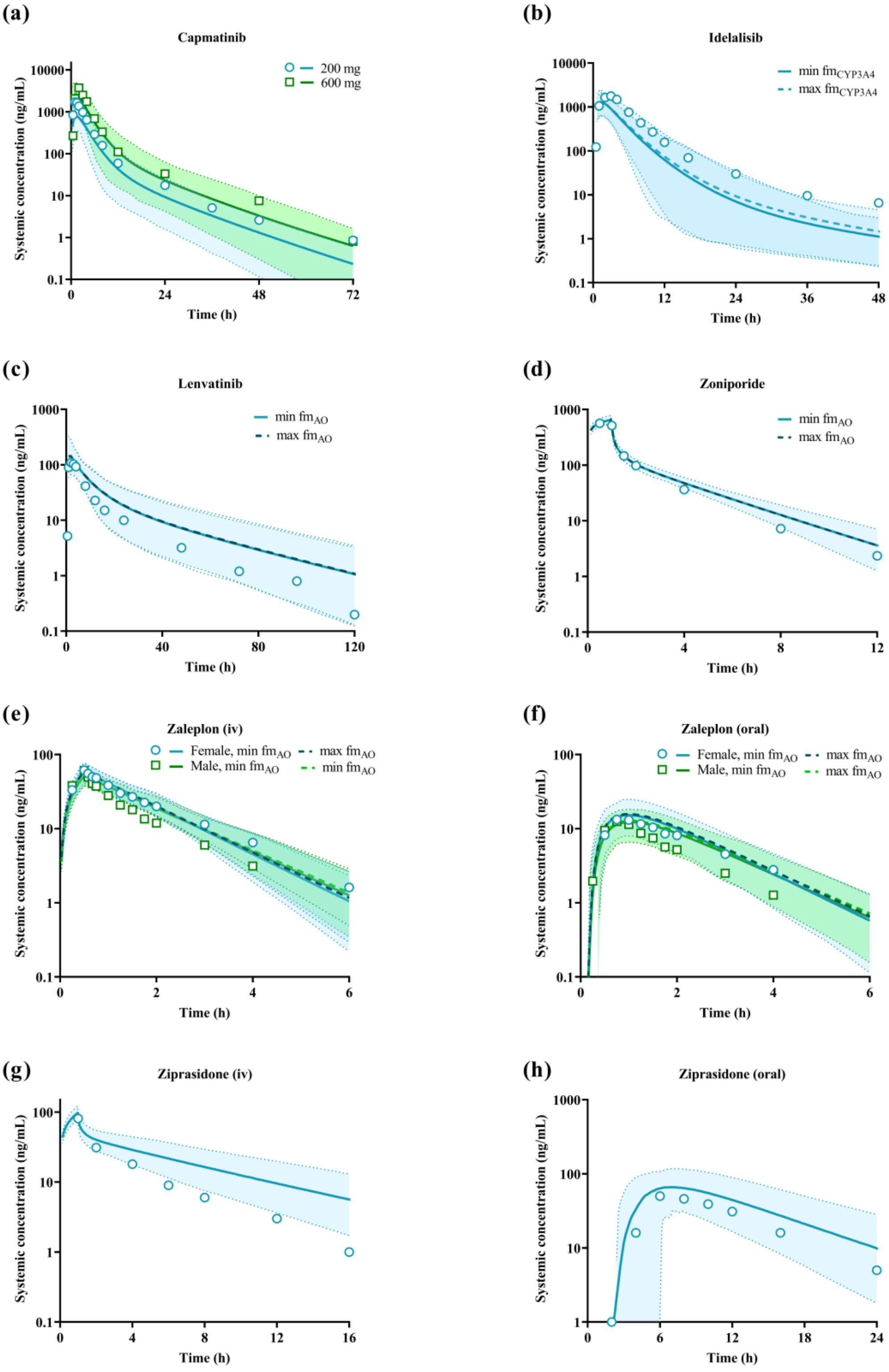

Figure S6. Simulation of the pharmacokinetics using the middle-out model from human liver microsomes and cytosols with observed fraction metabolized data of a) capmatinib [oral; 200 and 600 mg], b) idelalisib [oral; 150 mg], c) lenvatinib [oral; 10 mg], d) zoniporide [iv infusion; 80 mg], e) zaleplon [iv infusion; 5 mg in female and male], f) zaleplon [oral; 5 mg in female and male], g) ziprasidone [iv infusion; 5 mg], h) ziprasidone [oral; 20 mg]. Solid and dashed line represent the predicted profiles with minimum and maximum fm_{AO} /fm_{CYP3A4} assumption, if available. Symbols (circle and square) represent observed data, if available. Area between the dotted lines representing the 5th and 95th percentile of predictions is shaded with matching colours to predicted lines. In the simulations of oral ziprasidone, 5th percentile included 0 for time points below 6h

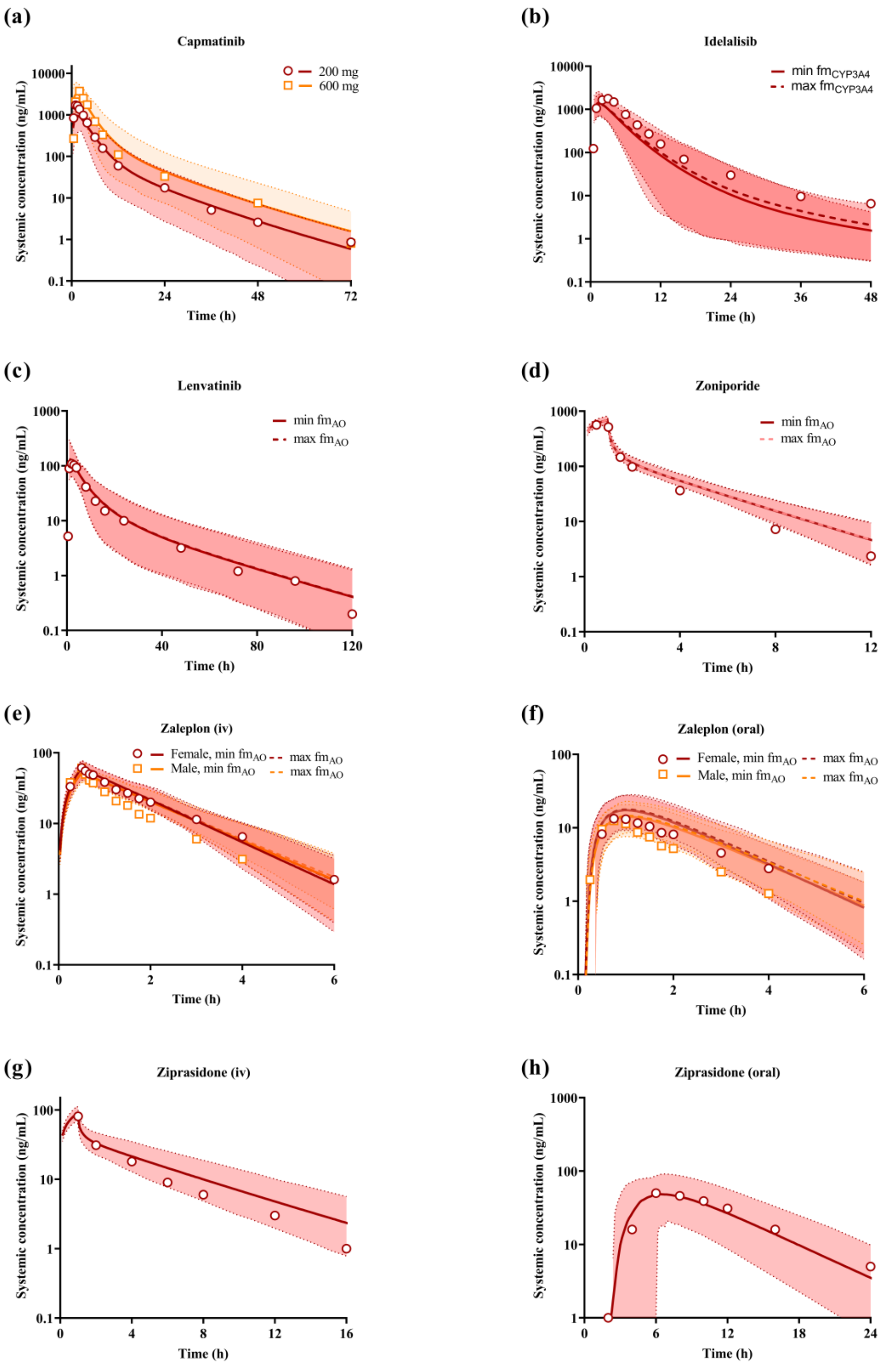

Figure S7. Simulation of the pharmacokinetics using the middle-out model from human hepatocytes with observed fraction metabolized data of a) capmatinib [oral; 200 and 600 mg], b) idelalisib [oral; 150 mg], c) lenvatinib [oral; 10 mg], d) zoniporide [iv infusion; 80 mg], e) zaleplon [iv infusion; 5 mg in female and male], f) zaleplon [oral; 5 mg in female and male], g) ziprasidone [iv infusion; 5 mg], h) ziprasidone [oral; 20 mg]. Solid and dashed line represent the predicted profiles with minimum and maximum fm_{AO} /fm_{CYP3A4} assumption, if available. Symbols (circle and square) represent observed data, if available. Area between the dotted lines representing the 5th and 95th percentile of predictions is shaded with matching colours to predicted lines. In the simulations of oral ziprasidone, 5th percentile included 0 for time points below 6h.

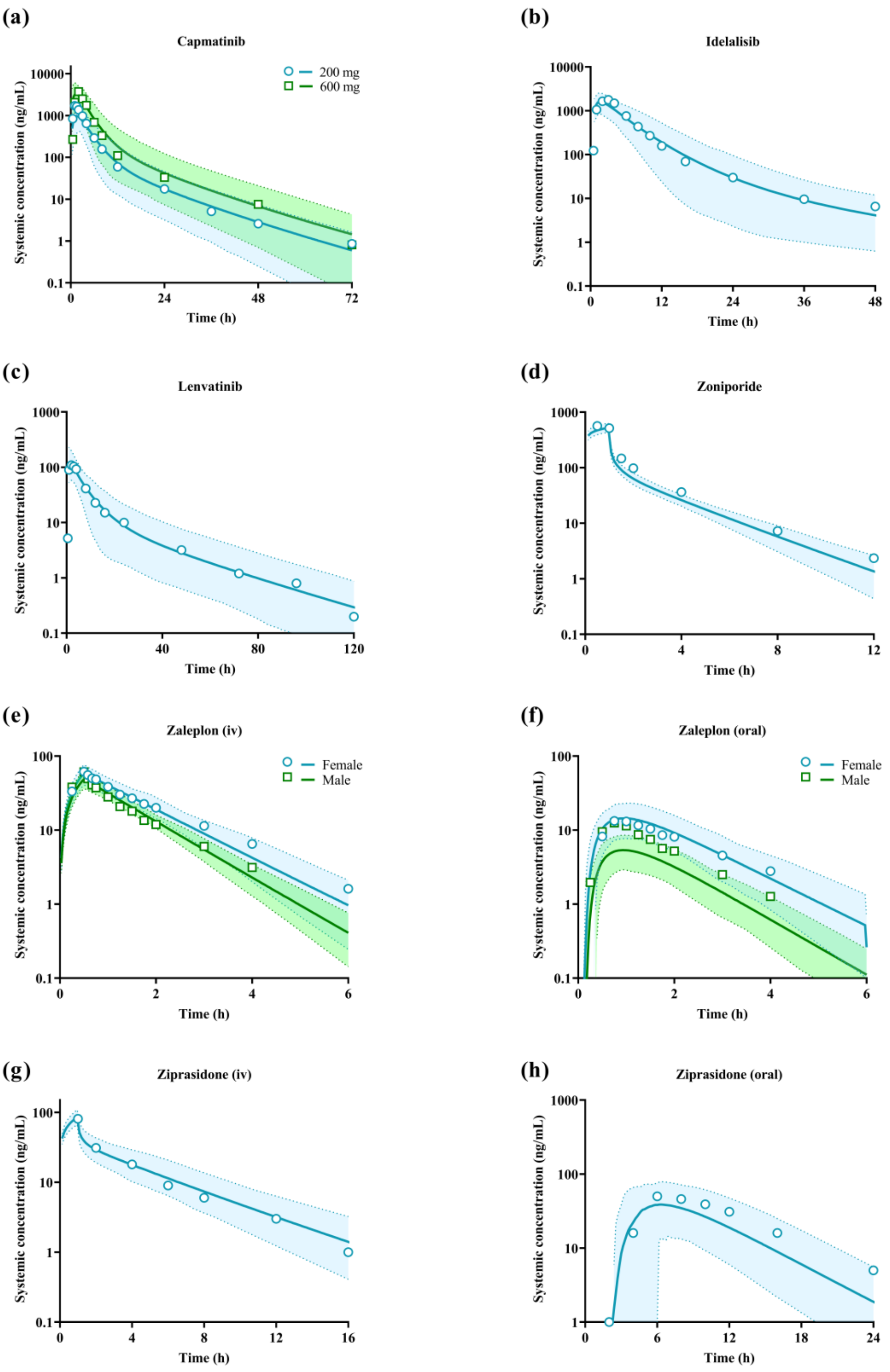

Figure S8. Simulation of the pharmacokinetics using the middle-out model from human liver microsomes and cytosols with observed intrinsic clearance data of a) capmatinib [oral; 200 and 600 mg], b) idelalisib [oral; 150 mg], c) lenvatinib [oral; 10 mg], d) zoniporide [iv infusion; 80 mg], e) zaleplon [iv infusion; 5 mg in female and male], f) zaleplon [oral; 5 mg in female and male], g) ziprasidone [iv infusion; 5 mg], h) ziprasidone [oral; 20 mg]. Solid lines represent the predicted profile. Symbols (circle and square) represent observed data, if available. Area between the dotted lines representing the 5th and 95th percentile of predictions is shaded with matching colours to predicted lines. In the simulations of oral ziprasidone, 5th percentile included 0 for time points below 6h.

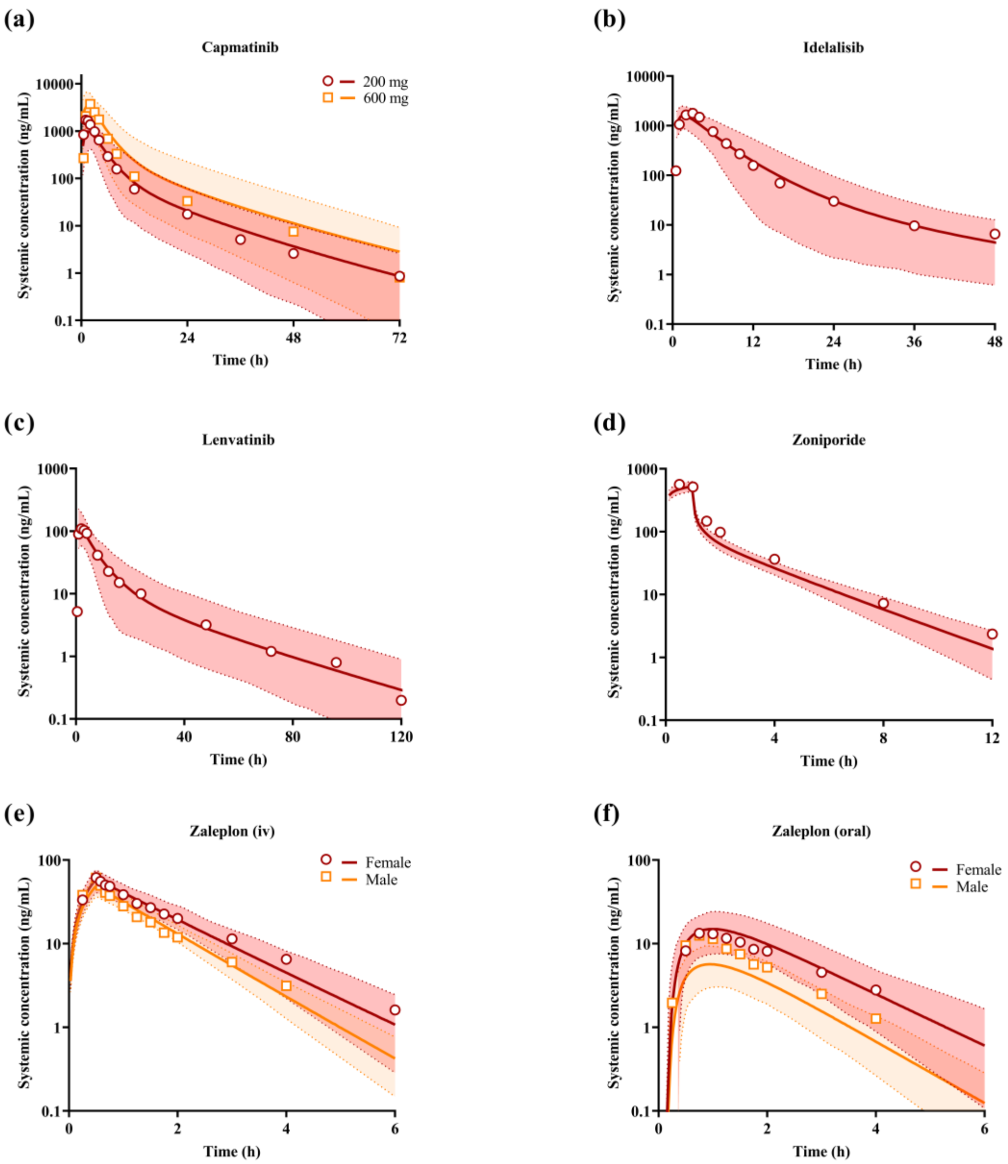

Figure S9. Simulation of the pharmacokinetics using the middle-out model from human hepatocytes with observed intrinsic clearance data of a) capmatinib [oral; 200 and 600 mg], b) idelalisib [oral; 150 mg], c) lenvatinib [oral; 10 mg], d) zoniporide [iv infusion; 80 mg], e) zaleplon [iv infusion; 5 mg in female and male], f) zaleplon [oral; 5 mg in female and male]. Solid lines represent the predicted profile. Symbols (circle and square) represent observed data, if available. Area between the dotted lines representing the 5th and 95th percentile of predictions is shaded with matching colours to predicted lines. In the simulations of oral ziprasidone, 5th percentile included 0 for time points below 6h.

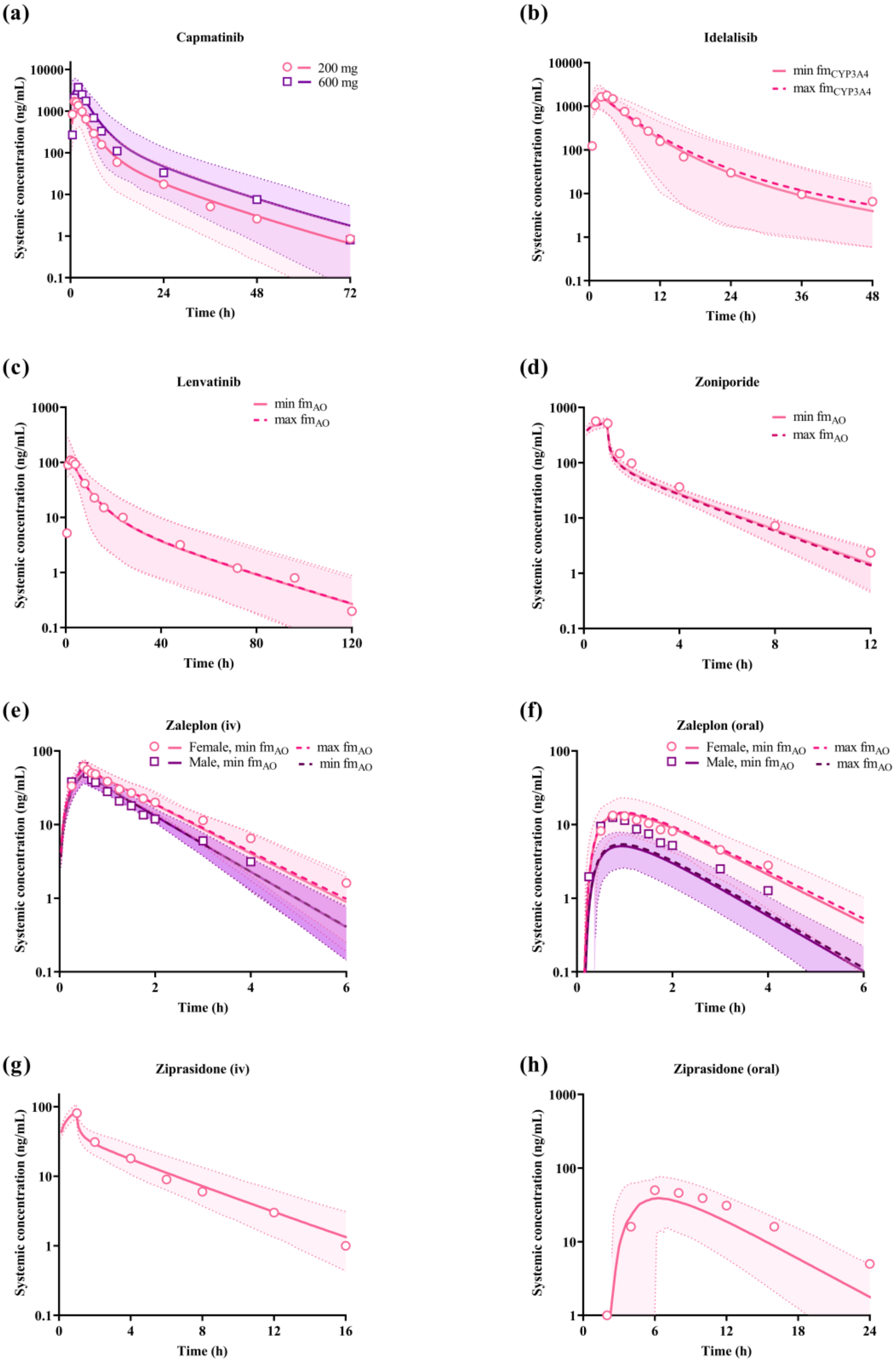

Figure S10. Simulation of the pharmacokinetics using the top-down model with observed fraction metabolized data and observed intrinsic clearance data of a) capmatinib [oral; 200 and 600 mg], b) idelalisib [oral; 150 mg], c) lenvatinib [oral; 10 mg], d) zoniporide [iv infusion; 80 mg], e) zaleplon [iv infusion; 5 mg in female and male], f) zaleplon [oral; 5 mg in female and male], g) ziprasidone [iv infusion; 5 mg], h) ziprasidone [oral; 20 mg]. Solid and dashed line represent the predicted profiles with minimum and maximum fm_{AO} /fm_{CYP3A4} assumption, if available. Symbols (circle and square) represent observed data, if available. Area between the dotted lines representing the 5th and 95th percentile of predictions is shaded with matching colours to predicted lines. In the simulations of oral ziprasidone, 5th percentile included 0 for time points below 6h.

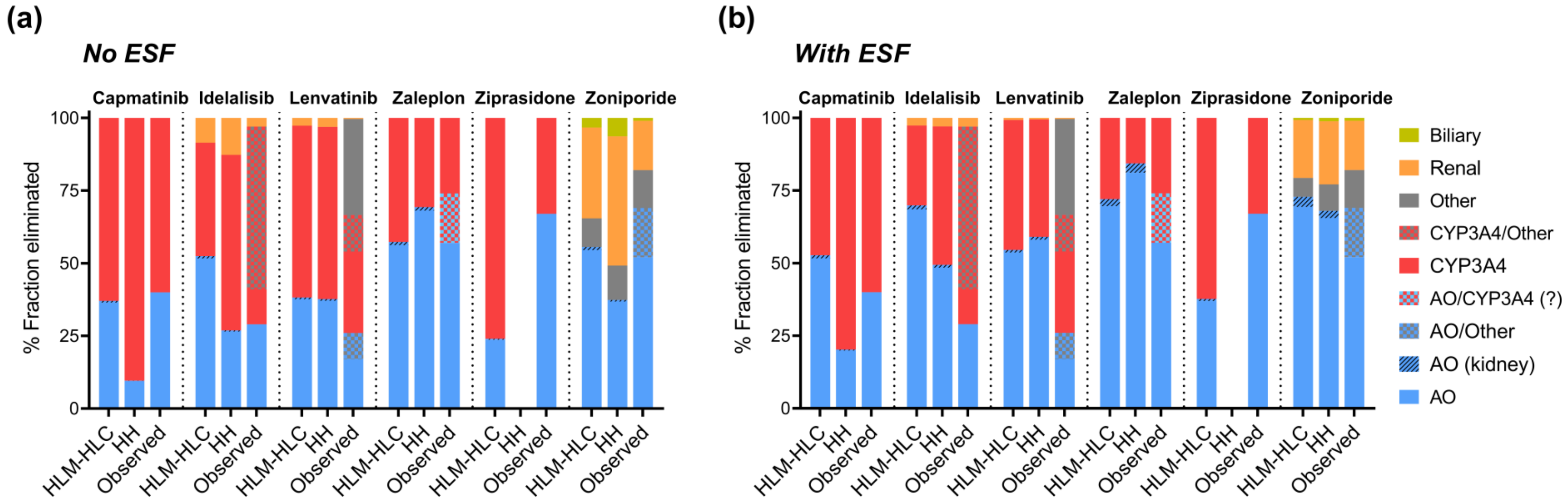

Figure S11. Observed vs. predicted fraction eliminated via bottom-up models (a) in the absence and (b) presence of empirical scaling factors (ESF)[47, 48] using human liver microsomes and cytosols (HLM-HLC) or human hepatocytes (HH) data

Table S10. Summary of observed vs. predicted pharmacokinetic parameters of study compounds with bottom-up PBPK models using the data from human liver microsomes and cytosols and refined models.

PK parameters[a]	Obs.	Bottom-up model (from HLM-HLC)		Bottom-up model with ESF (from HLM-HLC)		Middle-out model with obs fm_{AO} (from HLM-HLC)[b]		Middle-out model with obs $CL_{int,u}$ (from HLM-HLC)		Top-down model with obs fm_{AO} and $CL_{int,u}$[b]	
		Pred.	$R_{pred/obs}$	Pred.	$R_{pred/obs}$	Pred.	$R_{pred/obs}$[c]	Pred.	$R_{pred/obs}$	Pred.	$R_{pred/obs}$[c]
Capmatinib 200 mg-oral											
C_{max} (ng/mL) mean (CV%)	1870 (39.1)	1846 (50.8)	1.0	861 (45.8)	0.5	858 (45.9)	0.5	1065 (46.9)	0.6	1062 (46.9)	0.6
AUC_{inf} (ng.h/mL) mean (CV%)	6970 (47.9)	15783 (48)	2.3	3859 (43.8)	0.6	3883 (45.7)	0.6	5405 (43.9)	0.8	5450 (45.9)	0.8
CL_{oral} (L/h) mean (CV%)	36.1 (52.9)	15.6 (47.4)	0.4	62.2 (45.7)	1.7	62.4 (45.6)	1.7	44.5 (45.9)	1.2	44.5 (45.8)	1.2
Fg (mean)	n/a	1	n/a	0.99	n/a	0.99	n/a	0.99	n/a	0.99	n/a
fm_{AO}	40	36.5 (37.7)	0.9	52.1 (28.9)	1.3	40.7 (35.3)	1.0	52.3 (28.7)	1.3	40.7 (35.3)	1.0
fm_{CYP3A4}	60	63.5 (21.1)	1.1	47.9 (30.2)	0.8	59.3 (23.5)	1.0	47.7 (30.4)	0.8	59.3 (23.5)	1.0
Capmatinib 600 mg-oral											
C_{max} (ng/mL) mean (CV%)	3670 (46.6)	6083 (43.5)	1.7	2828 (43.5)	0.8	2841 (44.4)	0.8	3528 (42.4)	1.0	3546 (43.2)	1.0
AUC_{inf} (ng.h/mL) mean (CV%)	14400 (36.9)	53641 (51.9)	3.7	11361 (43.7)	0.8	11651 (46.5)	0.8	16121 (44.2)	1.1	16647 (47.3)	1.2
CL_{oral} (L/h) mean (CV%)	51.7 (66.5)	14 (47.3)	0.3	62.5 (41.6)	1.2	62.1 (43)	1.2	44.2 (42.1)	0.9	43.7 (43.6)	0.8
Fg (mean)	n/a	1	n/a	0.99	n/a	0.99	n/a	0.99	n/a	0.99	n/a
fm_{AO}	40	37.7 (38.4)	0.9	53.3 (29.6)	1.3	41.9 (36)	1.0	53.5 (29.4)	1.3	41.9 (36)	1.0
fm_{CYP3A4}	60	62.3 (22.7)	1	46.7 (32.6)	0.8	58.1 (25.3)	1.0	46.5 (32.8)	0.8	58.1 (25.3)	1.0

Cont. PK parameters[a]	Obs.	Bottom-up model (from HLM-HLC)		Bottom-up model with ESF (from HLM-HLC)		Middle-out model with obs fm_{AO} (from HLM-HLC)[b]		Middle-out model with obs $CL_{int,u}$ (from HLM-HLC)		Top-down model with obs fm_{AO} and $CL_{int,u}$[b]	
		Pred.	$R_{pred/obs}$	Pred.	$R_{pred/obs}$	Pred.	$R_{pred/obs}$[c]	Pred.	$R_{pred/obs}$	Pred.	$R_{pred/obs}$[c]
Idelalisib 150 mg-oral											
C_{max} (ng/mL) mean (CV%)	2168 (26.4)	2126 (31.3)	1.0	1283 (34)	0.6	1328 (40.5)-1279 (35.3)	0.6	1604 (32.4)	0.7	1670 (40.2)-1601 (33.3)	0.8-0.7
AUC_{inf} (ng.h/mL) mean (CV%)	10508 (28.2)	25625 (39.8)	2.4	6193 (42.3)	0.6	6115 (39.1)-6367 (50.5)	0.6	10321 (41.5)	1.0	10198 (38.6)-10630 (49.4)	1
CL_{oral} (L/h) mean (CV%)	15.3 (28.2)	6.78 (40.6)	0.4	28.6 (43.4)	1.9	28.8 (43.7)-29.3 (48.8)	1.9	17.1 (42.7)	1.1	17.2 (43.1)-17.4 (48.2)	1.1
Fg (mean)	n/a	1	n/a	1	n/a	1-0.99	n/a	1	n/a	1-0.99	n/a
fm_{AO}	29	52.4 (27.4)	1.8	69.9 (19.6)	2.4	29.5 (35.7)-31.1 (42.2)	1.0-1.1	67.7 (20.2)	2.3	29.1 (35.6)-30.6 (42)	1.0-1.1
fm_{CYP3A4} (min-max)	12-68	39.0 (36.4)	1.0	27.5 (46.4)	0.7	12.4 (47.4)-66.2 (20)	0.3-1.7	28.4 (45.4)	0.7	12.3 (47.4)-65.4 (20.3)	0.3-1.6
Lenvatinib 10 mg-oral											
C_{max} (ng/mL) geomean (CV%)	100 (64.1)	154 (48.2)	1.5	131 (46.1)	1.3	132 (53.9)-132 (53.8)	1.3	117 (45.2)	1.2	117 (52.5)-117 (52.3)	1.2
AUC_{inf} (ng.h/mL) geomean (CV%)	1080 (40.4)	6905 (41.3)	6.4	1784 (39.4)	1.7	1735 (38.4)-1746 (38.3)	1.6	1058 (39.8)	1.0	1029 (38.7)-1035 (38.7)	1.0
CL_{oral} (L/h) mean (CV%)	9.9 (34.3)	1.56 (40.8)	0.2	6.02 (39)	0.6	6.17 (38.6)-6.13 (37.9)	0.6	10.16 (39.6)	1.0	10.42 (38.9)-10.35 (38.2)	1.1-1.0
Fg (mean)	n/a	1	n/a	1	n/a	1	n/a	1	n/a	1	n/a
fm_{AO} (min-max)	17-26	38.2 (36.5)	1.8	54.5 (27.9)	2.5	17.6 (41)-25.7 (37.9)	0.8-1.2	54.7 (27.9)	2.5	17.7 (41)-25.8 (37.9)	0.8-1.2
fm_{CYP3A4} (min-max)	28-41	59.1 (23.7)	1.7	44.7 (33)	1.3	27.1 (35.2)-39.2 (29.1)	1.3-1.1	44.8 (32.9)	1.3	27.1 (35.2)-39.3 (29.1)	1.3-1.1

Cont. PK parameters[a]		Obs.	Bottom-up model (from HLM-HLC)		Bottom-up model with ESF (from HLM-HLC)		Middle-out model with obs fm_{AO} (from HLM-HLC)[b]		Middle-out model with obs $CL_{int,u}$ (from HLM-HLC)		Top-down model with obs fm_{AO} and $CL_{int,u}$[b]	
			**Pred.**	**$R_{pred/obs}$**	**Pred.**	**$R_{pred/obs}$**	**Pred.**	**$R_{pred/obs}$**[c]	**Pred.**	**$R_{pred/obs}$**	**Pred.**	**$R_{pred/obs}$**[c]
Zaleplon 5 mg - iv												
**C_{max} (ng/mL)**	F	62.7 (23.8)	64.1 (20.1)	1.0	58.6 (19.1)	0.9	58.5 (19.2)-58.6 (19.1)	0.9	58.1 (19)	0.9	58.1 (19.1)-58.1 (19)	0.9
**mean (CV%)**	M	61.4 (26.4)	55.3 (17.2)	0.9	51 (16.4)	0.8	51 (16.5)-51.1 (16.4)	0.8	48.5 (16)	0.8	48.5 (16.1)-48.4 (16)	0.8
**AUC_{inf} (ng.h/mL)**	F	100 (24)	209 (31.3)	2.1	97.8 (18.3)	1.0	96.4 (19.1)-97.9 (18.2)	1.0	93.7 (17.6)	0.9	92.6 (18.3)-93.9 (17.5)	0.9
**mean (CV%)**	M	71.7 (12)	188 (29.7)	2.6	91.6 (16.8)	1.3	90.3 (17.6)-91.7 (16.8)	1.3	68.8 (12.7)	1.0	68.9 (13)-68.8 (12.7)	1.0
**CL_{iv} (L/h) mean**	F	52.5 (22.5)	26.0 (28)	0.5	52.7 (16.9)	1.0	53.6 (17.6)-52.6 (16.8)	1.0	54.9 (16.3)	1.0	55.7 (17)-54.8 (16.3)	1.1-1.0
**(CV%)**	M	71.6 (20.2)	28.6 (26.7)	0.4	56 (15.7)	0.8	56.9 (16.4)-56 (15.6)	0.8	73.8 (12.5)	1.0	73.7 (12.8)-73.8 (12.5)	1.0
**fm_{AO} (min-max)**	F	57-74	57.4 (26.1)	0.9	72.1 (18.9)	1.1	57.4 (26.7)-73.1 (18.3)	0.9-1.1	71.4 (19.4)	1.1	57.4 (26.7)-73.2 (18.4)	0.9-1.1
	M		57.2 (26.4)		72 (19.1)		57.3 (27)-73 (18.5)		71.7 (20.6)		58.1 (27.9)-73.6 (19.6)	
**fm_{CYP3A4} (min-max)**	F	26-43	42.6 (33.6)	1.2	27.9 (43.9)	0.8	42.6 (33.5)-26.9 (44.7)	1.2-0.8	28.6 (43.3)	0.8	42.6 (33.5)-26.8 (44.7)	1.2-0.8
	M		42.8 (33.7)		28 (43.8)		42.7 (33.6)-27 (44.6)		28.3 (43.4)		41.9 (33.7)-26.4 (44.9)	
Zaleplon 5 mg – oral												
**C_{max} (ng/mL)**	F	14.4 (38.9)	35.9 (24.8)	2.5	15.9 (31.7)	1.1	15.4 (32.8)-15.9 (31.7)	1.1	14.6 (32.4)	1.0	14.1 (33.5)-14.6 (32.3)	1.0
**mean (CV%)**	M	14.5 (45.5)	29.8 (22.8)	2.1	12.8 (30.1)	0.9	12.4 (31.2)-12.9 (30.1)	0.9	5.4 (35.1)	0.4	5.1 (36.5)-5.5 (35)	0.4
**AUC_{inf} (ng.h/mL)**	F	31.4 (57.3)	147 (39.5)	4.7	37.6 (37.7)	1.2	35.8 (39.5)-37.8 (37.7)	1.1	33.6 (37.8)	1.1	32 (39.6)-33.8 (37.7)	1.0-1.1
**mean (CV%)**	M	22.5 (45.8)	128 (38.6)	5.7	32.8 (37)	1.5	31.2 (38.8)-33 (36.9)	1.4-1.5	11.3 (37.8)	0.5	10.7 (40)-11.5 (37.6)	0.5
**CL_{oral} (L/h)**	F	159.2 (57.3)	38.8 (37.4)	0.2	150.8 (37.1)	0.9	159.9 (37.6)-150.1 (37.2)	1.0-0.9	169.1 (37.1)	1.1	178.8 (37.7)-167.9 (37.3)	1.1
**mean (CV%)**	M	222.2 (45.8)	44.3 (37.2)	0.2	172.4 (36.9)	0.8	183.1 (37.5)-171.7 (37)	0.8	501.3 (37.7)	2.3	536 (38.3)-496.5 (37.9)	2.4-2.2
**Fg**	F	n/a	0.99	n/a	0.98	n/a	0.97-0.98	n/a	0.98	n/a	0.97-0.98	n/a
**(mean)**	M	n/a	0.99	n/a	0.98	n/a	0.97-0.98	n/a	0.95	n/a	0.93-0.95	n/a

Cont. PK parameters[a]	Obs.	Bottom-up model (from HLM-HLC)		Bottom-up model with ESF (from HLM-HLC)		Middle-out model with obs fm_{AO} (from HLM-HLC)[b]		Middle-out model with obs $CL_{int,u}$ (from HLM-HLC)		Top-down model with obs fm_{AO} and $CL_{int,u}$[b]	
		**Pred.**	**$R_{pred/obs}$**	**Pred.**	**$R_{pred/obs}$**	**Pred.**	**$R_{pred/obs}$[c]**	**Pred.**	**$R_{pred/obs}$**	**Pred.**	**$R_{pred/obs}$[c]**
Ziprasidone 5 mg-iv											
**C_{max} (ng/mL) geomean (CV%)**	83 (21)	110 (13.4)	1.3	97.6 (14.3)	1.2	97.5 (14)	1.2	85.7 (14.7)	1.0	85.6 (14.4)	1.0
**AUC_{inf} (ng.h/mL) geomean (CV%)**	217 (20)	1085 (44.5)	5.0	384 (34)	1.8	381 (31.8)	1.8	221 (26.5)	1.0	220 (25)	1.0
**CL_{iv} (L/h) mean (CV%)**	23.04 (14)	5.03 (42.3)	0.2	13.7 (31.8)	0.6	13.8 (30.1)	0.6	23.3 (24.8)	1.0	23.4 (23.5)	1.0
**fm_{AO}**	67	24.0 (44.6)	0.4	37.6 (36.7)	0.6	66.3 (21)	1.0	39.4 (36)	0.6	66.5 (21.3)	1.0
**fm_{CYP3A4}**	33	76.0 (13.8)	2.3	62.4 (21.5)	1.9	33.7 (39)	1.0	60.6 (22.4)	1.8	33.5 (39.1)	1.0
Ziprasidone 20 mg-oral											
**C_{max} (ng/mL) geomean (CV%)**	64 (28)	108.9 (33.1)	1.7	66.5 (38)	1.0	66.8 (36.6)	1.0	40.0 (42.8)	0.6	40.5 (41.2)	0.6
**AUC_{inf} (ng.h/mL) geomean (CV%)**	514 (27)	2616 (57.4)	5.1	752 (53.5)	1.5	753 (50.3)	1.5	322 (53.1)	0.6	325 (50.4)	0.6
**CL_{oral} (L/h) mean (CV%)**	38.9 (27)	8.80 (57.8)	0.2	30.2 (54.7)	0.8	29.8 (52.9)	0.8	70.3 (54.4)	1.8	69.1 (53.0)	1.8
**Fg (mean)**	n/a	1	n/a	0.99	n/a	0.99	n/a	0.98	n/a	0.99	n/a
Zoniporide 80 mg-iv											
**C_{max} (ng/mL) mean (CV%)**	590 (7.49)	822 (11.5)	1.4	657 (11.4)	1.1	658 (11.2)-657 (11.3)	1.1	527 (12.2)	0.9	538 (12.1)-529 (12.2)	0.9
**AUC_{inf} (ng.h/mL) mean (CV%)**	839 (4.08)	1557 (17.2)	1.9	984 (12.5)	1.2	984 (11.8)-983 (12.2)	1.2	697 (12.7)	0.8	719 (12.6)-702 (12.7)	0.9-0.8
**CL_{iv} (L/h) mean (CV%)**	95.4 (5.71)	52.8 (16.3)	0.6	82.5 (12.1)	0.9	82.4 (11.4)-82.5 (11.8)	0.9	117(12.6)	1.2	113.1 (12.6)-115.8 (12.6)	1.2
**fm_{AO}**	52-69	55.6 (15.9)	0.9	72.8 (9.4)	1.2	49.8 (23)-65.8 (13.3)	0.8-1.1	79.3 (11.4)	1.3	58.5 (23.3)-73.5 (14.4)	1.0-1.2
**fm_{other}**	13-30	9.82 (42.1)	0.5	6.50 (49.3)	0.3	29.48 (33.1)-13.5 (44)	1.4-0.6	6.45 (51.3)	0.3	26.83 (34.5)-12.15 (46.1)	1.2-1.4

Abbreviations: AUC_{inf}, area under curve extrapolated to infinity, $CL_{int,u}$, hepatic unbound intrinsic clearance, CL_{iv}, total plasma clearance after intraveneous administration, CL_{oral}, total plasma clearance after oral administration CI, confidence interval, C_{max}, maximum systemic concentration, CV, coefficient of variation, F, female, fm_{AO}, fraction metabolized by aldehyde oxidase, HLC, human liver cytosols, HLM, human liver microsomes, M, male, n/a, not available, Obs, observed, Pred, predicted, $R_{pred/obs}$, ratio of predicted to observed

[a]Pharmacokinetic parameters were represented as mean or geometric mean with CV% or geometric CV%, respectively.

[b]Two predicted values (separated with dash) were given when simulations were performed with minimum and maximum fm_{AO} assumptions.

[c]For $R_{pred/obs}$ of fm_i, mean of predicted values were considered when simulations were performed with minimum and maximum $fm_{i.}$

Table S11. Summary of observed vs. predicted pharmacokinetic parameters of study compounds with bottom-up PBPK models using the data from human hepatocytes (with and without hydralazine) and refined models.

PK parameters[a]	Obs.	Bottom-up model (from HH)		Bottom-up model with ESF (from HH)		Middle-out model with obs fm_{AO} (from HH) [b]		Middle-out model with obs $CL_{int,u}$ (from HH)		Top-down model with obs fm_{AO} and $CL_{int,u}$ [b]	
		Pred.	$R_{pred/obs}$	Pred.	$R_{pred/obs}$	Pred.	$R_{pred/obs}$[c]	Pred.	$R_{pred/obs}$	Pred.	$R_{pred/obs}$[c]
Capmatinib 200 mg-oral											
C_{max} (ng/mL) mean (CV%)	1870 (39.1)	2294 (52.6)	1.2	1044 (48.2)	0.6	1037 (46.8)	0.6	1067 (48)	0.6	1062 (46.9)	0.6
AUC_{inf} (ng.h/mL) mean (CV%)	6970 (47.9)	31030 (61.6)	4.4	5463 (51.8)	0.8	5243 (45.8)	0.8	5648 (51.1)	0.8	5450 (45.9)	0.8
CL_{oral} (L/h) mean (CV%)	36.1 (52.9)	8.85 (57.1)	0.2	46.4 (49.7)	1.3	46.3 (45.8)	1.3	44.6 (49.2)	1.2	44.5 (45.8)	1.2
Fg (mean)	n/a	1	n/a	0.98	n/a	0.99	n/a	0.99	n/a	0.99	n/a
fm_{AO}	40	9.36 (56.1)	0.2	19.8 (48.4)	0.5	40.7 (35.3)	1.0	21.9 (47)	0.5	40.7 (35.3)	1.0
fm_{CYP3A4}	60	90.6 (5.72)	1.5	80.2 (11.7)	1.3	59.3 (23.5)	1.0	78.1 (12.9)	1.3	59.3 (23.5)	1.0
Capmatinib 600 mg-oral											
C_{max} (ng/mL) mean (CV%)	3670 (46.6)	7577 (47.4)	2.1	3541 (45.8)	1.0	3463 (43.3)	0.9	3616 (45.4)	1.0	3546 (43.2)	1.9
AUC_{inf} (ng.h/mL) mean (CV%)	14400 (36.9)	154197 (82)	10.7	17635 (56.2)	1.2	15980 (47.2)	1.1	18172 (55.2)	1.3	16647 (47.3)	2.4
CL_{oral} (L/h) mean (CV%)	51.7 (66.5)	6.4 (69.6)	0.1	44.6 (51.5)	0.9	45.5 (43.5)	0.9	42.9 (50.6)	0.8	43.7 (43.6)	1.2
Fg (mean)	n/a	1	n/a	0.99	n/a	0.99	n/a	0.99	n/a	0.99	n/a
fm_{AO}	40	9.90 (56.7)	0.2	20.7 (49.1)	0.5	41.9 (36)	1.1	22.9 (47.6)	0.6	41.9 (36)	1.0
fm_{CYP3A4}	60	90.1 (6.16)	1.5	79.3 (12.6)	1.3	58.1 (25.3)	1.0	77.1 (13.9)	1.3	58.1 (25.3)	1.0

Cont. PK parameters[a]	Obs.	Bottom-up model (from HLM-HLC)		Bottom-up model with ESF (from HLM-HLC)		Middle-out model with obs fm_{AO} (from HLM-HLC)[b]		Middle-out model with obs $CL_{int,u}$ (from HLM-HLC)		Top-down model with obs fm_{AO} and $CL_{int,u}$[b]	
		Pred.	$R_{pred/obs}$	Pred.	$R_{pred/obs}$	Pred.	$R_{pred/obs}$[c]	Pred.	$R_{pred/obs}$	Pred.	$R_{pred/obs}$[c]
Idelalisib 150 mg-oral											
**C_{max} (ng/mL) mean (CV%)**	2168 (26.4)	2316 (31.5)	1.1	1365 (33.5)	0.6	1423 (40.4)-1369 (34.7)	0.7-0.6	1598 (32.4)	0.7	1670 (40.2)-1601 (33.3)	0.8-0.7
**AUC_{inf} (ng.h/mL) mean (CV%)**	10508 (28.2)	39465 (43.6)	3.8	7128 (44.7)	0.7	7051 (39)-7346 (50.2)	0.7	10314 (43.9)	1.0	10198 (38.6)-10630 (49.4)	1
**CL_{oral} (L/h) mean (CV%)**	15.3 (28.2)	4.54 (44.7)	0.3	25.08 (43.8)	1.6	24.95 (43.6)-25.37 (48.7)	1.6-1.7	17.25 (43.3)	1.1	17.2 (43.1)-17.4 (48.2)	1.1
**Fg (mean)**	n/a	1	n/a	0.99	n/a	1-0.99	n/a	1	n/a	1-0.99	n/a
**fm_{AO}**	29	26.9 (41.3)	0.9	49.4 (31.1)	1.7	29.4 (35.7)-31 (42.2)	1.0-1.1	49.3 (30.8)	1.7	29.1 (35.6)-30.6 (42)	1.0-1.1
**fm_{CYP3A4} (min-max)**	12-68	60.4 (22.3)	1.5	47.7 (31.6)	1.2	12.4 (47.4)-66 (20.1)	0.3-1.7	46.8 (32)	1.2	12.3 (47.4)-65.4 (20.3)	0.3-1.6
Lenvatinib 10 mg-oral											
**C_{max} (ng/mL) geomean (CV%)**	100 (64.1)	156 (48.3)	1.6	122 (45.5)	1.2	122 (52.9)-122 (52.8)	1.2	117 (45.3)	1.2	117 (52.5)-117 (52.3)	1.2
**AUC_{inf} (ng.h/mL) geomean (CV%)**	1080 (40.4)	8124 (41.1)	7.5	1240 (39.2)	1.1	1205 (38.6)-1213 (38.5)	1.1	1058 (39.4)	1.0	1029 (38.7)-1035 (38.7)	1.0
**CL_{oral} (L/h) mean (CV%)**	9.9 (34.3)	1.33 (40.7)	0.1	8.66 (39.3)	0.9	8.89 (38.8)-8.83 (38.1)	0.9	10.15 (39.5)	1.0	10.42 (38.9)-10.35 (38.2)	1.1-1.0
**Fg (mean)**	n/a	1	n/a	1	n/a	1	n/a	1	n/a	1	n/a
**fm_{AO} (min-max)**	17-26	37.6 (36.7)	1.8	59 (25.5)	2.7	17.7 (41)-25.8 (37.9)	0.8-1.2	58.7 (25.7)	2.7	17.7 (41)-25.8 (37.9)	0.8-1.2
**fm_{CYP3A4} (min-max)**	28-41	59.2 (23.6)	1.7	40.4 (35.9)	1.2	27.1 (35.2)-39.3 (29.1)	0.8-1.1	40.8 (35.6)	1.2	27.1 (35.2)-39.3 (29.1)	1.3-1.1

Cont. PK parameters[a]		Obs.	Bottom-up model (from HLM-HLC)		Bottom-up model with ESF (from HLM-HLC)		Middle-out model with obs fm_{AO} (from HLM-HLC)[b]		Middle-out model with obs $CL_{int,u}$ (from HLM-HLC)		Top-down model with obs fm_{AO} and $CL_{int,u}$[b]	
			Pred.	$R_{pred/obs}$	Pred.	$R_{pred/obs}$	Pred.	$R_{pred/obs}$[c]	Pred.	$R_{pred/obs}$	Pred.	$R_{pred/obs}$[c]
Zaleplon 5 mg - iv												
C_{max} (ng/mL) mean (CV%)	F	62.7 (23.8)	65.3 (20.2)	1.0	57.7 (18.8)	0.9	59.2 (19.3)-59.3 (19.2)	0.9	58.2 (18.9)	0.9	58.1 (19.1)-58.1 (19)	0.9
	M	61.4 (26.4)	56.2 (17.2)	0.9	50.4 (16.2)	0.8	51.5 (16.6)-51.6 (16.5)	0.8	48.4 (16)	0.8	48.5 (16.1)-48.4 (16)	0.8
AUC_{inf} (ng.h/mL) mean (CV%)	F	100 (24)	291 (33.3)	2.9	90.5 (16.5)	0.9	103.3 (20.4)-105.3 (19.5)	1.0-1.1	95.2 (17.3)	1.0	92.6 (18.3)-93.9 (17.5)	0.9
	M	71.7 (12)	260 (31.8)	3.6	85.3 (15.2)	1.2	96.3 (18.8)-98.1 (18)	1.3-1.4	69 (12.6)	1.0	68.9 (13)-68.8 (12.7)	1.0
CL_{iv} (L/h) mean (CV%)	F	52.5 (22.5)	18.9 (29.6)	0.4	56.6 (15.5)	1.1	50.2 (18.6)-49.1 (17.8)	1.0-0.9	54 (16.1)	1.0	55.7 (17)-54.8 (16.3)	1.1-1.0
	M	71.6 (20.2)	20.9 (28.4)	0.3	59.9 (14.4)	0.8	53.6 (17.4)-52.4 (16.5)	0.7	73.6 (12.4)	1.0	73.7 (12.8)-73.8 (12.5)	1.0
fm_{AO} (min-max)	F	57-74	69.4 (19.6)	1.1	84.3 (12.3)	1.3	57.3 (26.6)-73.1 (18.2)	0.9-1.1	83.2 (12.8)	1.3	57.4 (26.7)-73.2 (18.4)	0.9-1.1
	M		69.2 (19.8)		84.3 (12.5)		57.2 (26.9)-72.9 (18.4)		83.4 (14.2)		58.1 (27.9)-73.6 (19.6)	
fm_{CYP3A4} (min-max)	F	26-43	30.6 (42.0)	0.9	15.7 (54.4)	0.5	42.7 (33.5)-26.9 (44.7)	1.2-0.8	16.8 (53.3)	0.5	42.6 (33.5)-26.8 (44.7)	1.2-0.8
	M		30.8 (41.9)		15.7 (54.2)		42.8 (33.5)-27.1 (44.6)		16.6 (53.4)		41.9 (33.7)-26.4 (44.9)	
Zaleplon 5 mg – oral												
C_{max} (ng/mL) mean (CV%)	F	14.4 (38.9)	42.2 (22.8)	2.9	13.6 (33.3)	0.9	17.4 (31.8)-18 (30.7)	1.2-1.3	15.1 (32.4)	1.0	14.1 (33.5)-14.6 (32.3)	1.0
	M	14.5 (45.5)	35.3 (20.5)	2.4	11.02 (31.9)	0.8	14.1 (30.1)-14.6 (29.1)	1.0	5.7 (35.3)	0.4	5.1 (36.5)-5.5 (35)	0.4
AUC_{inf} (ng.h/mL) mean (CV%)	F	31.4 (57.3)	228 (38.5)	7.2	31 (37.8)	1.0	42.6 (39.4)-45.1 (37.6)	1.4	35.5 (37.7)	1.1	32 (39.6)-33.8 (37.7)	1.0-1.1
	M	22.5 (45.8)	199 (37.6)	8.8	27.1 (37.1)	1.2	37.1 (38.6)-39.3 (36.8)	1.6-1.7	12.1 (37.5)	0.5	10.7 (40)-11.5 (37.6)	0.5
CL_{oral} (L/h) mean (CV%)	F	159.2 (57.3)	24.9 (36.5)	0.2	184.8 (39.6)	1.2	134.2 (37.5)-125.9 (37)	0.8	160.9 (39.2)	1.0	178.8 (37.7)-167.9 (37.3)	1.1
	M	222.2 (45.8)	28.4 (36.2)	0.1	210.7 (39.4)	0.9	153.6 (37.4)-143.9 (36.8)	0.7-0.6	472.6 (39.7)	2.1	536 (38.3)-496.5 (37.9)	2.4-2.2
Fg (mean)	F	n/a	1	n/a	0.99	n/a	0.98-0.99	n/a	0.99	n/a	0.97-0.98	n/a
	M	n/a	1	n/a	0.99	n/a	0.98-0.99	n/a	0.97	n/a	0.93-0.95	n/a

Cont. PK parameters[a]	Obs.	Bottom-up model (from HLM-HLC)		Bottom-up model with ESF (from HLM-HLC)		Middle-out model with obs fm_{AO} (from HLM-HLC)[b]		Middle-out model with obs $CL_{int,u}$ (from HLM-HLC)		Top-down model with obs fm_{AO} and $CL_{int,u}$[b]	
		Pred.	$R_{pred/obs}$	Pred.	$R_{pred/obs}$	Pred.	$R_{pred/obs}$[c]	Pred.	$R_{pred/obs}$	Pred.	$R_{pred/obs}$[c]
Ziprasidone 5 mg-iv											
**C_{max} (ng/mL) geomean (CV%)**	83 (21)	114 (13)	1.4	90.7 (15)	1.1	90.1 (14.3)	1.1	n/a	n/a	85.6 (14.4)	1.0
**AUC_{inf} (ng.h/mL) geomean (CV%)**	217 (20)	1900 (45.7)	8.8	273 (31.4)	1.3	265 (27.7)	1.2	n/a	n/a	220 (25)	1.0
**CL_{iv} (L/h) mean (CV%)**	23.04 (14)	2.89 (44.7)	0.1	19.2 (30.1)	0.8	19.5 (26.0)	0.8	n/a	n/a	23.4 (23.5)	1.0
**fm_{AO}**	67	n/a	n/a	n/a	n/a	66.4 (21.2)	1.0	n/a	n/a	66.5 (21.3)	1.0
**fm_{CYP3A4}**	33	n/a	n/a	n/a	n/a	33.6 (39.0)	1.0	n/a	n/a	33.5 (39.1)	1.0
Ziprasidone 20 mg-oral											
**C_{max} (ng/mL) geomean (CV%)**	64 (28)	126 (30.5)	2.0	50.1 (40.8)	0.8	49.7 (39.4)	0.8	n/a	n/a	40.5 (41.2)	0.6
**AUC_{inf} (ng.h/mL) geomean (CV%)**	514 (27)	4686 (53.2)	9.1	452 (53.1)	0.9	446 (50.4)	0.9	n/a	n/a	325 (50.4)	0.6
**CL_{oral} (L/h) mean (CV%)**	38.9 (27)	4.81 (48.4)	0.1	49.8 (48.4)	1.3	50.3 (53)	1.3	n/a	n/a	69.1 (53.0)	1.8
**Fg (mean)**	n/a	1	n/a	1	n/a	0.99	n/a	n/a	n/a	0.99	n/a
Zoniporide 80 mg-iv											
**C_{max} (ng/mL) mean (CV%)**	590 (7.49)	933 (11.2)	1.6	691 (11.5)	1.2	690 (11.4)-691 (11.4)	1.2	528 (12.2)	0.9	538 (12.1)-529 (12.2)	0.9
**AUC_{inf} (ng.h/mL) mean (CV%)**	839 (4.08)	2227 (17.9)	2.7	1076 (13.4)	1.3	1073 (12.7)-1075 (13.2)	1.3	699 (12.7)	0.8	719 (12.6)-702 (12.7)	0.9-0.8
**CL_{iv} (L/h) mean (CV%)**	95.4 (5.71)	37 (17.3)	0.4	75.6 (12.9)	0.8	75.7 (12.3)-75.7 (12.7)	0.8	116.3 (12.6)	1.2	113.1 (12.6)-115.8 (12.6)	1.2
**fm_{AO}**	52-69	37.4 (25.1)	0.6	68 (11.3)	1.1	48.5 (22)-64.1 (13.4)	0.8-1.1	76.5 (12.8)	1.3	58.5 (23.3)-73.5 (14.4)	1.0-1.2
**fm_{other}**	13-30	11.84 (33.9)	0.6	9.1 (45.8)	0.4	28.72 (31)-13.07 (42.4)	1.3-0.6	9.17 (48.8)	0.4	26.83 (34.5)-12.15 (46.1)	1.2-1.4

Abbreviations: AUC_{inf}, area under curve extrapolated to infinity, $CL_{int,u}$, hepatic unbound intrinsic clearance, CL_{iv}, total plasma clearance after intraveneous administration, CL_{oral}, total plasma clearance after oral administration CI, confidence interval, C_{max}, maximum systemic concentration, CV, coefficient of variation, F, female, fm_{AO}, fraction metabolized by aldehyde oxidase, HH, human hepatocytes, M, male, n/a, not available, Obs, observed, Pred, predicted, $R_{pred/obs}$, ratio of predicted to observed

[a]Pharmacokinetic parameters were represented as mean or geometric mean with CV% or geometric CV%, respectively.

[b]Two predicted values (separated with dash) were given when simulations were performed with minimum and maximum fm_{AO} assumptions.

[c]For $R_{pred/obs}$ of fm_i, mean of predicted values were considered when simulations were performed with minimum and maximum $fm_{i.}$

Table S12. Evaluation of model performances

		Bottom-up model		Bottom-up model with ESF		Middle-out model with obs fm_{AO}		Middle-out model with obs $CL_{int,u}$		Top-down model
		HLM-HLC	HH	HLM-HLC	HH	HLM-HLC	HH	HLM-HLC	HH	
	**Number of simulations**	11	11	11	11	18	18	11	9	18
	**Number of compounds**	6	6	6	6	6	6	6	5	6
**C_{max}**	**% points out of twofold range**	18.2	27.3	9.1	0	5.6	0	9.1	11.1	11.1
	**AFE**	1.4	1.5	0.9	0.9	0.9	0.9	0.8	0.8	0.8
	**GMFE**	1.4	1.6	1.3	1.2	1.2	1.2	1.3	1.3	1.3
	**RMSE (ng/mL)**	732	1191	478	351	423	329	300	300	263
**AUC_{inf}**	**% points out of twofold range**	90.9	100	0	0	0	0	0	0	5.6
	**AFE**	3.5	5.7	1.1	1.0	1.1	1.1	0.9	0.9	0.9
	**GMFE**	3.5	5.7	1.4	1.2	1.4	1.3	1.2	1.2	1.2
	**RMSE (ng.h/mL)**	13093	43727	1862	1485	1740	1238	707	1219	648
**CL_{Total}**	**% points out of twofold range**	90.9	100	0	0	0	0	9.1	11.1	11.1
	**AFE**	0.3	0.2	0.9	1.0	0.9	0.9	1.2	1.1	1.2
	**GMFE**	3.3	5.1	1.4	1.2	1.3	1.3	1.2	1.2	1.2
	**RMSE (L/h)**	69.9	78.2	19.3	12.5	18.8	28	85	77	98.9

Abbreviations: AFE, average fold error, $CL_{int,u}$, hepatic unbound intrinsic clearance, fm_{AO}, fraction metabolized by aldehyde oxidase, GMFE, geometric mean fold error, HH, human hepatocytes, HLC, human liver cytosols, HLM, human liver microsomes, ESF, empirical scaling factor, RMSE, root mean squared error.

Supplement 8. Model verification and CYP3A4- drug-drug interaction prediction

Content:

Figure S12. Observed vs. predicted CYP3A4-drug drug interactions by the bottom-up model with empirical scaling factors from human liver microsomes and cytosols

Figure S13. Observed vs. predicted CYP3A4-drug drug interactions by the bottom-up model with empirical scaling factors from human hepatocytes

Figure S14. Observed vs. predicted CYP3A4-drug drug interactions by using the middle-out model from human liver microsomes and cytosols with observed fraction metabolized

Figure S15. Observed vs. predicted CYP3A4-drug drug interactions by using the middle-out model from human hepatocytes with observed fraction metabolized

Figure S16. Observed vs. predicted CYP3A4-drug drug interactions by the middle-out model from human liver microsomes and cytosols with observed intrinsic clearance data

Figure S17. Observed vs. predicted CYP3A4-drug drug interactions by the middle-out model from human hepatocytes with observed intrinsic clearance data

Figure S18. Observed vs. predicted CYP3A4-drug drug interactions by the top-down model with observed fraction metabolized data and observed intrinsic clearance data

Table S13. Summary of observed vs. predicted pharmacokinetic parameters of study compounds in the presence and absence of CYP3A4 perpetrators with bottom-up PBPK models using the data from human liver microsomes and cytosols and refined models

Table S14. Summary of observed vs. predicted pharmacokinetic parameters of study compounds in the presence and absence of CYP3A4 perpetrators with bottom-up PBPK models using the data from human hepatocytes (with and without hydralazine) and refined models

Table S15. Evaluation of model performances predicting independent CYP3A4-DDI studies

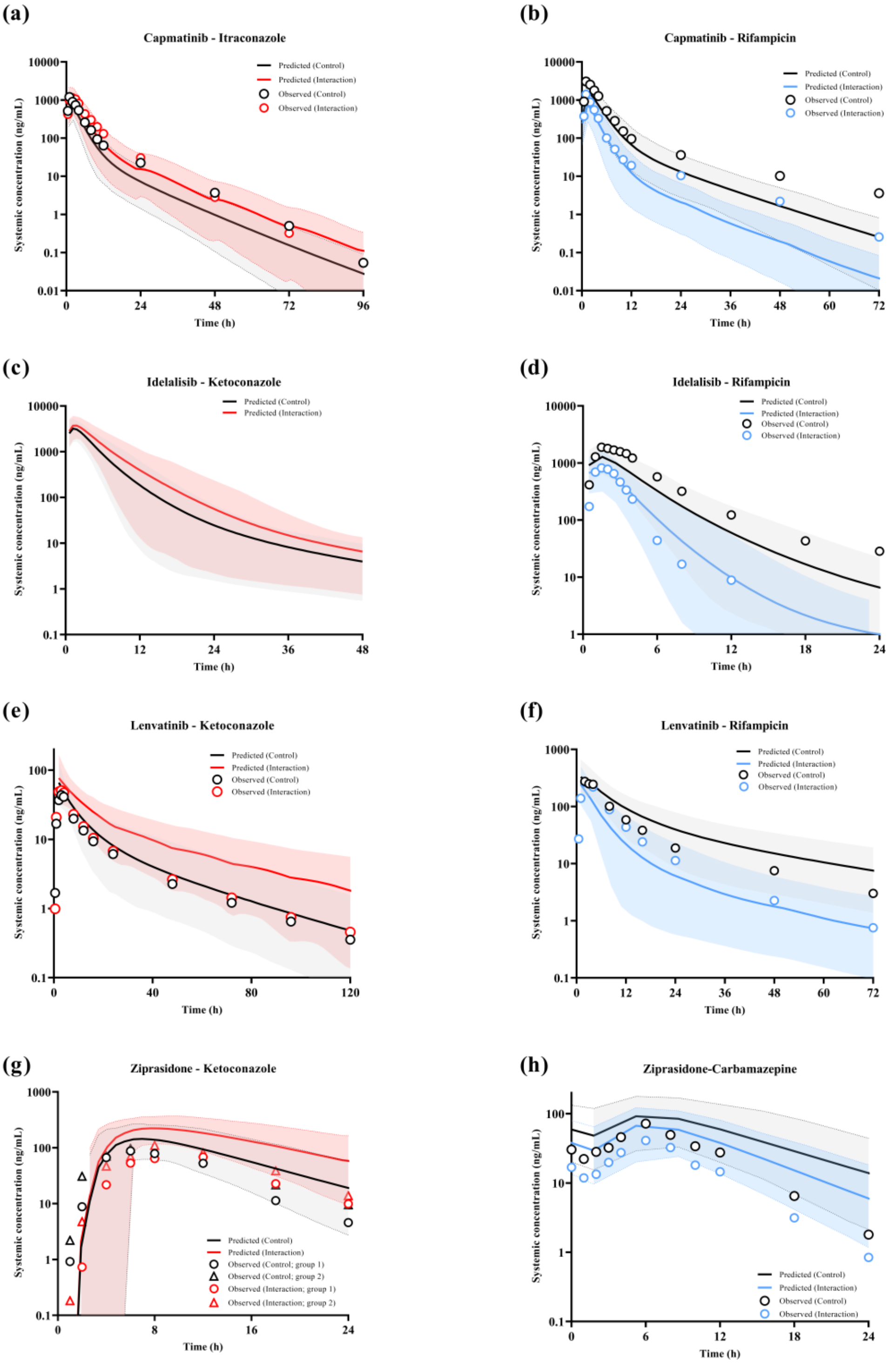

Figure S12. Simulation of the DDI between a) capmatinib [400 mg] and itraconazole [200 mg MD], b) capmatinib [200 mg] and rifampicin [600 mg MD], c) idelalisib [400 mg] and ketoconazole [400 mg MD], d) idelalisib [150 mg] and rifampicin [600 mg], e) lenvatinib [5 mg] and ketoconazole [400 mg MD], f) lenvatinib [24 mg] and rifampicin [600 mg], g) ziprasidone [40 mg] and ketoconazole [200-400 mg MD], h) ziprasidone [20 mg] and carbamazepine [200-400 mg] using the bottom-up model with empirical scaling factors from human liver microsomes and cytosols. Solid lines represent the predicted profile. Symbols (circle and square) represent observed data, if available. Area between the dotted lines representing the 5th and 95th percentile of predictions is shaded with matching colours to predicted lines. In the simulations of ziprasidone-ketoconazole interaction, 5th percentile included 0 for time points below 6h.

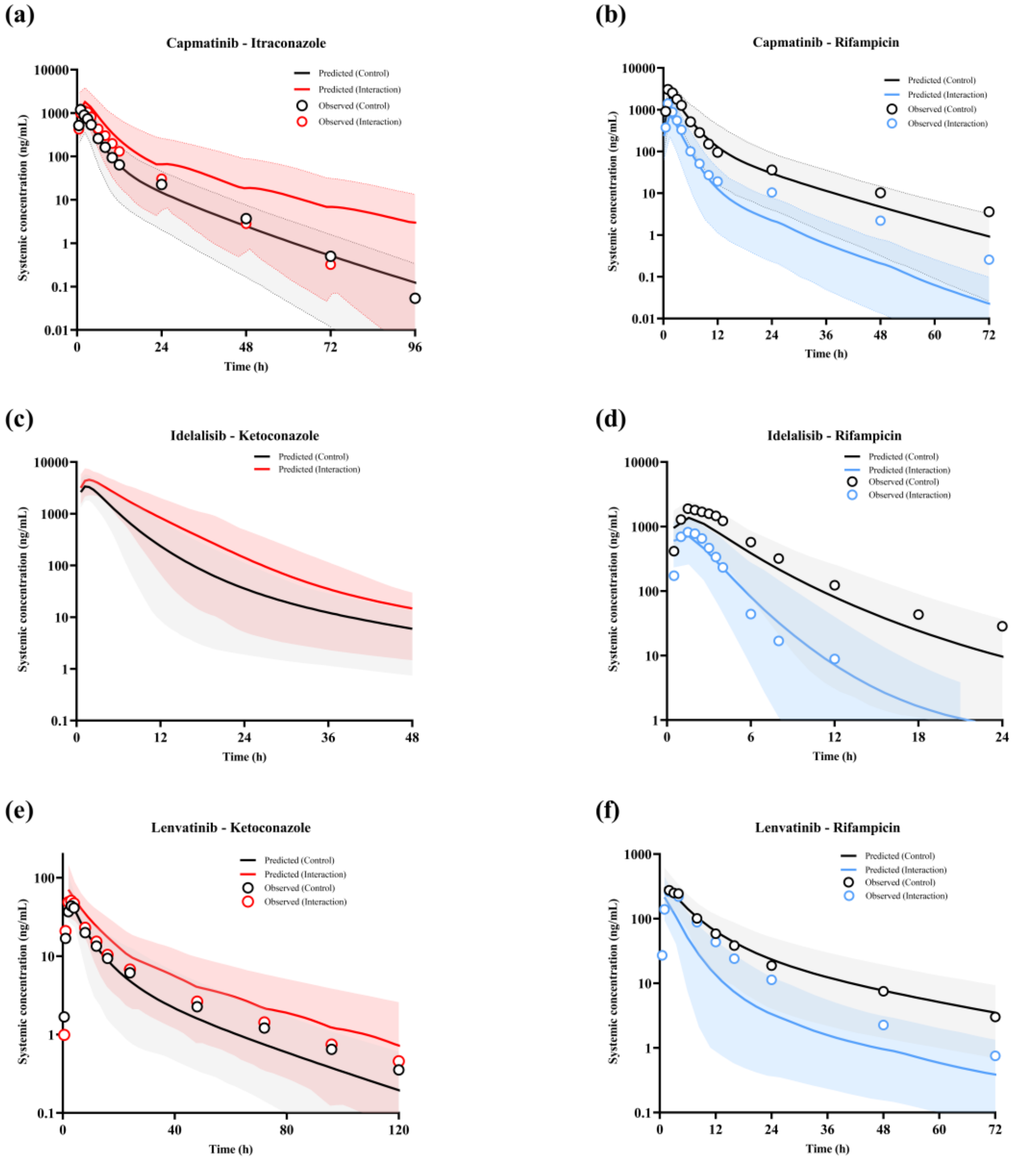

Figure S13. Simulation of the DDI between a) capmatinib [400 mg] and itraconazole [200 mg MD], b) capmatinib [200 mg] and rifampicin [600 mg MD], c) idelalisib [400 mg] and ketoconazole [400 mg MD], d) idelalisib [150 mg] and rifampicin [600 mg], e) lenvatinib [5 mg] and ketoconazole [400 mg MD], f) lenvatinib [24 mg] and rifampicin [600 mg], using the bottom-up model with empirical scaling factors from human hepatocytes. Solid lines represent the predicted profile. Symbols (circle and square) represent observed data, if available. Area between the dotted lines representing the 5th and 95th percentile of predictions is shaded with matching colours to predicted lines.

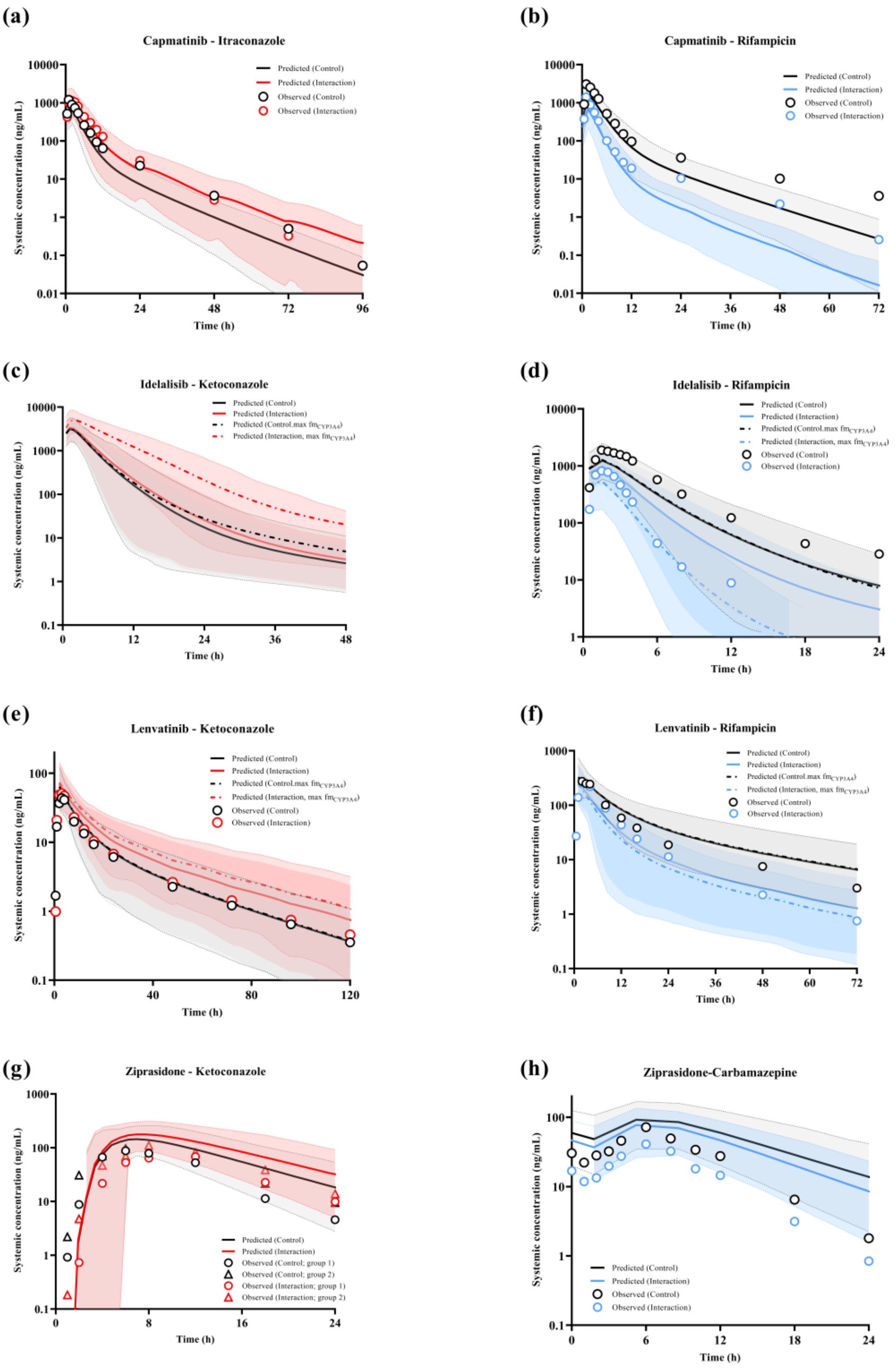

Figure S14. Simulation of the DDI between a) capmatinib [400 mg] and itraconazole [200 mg MD], b) capmatinib [200 mg] and rifampicin [600 mg MD], c) idelalisib [400 mg] and ketoconazole [400 mg MD], d) idelalisib [150 mg] and rifampicin [600 mg], e) lenvatinib [5 mg] and ketoconazole [400 mg MD], f) lenvatinib [24 mg] and rifampicin [600 mg], g) ziprasidone [40 mg] and ketoconazole [200-400 mg MD], h) ziprasidone [20 mg] and carbamazepine [200-400 mg] using the middle-out model from human liver microsomes and cytosols with observed fraction metabolized data. Solid and dashed line represent the predicted profiles with minimum and maximum fm_{AO} /fm_{CYP3A4} assumption, if available. Symbols (circle and square) represent observed data, if available. Area between the dotted lines representing the 5th and 95th percentile of predictions is shaded with matching colours to predicted lines. In the simulations of ziprasidone-ketoconazole interaction, 5th percentile included 0 for time points below 6h.

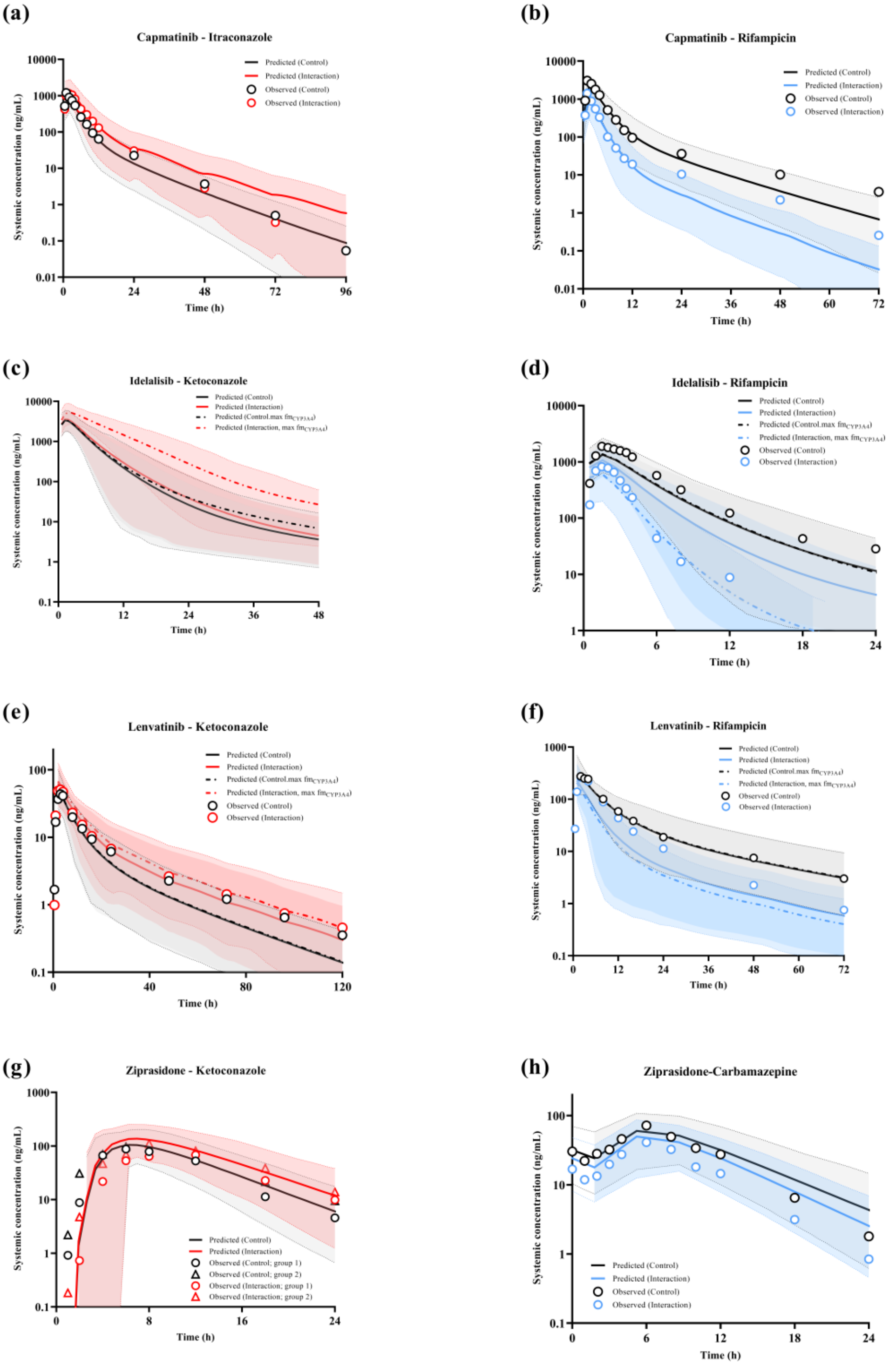

Figure S15. Simulation of the DDI between a) capmatinib [400 mg] and itraconazole [200 mg MD], b) capmatinib [200 mg] and rifampicin [600 mg MD], c) idelalisib [400 mg] and ketoconazole [400 mg MD], d) idelalisib [150 mg] and rifampicin [600 mg], e) lenvatinib [5 mg] and ketoconazole [400 mg MD], f) lenvatinib [24 mg] and rifampicin [600 mg], g) ziprasidone [40 mg] and ketoconazole [200-400 mg MD], h) ziprasidone [20 mg] and carbamazepine [200-400 mg] using the middle-out model from human hepatocytes with observed fraction metabolized data. Solid and dashed line represent the predicted profiles with minimum and maximum fm_{AO} /fm_{CYP3A4} assumption, if available. Symbols (circle and square) represent observed data, if available. Area between the dotted lines representing the 5th and 95th percentile of predictions is shaded with matching colours to predicted lines. In the simulations of ziprasidone-ketoconazole interaction, 5th percentile included 0 for time points below 6h.

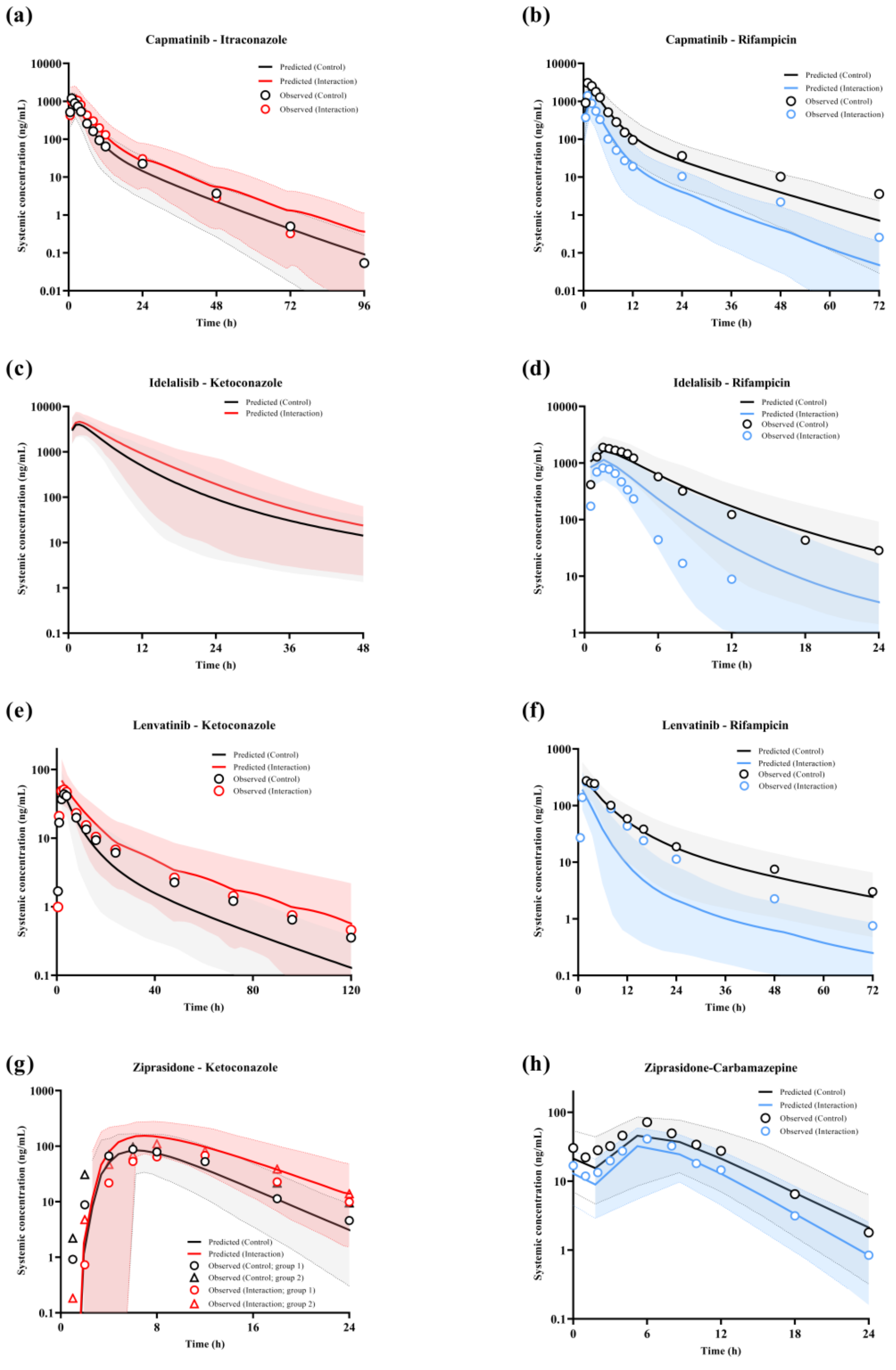

Figure S16. Simulation of the DDI between a) capmatinib [400 mg] and itraconazole [200 mg MD], b) capmatinib [200 mg] and rifampicin [600 mg MD], c) idelalisib [400 mg] and ketoconazole [400 mg MD], d) idelalisib [150 mg] and rifampicin [600 mg], e) lenvatinib [5 mg] and ketoconazole [400 mg MD], f) lenvatinib [24 mg] and rifampicin [600 mg], g) ziprasidone [40 mg] and ketoconazole [200-400 mg MD], h) ziprasidone [20 mg] and carbamazepine [200-400 mg] using the middle-out model from human liver microsomes and cytosols with observed intrinsic clearance data. Solid lines represent the predicted profile. Symbols (circle and square) represent observed data, if available. Area between the dotted lines representing the 5th and 95th percentile of predictions is shaded with matching colours to predicted lines. In the simulations of ziprasidone-ketoconazole interaction, 5th percentile included 0 for time points below 6h.

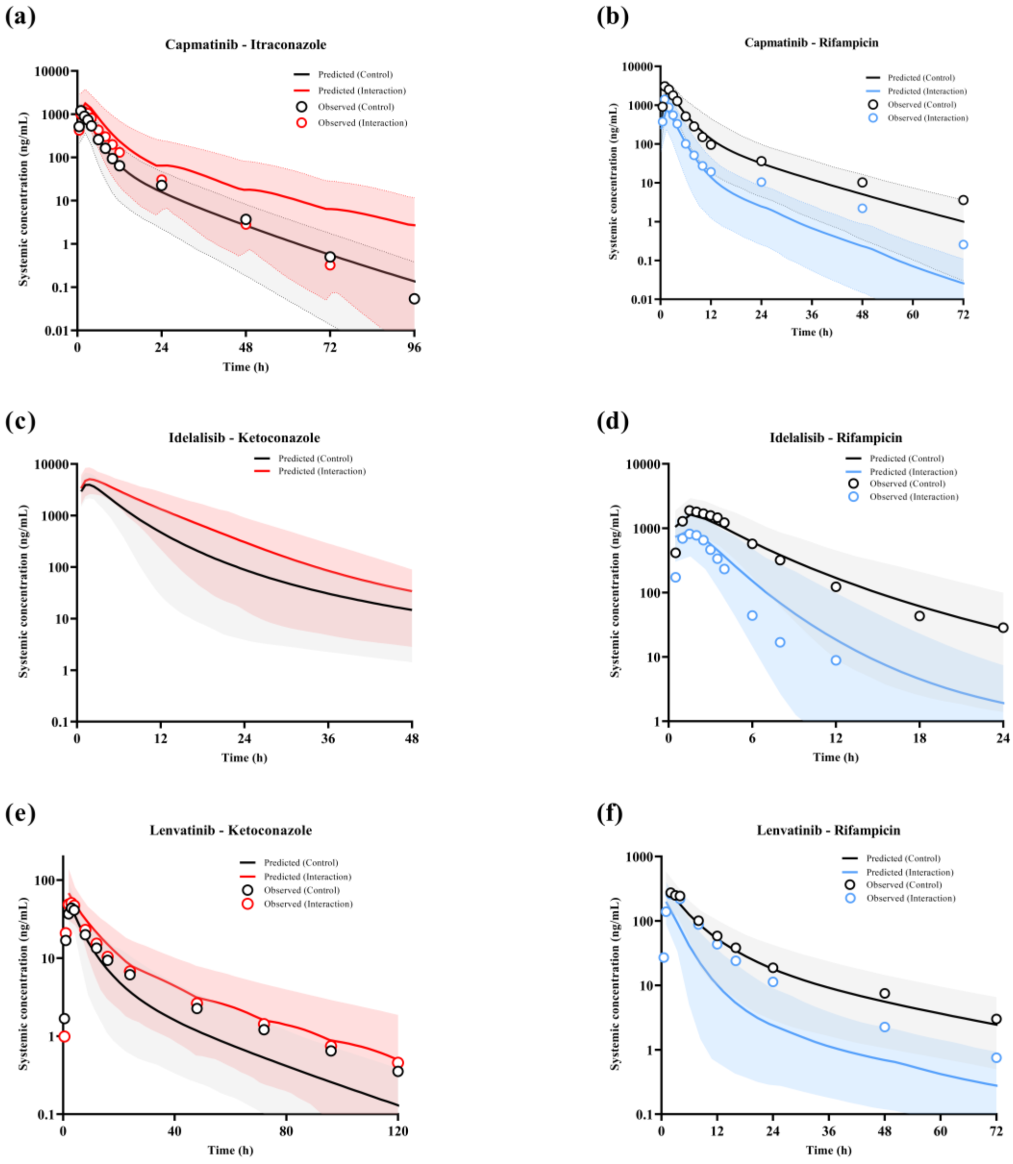

Figure S17. Simulation of the DDI between a) capmatinib [400 mg] and itraconazole [200 mg MD], b) capmatinib [200 mg] and rifampicin [600 mg MD], c) idelalisib [400 mg] and ketoconazole [400 mg MD], d) idelalisib [150 mg] and rifampicin [600 mg], e) lenvatinib [5 mg] and ketoconazole [400 mg MD], f) lenvatinib [24 mg] and rifampicin [600 mg] using the middle-out model from human hepatocytes with observed intrinsic clearance data. Solid lines represent the predicted profile. Symbols (circle and square) represent observed data, if available. Area between the dotted lines representing the 5th and 95th percentile of predictions is shaded with matching colours to predicted lines.

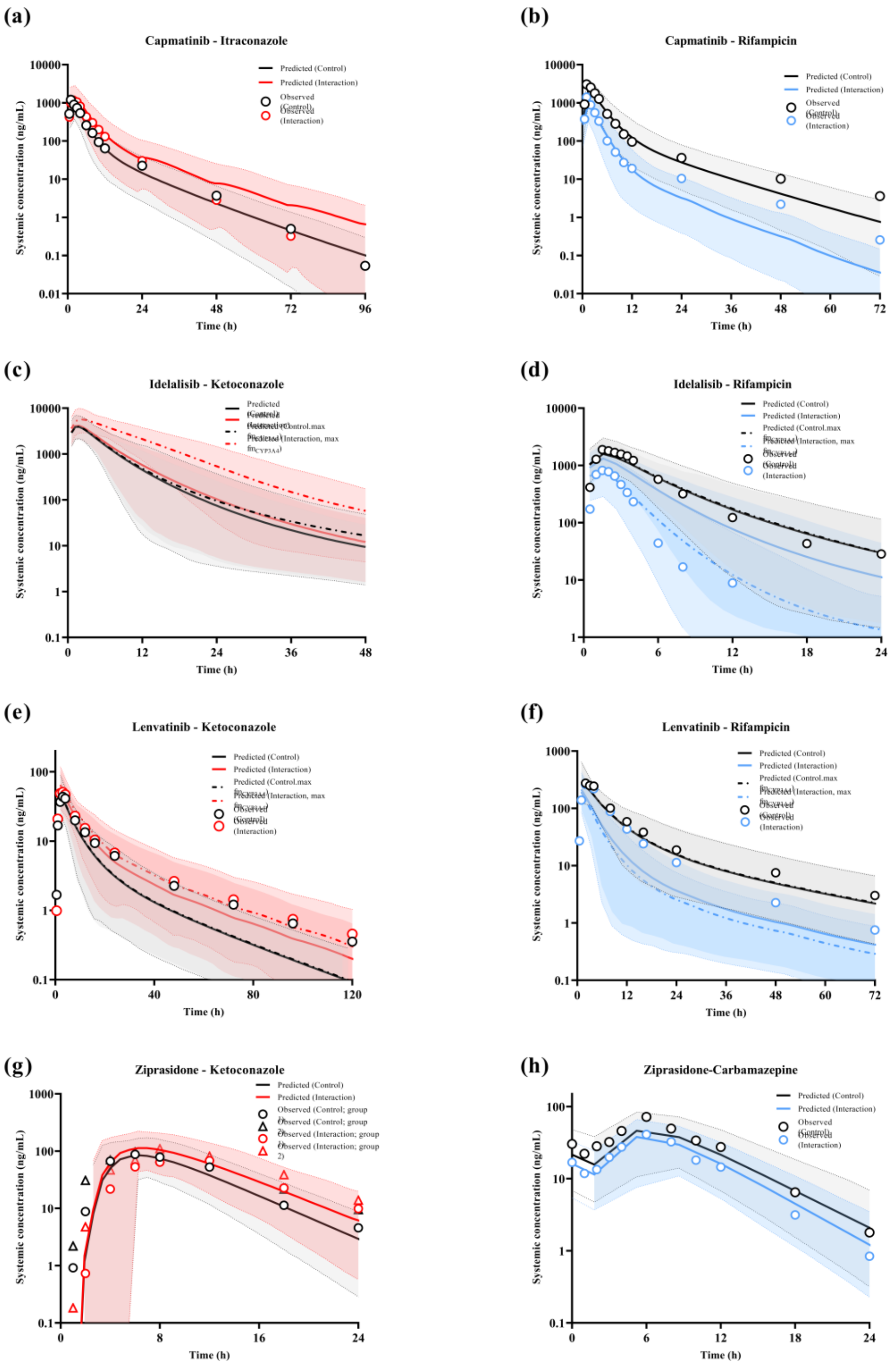

Figure S18. Simulation of the DDI between a) capmatinib [400 mg] and itraconazole [200 mg MD], b) capmatinib [200 mg] and rifampicin [600 mg MD], c) idelalisib [400 mg] and ketoconazole [400 mg MD], d) idelalisib [150 mg] and rifampicin [600 mg], e) lenvatinib [5 mg] and ketoconazole [400 mg MD], f) lenvatinib [24 mg] and rifampicin [600 mg], g) ziprasidone [40 mg] and ketoconazole [200-400 mg MD], h) ziprasidone [20 mg] and carbamazepine [200-400 mg] using the top-down model with observed fraction metabolized data and observed intrinsic clearance data. Solid and dashed line represent the predicted profiles with minimum and maximum fm_{AO} /fm_{CYP3A4} assumption, if available. Symbols (circle and square) represent observed data, if available. Area between the dotted lines representing the 5th and 95th percentile of predictions is shaded with matching colours to predicted lines. In the simulations of ziprasidone-ketoconazole interaction, 5th percentile included 0 for time points below 6h.

Table S13. Summary of observed vs. predicted pharmacokinetic parameters of study compounds in the presence and absence of CYP3A4 perpetrators with bottom-up PBPK models using the data from human liver microsomes and cytosols and refined models.

PK parameters[a]	Obs.	Bottom-up model with ESF (from HLM-HLC)		Middle-out model with obs fm_{AO} (from HLM-HLC)[b]		Middle-out model with obs $CL_{int,u}$ (from HLM-HLC)		Top-down model with obs fm_{AO} and $CL_{int,u}$ [b]	
		Pred.	$R_{pred/obs}$	Pred.	$R_{pred/obs}$	Pred.	$R_{pred/obs}$	Pred.	$R_{pred/obs}$
**Capmatinib-Itraconazole DDI**									
$C_{max, control}$ (ng/mL) geomean (CV%)	1260	743 (49)	0.6	732 (50)	0.6	915 (50)	0.7	903 (50)	0.7
$AUC_{inf, control}$ (ng.h/mL) geomean (CV%)	5180	2955 (47)	0.6	2904 (49)	0.6	4197 (47)	0.8	4130 (49)	0.8
$C_{max, interaction}$ (ng/mL) geomean (CV%)	1300	1080 (54)	0.8	1193 (56)	0.9	1278 (55)	1.0	1400 (57)	1.1
$AUC_{inf, interaction}$ (ng.h/mL) geomean (CV%)	7360	4899 (54)	0.7	5659 (57)	0.8	6771 (54)	0.9	7797 (57)	1.1
AUCR (+perpetrator/control) Geometric mean (90% CI)	1.42 (1.33-1.52)	1.66 (1.63-1.69)	1.2	1.95 (1.91-1.99)	1.4	1.61 (1.59-1.64)	1.1	1.89 (1.85-1.93)	1.3
**Capmatinib-Rifampicin DDI**									
$C_{max, control}$ (ng/mL) geomean (CV%)	3070	1655 (51)	0.5	1634 (51)	0.5	2071 (51)	0.7	2048 (52)	0.7
$AUC_{inf, control}$ (ng.h/mL) geomean (CV%)	11500	6323 (47)	0.5	6263 (50)	0.5	9020 (48)	0.8	8962 (50)	0.8
$C_{max, interaction}$ (ng/mL) geomean (CV%)	1350	774 (61)	0.6	680 (65)	0.5	1020 (60)	0.8	901 (64)	0.7
$AUC_{inf, interaction}$ (ng.h/mL) geomean (CV%)	3850	2165 (65)	0.6	1845 (69)	0.5	3102 (65)	0.8	2638 (70)	0.7
AUCR (+perpetrator/control) Geometric mean (90% CI)	0.44 (0.39-0.50)	0.34 (0.33-0.36)	0.8	0.29 (0.28-0.31)	0.7	0.34 (0.33-0.36)	0.8	0.29 (0.28-0.31)	0.7
**Idelalisib-Ketoconazole DDI**									
$C_{max, control}$ (ng/mL) geomean (CV%)	3550	3056 (38)	0.9	2991 (39)-2992 (39)	0.8	3839 (37)	1.1	3771 (39)-3780 (38)	1.1
$AUC_{inf, control}$ (ng.h/mL) geomean (CV%)	15900	14369 (45)	0.9	13813 (41)-13983 (50)	0.9	24000 (44)	1.5	23096 (40)-23447 (49)	1.5
$C_{max, interaction}$ (ng/mL) geomean (CV%)	4450	3588 (39)	0.8	3200 (39)-4772 (38)	0.7-1.1	4367 (38)	1.0	3974 (39)-5368 (39)	0.9-1.2
$AUC_{inf, interaction}$ (ng.h/mL) geomean (CV%)	28500	20240 (51)	0.7	15823 (42)-40896 (42)	0.6-1.4	33557 (48)	1.2	26267 (41)-61252 (39)	0.9-2.1
AUCR (+perpetrator/control) Geometric mean (90% CI)	1.79 (1.57-2.04)	1.41 (1.37-1.45)	0.8	1.15 (1.13-1.16)-2.92(2.76-3.10)	0.6-1.6	1.40 (1.36-1.44)	0.8	1.14 (1.13-1.15)-2.61 (2.48-2.76)	0.6-1.5

Cont. PK parameters[a]	Obs.	Bottom-up model with ESF (from HLM-HLC)		Middle-out model with obs fm_{AO} (from HLM-HLC)[b]		Middle-out model with obs $CL_{int,u}$ (from HLM-HLC)		Top-down model with obs fm_{AO} and $CL_{int,u}$ [b]	
		Pred.	$R_{pred/obs}$	Pred.	$R_{pred/obs}$	Pred.	$R_{pred/obs}$	Pred.	$R_{pred/obs}$
**Idelalisib-Rifampicin DDI**									
$C_{max, control}$ (ng/mL) mean (CV%)	2152 (24)	1325 (42)	0.6	1281 (41)-1311 (44)	0.6	1665 (41)	0.8	1613 (40) -1653 (42)	0.7-0.8
$AUC_{inf, control}$ (ng.h/mL) mean (CV%)	9599 (37)	5685 (46)	0.6	5568 (45)-5685 (52)	0.6	9613 (44)	1.0	9422 (44) - 9640 (50)	1.0
$C_{max, interaction}$ (ng/mL) mean (CV%)	933 (41)	886 (51)	1.0	1033 (43)-607 (66)	1.1-0.7	1188 (48)	1.3	1358 (41) - 873 (60)	1.5-0.9
$AUC_{inf, interaction}$ (ng.h/mL) mean (CV%)	2294 (40)	2695 (55)	1.2	3698 (49)- 1581 (73)	1.6-0.7	4510 (54)	2.0	62856 (49) - 2741 (72)	2.7-1.2
AUCR (+perpetrator/control) Geometric mean (90% CI)	0.25 (0.23-0.27)	0.46 (0.43-0.48)	1.8	0.65 (0.63-0.68) – 0.25 (0.24-0.27)	2.6-1.0	0.45 (0.43-0.48)	1.8	0.66 (0.63-0.68) - 0.26 (0.24-0.27)	2.6-1.0
**Lenvatinib-Ketoconazole DDI**									
$C_{max, control}$ (ng/mL) geomean (CV%)	43.7 (26)	62.1 (52)	1.4	60.8 (55)-61.1 (56)	1.4	55.0 (50)	1.3	53.5 (53)-53.8 (54)	1.2
$AUC_{inf, control}$ (ng.h/mL) geomean (CV%)	577 (17)	758 (40)	1.3	683 (40)-697 (39)	1.2	437 (42)	0.8	393 (43)-401 (41)	0.7
$C_{max, interaction}$ (ng/mL) geomean (CV%)	51.2 (38)	69.1 (54)	1.3	64.7 (56)- 67.1 (57)	1.3	64.3 (52)	1.3	58.5 (54)-61.6 (56)	1.1-1.2
$AUC_{inf, interaction}$ (ng.h/mL) geomean (CV%)	662 (21)	1408 (43)	2.1	936 (41)- 1142 (37)	1.4-1.7	851 (44)	1.3	550 (43)-681 (38)	0.8-1.0
AUCR (+perpetrator/control) Geometric mean (90% CI)	1.15 (1.08-1.21)	1.86 (1.79-1.92)	1.6	1.37 (1.34-1.39)- 1.64 (1.60-1.68)	1.2-1.4	1.95 (1.87-2.02)	1.7	1.40 (1.37-1.43)- 1.70 (1.65-1.75	1.2-1.5
**Lenvatinib-Rifampicin DDI**									
$C_{max, control}$ (ng/mL) geomean (CV%)	274 (36.2)	303 (52)	1.1	297 (53)-298 (53)	1.1	269 (51)	1.0	262 (52)-263 (51)	1.0
$AUC_{inf, control}$ (ng.h/mL) geomean (CV%)	2370 (24.2)	3610 (41)	1.5	3291 (41)-3351 (40)	1.4	2071 (42)	0.9	1885 (43)-1919 (42)	0.8
$C_{max, interaction}$ (ng/mL) geomean (CV%)	275 (28.6)	218 (53)	0.8	239 (51)-221 (51)	0.9-0.8	173 (55)	0.6	195 (51)-176 (51)	0.7-0.6
$AUC_{inf, interaction}$ (ng.h/mL) geomean (CV%)	1950 (27.1)	1012 (63)	0.5	1338 (56)-1055 (63)	0.7-0.5	567 (65)	0.3	753 (59)-591 (65)	0.4-0.3
AUCR (+perpetrator/control) Geometric mean (90% CI)	0.82 (0.73-0.91)	0.28 (0.27-0.30)	0.3	0.41 (0.39-0.43)-0.31 (0.30-0.33)	0.5-0.4	0.27 (0.26-0.29)	0.3	0.40 (0.38-0.42)- 0.31 (0.29-0.32)	0.5-0.4

Cont. PK parameters[a]	Obs.	Bottom-up model with ESF (from HLM-HLC)		Middle-out model with obs fm_{AO} (from HLM-HLC)[b]		Middle-out model with obs $CL_{int,u}$ (from HLM-HLC)		Top-down model with obs fm_{AO} and $CL_{int,u}$ [b]	
		Pred.	$R_{pred/obs}$	Pred.	$R_{pred/obs}$	Pred.	$R_{pred/obs}$	Pred.	$R_{pred/obs}$
**Ziprasidone-Ketoconazole DDI**									
$C_{max, control}$ (ng/mL) geomean (CV%)	89 (30)	146 (39)	1.6	147 (37)	1.6	88 (43)	1.0	88.9 (41)	1.0
$AUC_{inf, control}$ (ng.h/mL) geomean (CV%)	899 (20)	1553 (53)	1.7	1553 (50)	1.7	666 (53)	0.7	670 (50)	0.7
$C_{max, interaction}$ (ng/mL) geomean (CV%)	119 (25)	221 (34)	1.9	178 (36)	1.5	156 (38)	1.3	117 (42)	1.0
$AUC_{inf, interaction}$ (ng.h/mL) geomean (CV%)	1199 (18)	3395 (52)	2.8	2208 (53)	1.8	1547 (52)	1.3	987 (54)	0.8
AUCR (+perpetrator/control) Geometric mean (90% CI)	1.33 (1.12-1.58)	2.19 (2.11-2.26)	1.6	1.42 (1.39-1.45)	1.1	2.32 (2.24-2.41)	1.7	1.47 (1.44-1.51)	1.1
**Ziprasidone-Carbamazepine DDI**									
$C_{max, control}$ (ng/mL) geomean (CV%)	79 (24)	93.7 (43)	1.2	95.2 (41)	1.2	48.2 (43)	0.6	49.2 (40)	0.6
$AUC_{0-12, control}$ (ng.h/mL) mean (CV%)	495 (26)	876 (51)	1.8	881 (47)	1.8	376 (51)	0.8	381 (48)	0.8
$C_{max, interaction}$ (ng/mL) geomean (CV%)	48 (27)	70.3 (40)	1.5	81.0 (39)	1.7	34.7 (40)	0.7	40.9 (40)	0.9
$AUC_{0-12, interaction}$ (ng.h/mL) mean (CV%)	285 (28)	601 (44)	2.1	713 (43)	2.5	249 (44)	0.9	300 (43)	1.1
AUCR (+perpetrator/control) Geometric mean (90% CI)	0.58 (0.44-0.75)	0.70 (0.69-0.71)	1.2	0.82 (0.81-0.83)	1.4	0.68 (0.67-0.69)	1.2	0.80 (0.79-0.81)	1.4

Abbreviations: AUC_{0-12}, area under curve between zero to twelve hours, AUC_{inf}, area under curve extrapolated to infinity, $CL_{int,u}$, hepatic unbound intrinsic clearance, CI, confidence interval, C_{max}, maximum systemic concentration, CV, coefficient of variation, fm_{AO}, fraction metabolized by aldehyde oxidase, HLC, human liver cytosols, HLM, human liver microsomes, n/a, not available, Obs, observed, Pred, predicted, $R_{pred/obs}$, ratio of predicted to observed

[a]Pharmacokinetic parameters were represented as mean or geometric mean with CV% or geometric CV%, respectively.

[b]Two predicted values (separated with dash) were given when simulations were performed with minimum and maximum fm_{AO} assumptions.

Table S14. Summary of observed vs. predicted pharmacokinetic parameters of study compounds in the presence and absence of CYP3A4 perpetrators with bottom-up PBPK models using the data from human hepatocytes (with and without hydralazine) and refined models

PK parameters[a]	Obs.	Bottom-up model with ESF (from HH)		Middle-out model with obs fm_{AO} (from HH)[b]		Middle-out model with obs $CL_{int,u}$ (from HH)		Top-down model with obs fm_{AO} and $CL_{int,u}$[b]	
		Pred.	$R_{pred/obs}$	Pred.	$R_{pred/obs}$	Pred.	$R_{pred/obs}$	Pred.	$R_{pred/obs}$
**Capmatinib-Itraconazole DDI**									
**$C_{max, control}$ (ng/mL) geomean (CV%)**	1260	868 (53)	0.7	882 (50)	0.7	890 (52)	0.7	903 (50)	0.7
**$AUC_{inf, control}$ (ng.h/mL) geomean (CV%)**	5180	3914 (58)	0.8	3968 (49)	0.8	4078 (55)	0.8	4130 (49)	0.8
**$C_{max, interaction}$ (ng/mL) geomean (CV%)**	1300	1676 (62)	1.3	1376 (57)	1.1	1664 (62)	1.3	1400 (57)	1.1
**$AUC_{inf, interaction}$ (ng.h/mL) geomean (CV%)**	7360	10561 (73)	1.4	7518 (57)	1.0	10503 (70)	1.4	7797 (57)	1.1
**AUCR (+perpetrator/control) Geometric mean (90% CI)**	1.42 (1.33-1.52)	2.70 (2.62-2.78)	1.9	1.89 (1.86-1.93)	1.3	2.58 (2.50-2.65)	1.8	1.89 (1.85-1.93)	1.3
**Capmatinib-Rifampicin DDI**									
**$C_{max, control}$ (ng/mL) geomean (CV%)**	3070	1974 (54)	0.6	1998 (52)	0.7	2026 (54)	0.7	2048 (52)	0.7
**$AUC_{inf, control}$ (ng.h/mL) geomean (CV%)**	11500	8647 (58)	0.8	8602 (50)	0.7	9001 (58)	0.8	8962 (50)	0.8
**$C_{max, interaction}$ (ng/mL) geomean (CV%)**	1350	727 (69)	0.5	873 (64)	0.6	763 (68)	0.6	901 (64)	0.7
**$AUC_{inf, interaction}$ (ng.h/mL) geomean (CV%)**	3850	2012 (77)	0.5	2533 (70)	0.7	2138 (76)	0.6	2638 (70)	0.7
**AUCR (+perpetrator/control) Geometric mean (90% CI)**	0.44 (0.39-0.50)	0.23 (0.22-0.24)	0.5	0.29 (0.28-0.31)	1.5	0.24 (0.23-0.25)	0.5	0.29 (0.28-0.31)	0.7
**Idelalisib-Ketoconazole DDI**									
**$C_{max, control}$ (ng/mL) geomean (CV%)**	3550	3233 (38)	0.9	3207 (39)-3210 (39)	0.9	3803 (37)	1.1	3771 (39)-3780 (38)	1.1
**$AUC_{inf, control}$ (ng.h/mL) geomean (CV%)**	15900	16222 (45)	1.0	15939 (41)-16152 (50)	1.0	23544 (45)	1.5	23096 (40)-23447 (49)	1.5
**$C_{max, interaction}$ (ng/mL) geomean (CV%)**	4450	4315 (38)	1.0	3417 (39)-4950 (38)	0.8-1.1	4801 (38)	1.1	3974 (39)-5368 (39)	0.9-1.2
**$AUC_{inf, interaction}$ (ng.h/mL) geomean (CV%)**	28500	31618 (47)	1.1	18228 (42)-45918 (41)	0.6-1.6	43618 (44)	1.5	26267 (41)-61252 (39)	0.9-2.1
**AUCR (+perpetrator/control) Geometric mean (90% CI)**	1.79 (1.57-2.04)	1.95 (1.86-2.04)	1.1	1.14 (1.13-1.16)-2.84 (2.69-3.01)	0.6-1.6	1.85 (1.78-1.93)	1.0	1.14 (1.13-1.15)-2.61 (2.48-2.76)	0.6-1.5

Cont. PK parameters[a]	Obs.	Bottom-up model with ESF (from HLM-HLC)		Middle-out model with obs fm_{AO} (from HLM-HLC)[b]		Middle-out model with obs $CL_{int,u}$ (from HLM-HLC)		Top-down model with obs fm_{AO} and $CL_{int,u}$ [b]	
		Pred.	$R_{pred/obs}$	Pred.	$R_{pred/obs}$	Pred.	$R_{pred/obs}$	Pred.	$R_{pred/obs}$
**Idelalisib-Rifampicin DDI**									
**$C_{max, control}$ (ng/mL) mean (CV%)**	2152 (24)	1406 (42)	0.7	1373 (41)-1405 (44)	0.6-0.7	1654 (41)	0.8	1613 (40) -1653 (42)	0.7-0.8
**$AUC_{inf, control}$ (ng.h/mL) mean (CV%)**	9599 (37)	6483 (47)	0.7	6450 (44)-6590 (52)	0.7	9485 (46)	1.0	9422 (44) - 9640 (50)	1.0
**$C_{max, interaction}$ (ng/mL) mean (CV%)**	933 (41)	784 (58)	0.8	1121 (42)-675 (64)	1.2-0.7	1001 (60)	1.1	1358 (41) - 873 (60)	1.5-0.9
**$AUC_{inf, interaction}$ (ng.h/mL) mean (CV%)**	2294 (40)	2268 (64)	1.0	4288 (48)- 1844 (73)	1.9-0.8	3379 (63)	1.5	62856 (49) - 2741 (72)	2.7-1.2
**AUCR (+perpetrator/control) Geometric mean (90% CI)**	0.25 (0.23-0.27)	0.32 (0.30-0.34)	1.3	0.65 (0.63-0.68)- 0.25 (0.24-0.27)	2.6-1.0	0.33 (0.31-0.35)	1.3	0.66 (0.63-0.68) - 0.26 (0.24-0.27)	2.6-1.0
**Lenvatinib-Ketoconazole DDI**									
**$C_{max, control}$ (ng/mL) geomean (CV%)**	43.7 (26)	57.5 (50)	1.3	55.9 (54)-56.2 (54)	1.3	55.2 (50)	1.3	53.5 (53)-53.8 (54)	1.2
**$AUC_{inf, control}$ (ng.h/mL) geomean (CV%)**	577 (17)	523 (40)	0.9	465 (42)-475 (40)	0.8	442 (41)	0.8	393 (43)-401 (41)	0.7
**$C_{max, interaction}$ (ng/mL) geomean (CV%)**	51.2 (38)	65.1 (52)	1.3	60.5 (55)-63.4 (56)	1.2	63.5 (52)	1.2	58.5 (54)-61.6 (56)	1.1-1.2
**$AUC_{inf, interaction}$ (ng.h/mL) geomean (CV%)**	662 (21)	930 (44)	1.4	646 (42)-797 (37)	1.0-1.2	801 (45)	1.2	550 (43)-681 (38)	0.8-1.0
**AUCR (+perpetrator/control) Geometric mean (90% CI)**	1.15 (1.08-1.21)	1.78 (1.72-1.84)	1.6	1.39 (1.36-1.42)- 1.68 (1.64-1.73)	1.2-1.5	1.81 (1.75-1.88)	1.6	1.40 (1.37-1.43)- 1.70 (1.65-1.75	1.2-1.5
**Lenvatinib-Rifampicin DDI**									
**$C_{max, control}$ (ng/mL) geomean (CV%)**	274 (36.2)	281 (51)	1.0	273 (52)-274 (52)	1.0	270 (51)	1.0	262 (52)-263 (51)	1.0
**$AUC_{inf, control}$ (ng.h/mL) geomean (CV%)**	2370 (24.2)	2478 (41)	1.0	2235 (42)-2275 (41)	0.9-1.0	2090 (42)	0.9	1885 (43)-1919 (42)	0.8
**$C_{max, interaction}$ (ng/mL) geomean (CV%)**	275 (28.6)	194 (54)	0.7	208 (51)-189 (51)	0.8-.0.7	179 (54)	0.7	195 (51)-176 (51)	0.7-0.6
**$AUC_{inf, interaction}$ (ng.h/mL) geomean (CV%)**	1950 (27.1)	737 (62)	0.4	897 (58)-705 (64)	0.5-0.4	613 (63)	0.3	753 (59)-591 (65)	0.4-0.3
**AUCR (+perpetrator/control) Geometric mean (90% CI)**	0.82 (0.73-0.91)	0.30 (0.28-0.31)	0.4	0.40 (0.38-0.42)-0.31 (0.29-0.33)	0.5-0.4	0.29 (0.28-0.31)	0.4	0.40 (0.38-0.42)- 0.31 (0.29-0.32)	0.5-0.4

Cont. PK parameters[a]	Obs.	Bottom-up model with ESF (from HLM-HLC)		Middle-out model with obs fm_{AO} (from HLM-HLC)[b]		Middle-out model with obs $CL_{int,u}$ (from HLM-HLC)		Top-down model with obs fm_{AO} and $CL_{int,u}$ [b]	
		Pred.	$R_{pred/obs}$	Pred.	$R_{pred/obs}$	Pred.	$R_{pred/obs}$	Pred.	$R_{pred/obs}$
**Ziprasidone-Ketoconazole DDI**									
$C_{max,\ control}$ (ng/mL) geomean (CV%)	89 (30)	n/a	n/a	109 (40)	1.2	n/a	n/a	88.9 (41)	1.0
$AUC_{inf,\ control}$ (ng.h/mL) geomean (CV%)	899 (20)	n/a	n/a	921 (50)	1.0	n/a	n/a	670 (50)	0.7
$C_{max,\ interaction}$ (ng/mL) geomean (CV%)	119 (25)	n/a	n/a	139 (40)	1.2	n/a	n/a	117 (42)	1.0
$AUC_{inf,\ interaction}$ (ng.h/mL) geomean (CV%)	1199 (18)	n/a	n/a	1338 (54)	1.1	n/a	n/a	987 (54)	0.8
AUCR (+perpetrator/control) Geometric mean (90% CI)	1.33 (1.12-1.58)	n/a	n/a	1.45 (1.42-1.49)	1.1	n/a	n/a	1.47 (1.44-1.51)	1.1
**Ziprasidone-Carbamazepine DDI**									
$C_{max,\ control}$ (ng/mL) geomean (CV%)	79 (24)	n/a	n/a	63.2 (41)	0.8	n/a	n/a	49.2 (40)	0.6
$AUC_{0-12,\ control}$ (ng.h/mL) mean (CV%)	495 (26)	n/a	n/a	523 (48)	1.1	n/a	n/a	381 (48)	0.8
$C_{max,\ interaction}$ (ng/mL) geomean (CV%)	48 (27)	n/a	n/a	53.2 (39)	1.1	n/a	n/a	40.9 (40)	0.9
$AUC_{0-12,\ interaction}$ (ng.h/mL) mean (CV%)	285 (28)	n/a	n/a	417 (43)	1.5	n/a	n/a	300 (43)	1.1
AUCR (+perpetrator/control) Geometric mean (90% CI)	0.58 (0.44-0.75)	n/a	n/a	0.81 (0.80-0.82)	1.4	n/a	n/a	0.80 (0.79-0.81)	1.4

Abbreviations: AUC_{0-12}, area under curve between zero to twelve hours, AUC_{inf}, area under curve extrapolated to infinity, CI, confidence interval, $CL_{int,u}$, hepatic unbound intrinsic clearance, C_{max}, maximum systemic concentration, CV, coefficient of variation, fm_{AO}, fraction metabolized by aldehyde oxidase, HH, human hepatocytes, n/a, not available, Obs, observed, Pred, predicted, $R_{pred/obs}$, ratio of predicted to observed

[a]Pharmacokinetic parameters were represented as mean or geometric mean with CV% or geometric CV%, respectively.

[b]Two predicted values (separated with dash) were given when simulations were performed with minimum and maximum fm_{AO} assumptions.

Table S15. Evaluation of model performances predicting independent CYP3A4-DDI studies

		Bottom-up model with ESF		Middle-out model with obs fm_{AO}		Middle-out model with obs $CL_{int,u}$		Top-down model
		HLM-HLC	HH	HLM-HLC	HH	HLM-HLC	HH	
	**Number of simulations**	8	6	12	12	8	6	12
	**Number of compounds**	4	3	4	4	4	3	4
**Model verification**								
$C_{max,control}$	**% points out of 2-fold range**	0	0	0	0	0	0	0
	**AFE**	0.9	0.8	0.9	0.9	0.9	0.9	0.9
	**GMFE**	1.4	1.3	1.4	1.3	1.3	1.3	1.3
	**RMSE (ng/mL)**	633	579	608	473	424	506	389
$AUC_{inf,control}$	**% points out of 2-fold range**	0	0	0	0	0	0	8.3
	**AFE**	1.0	0.8	1.0	0.9	0.9	0.9	0.8
	**GMFE**	1.6	1.2	1.5	1.2	1.3	1.2	1.4
	**RMSE (ng.h/mL)**	2539	1806	2508	1553	3019	3317	3121
**Verification of fm_{CYP3A4}**								
**AUCR (+perpetrator/control)**	**% points out of Guest et al. criteria**	38	50	33	33	38	50	25
	**AFE**	1.0	1.0	1.0	1.0	1.0	1.0	1.0
	**GMFE**	1.5	1.7	1.5	1.6	1.6	1.7	1.5
	**RMSE**	0.47	0.63	0.50	0.48	0.52	0.59	0.45

[a]Based on predictive measure considering variability by Guest et al.[49]

Abbreviations: AFE, average fold error, $CL_{int,u}$, hepatic unbound intrinsic clearance, fm_{AO}, fraction metabolized by aldehyde oxidase, GMFE, geometric mean fold error, HH, human hepatocytes, HLC, human liver cytosols, HLM, human liver microsomes, ESF, empirical scaling factor, RMSE, root mean squared error.

Supplement 9. Inhibitory effects of AO and CYP3A4 inhibitors

Content:

Table S16. Inhibition potencies of ketoconazole and itraconazole on: AO and CYP3A4

Table S17. Evaluation of clinical DDI risk of AO inhibitors

Table S16. Inhibition potencies of ketoconazole and itraconazole on AO and CYP3A4

Parameter	Itraconazole	Ketoconazole
**IC50 (μM) for AO inhibition**	>100[50,a]	3.8[50,a]-5.2[51,b]
**fu_{mic} (0.5 mg/mL microsomal protein)**	0.014[52]	0.37[52]
**$IC50_u$ (μM) for AO inhibition**	>2.8	1.2 - 2.1
**$IC50_u$ (μM) for CYP3A4 inhibition**	0.0013[c]	0.01[c]

[a]*In vitro* inhibition of AO-mediated metabolism of zaleplon in human liver cytosols (0.25 mg/mL)

[b]*In vitro* inhibition of AO-mediated metabolism of ziprasidone in human liver S9 (1 mg/mL)

[c]Simcyp simulator (version 21; Certara, Sheffield, UK).

Table S17. Evaluation of clinical DDI risk of AO inhibitors

	Mode of inhibition	In vitro inhibition constant (μM)	Clinical evidence	Inhibition of other enzymes	Comments
**Cimetidine**	Competitive[30]	K_i= 155[30]	No/weak DDIs for dual AO-CYP3A4 substrates ziprasidone (fm_{AO}= 0.67; [AUCR= 1.06[53]]) and zaleplon (fm_{AO}= 0.57-0.74; [AUCR=1.85[54]])	Inhibits CYP3A4 (K_i= 370 μM)[55]	• As cimetidine is a weak AO and CYP3A4 inhibitor, the mechanism behind observed clinical interaction with zaleplon has raised questions[29]
**Ethinyl estradiol**	Mixed-mode inhibition *(K_{ii}/K_{is}= 0.21)*[31]	K_i= 1.1[31]	n/a	n/a	• Ethinyl estradiol is typically taken at very low doses in oral contraception combinations, thus the hepatic inlet/plasma concentrations stay below the K_i.<br>• Similar low DDI risk was also shown for other estrogenic compounds (e.g. β-estradiol)[31]
**Erlotinib**	Time-dependent and reversible inhibition[32]	K_i= 0.14[32] (IC50/2)<br>K_I= 0.72[32]<br>k_{inact}= 0.36 1/min[32]	A clinical interaction (AUCR~2-fold[56]) between erlotinib (150 mg QD) and OSI-930 (fm_{AO}=0.46[29])	Inhibits UGT1A1 (IC50= 1.34 μM) and UGT1A3 (K_i= 3.02 μM)[32]	• Erlotinib predominantly inhibits AO via time-dependent inhibition[29].<br>• Predicted AUCR of 1.85 for OSI-930 (fm_{AO}=0.46) consistent with clinical data and insensitive to k_{deg} across a range of values reported for CYPs.[29] However, $k_{deg,AO}$ value should be refined by monitoring the recovery of AO to improve the ability to predict AO time-dependent inhibition.<br>• Caution should be taken when AO substrates are co-administrated with erlotinib.<br>• High incidence of adverse effects by erlotinib observed in multiple-dose studies in healthy individuals[57] precludes the conduct of routine dedicated clinical DDI studies with this inhibitor.
**Icotinib**	Time-dependent and reversible inhibition[32]	K_i= 0.36[32] (IC50/2)<br>K_I= 1.18[32]<br>k_{inact}= 0.55 1/min[32]	n/a	Inhibits UGT1A1 (IC50= 12.2 μM) and UGT1A3 (Ki= 25.6 μM)[32]	• There is no evidence of clinical interaction so far. However, caution should be taken when AO substrates are co-administrated with icotinib based on the semi-mechanistic model predictions.<br>• Although icotinib is well tolerated in single dose studies, incidence of adverse effects increased in multiple dose studies[58]. This may preclude the conduct of routine dedicated clinical DDI studies with this inhibitor.
**Raloxifene**	Uncompetitive inhibition of oxidative reactions (e.g. phthalazine, nicotine, vanillin)[59]<br>Competitive inhibition of DACA oxidation[35]<br>Noncompetitive inhibition of CP-544,439 reduction[59]	K_i= 0.87-1.4 nM[59]<br>K_i= 2.3 nM[35]<br>K_i= 51 nM[59]	No clinical DDI was observed (AUCR=0.85) between favipiravir (fm_{AO}=0.9) and raloxifene (60 mg QD)[60]	Weakly inhibits xanthine oxidase (XO) as only caused 58% inhibition of oxidation of XO substrate pterin[59]	• Raloxifene shows substrate-specific mode of inhibition with a range of in vitro K_i. Therefore, substrate-specific K_i values should be used in model predictions.

Supplementary Material References

1. Sun D, Lennernas H, Welage LS, et al. Comparison of Human Duodenum and Caco-2 Gene Expression Profiles for 12,000 Gene Sequences Tags and Correlation with Permeability of 26 Drugs. *Pharm Res.* 2002;19(10):1400-1416. doi:10.1023/A:1020483911355
2. Gertz M, Kilford PJ, Houston JB, Galetin A. Drug Lipophilicity and Microsomal Protein Concentration as Determinants in the Prediction of the Fraction Unbound in Microsomal Incubations. *Drug Metab Dispos*. 2008;36(3):535-542. doi:10.1124/dmd.107.018713
3. Yang J, Jamei M, Yeo KR, Tucker GT, Rostami-Hodjegan A. Prediction of Intestinal First-pass Drug Metabolism. *Curr Drug Metab*. 2007;8(7):676-84. doi:10.2174/138920007782109733
4. Ezuruike U, Zhang M, Pansari A, et al. Guide to Development of Compound Files for PBPK Modeling in The Simcyp Population-Based Simulator. *CPT Pharmacometrics Syst Pharmacol*. 2022;11(7):805-821. doi:10.1002/psp4.12791
5. Rodgers T, Rowland M. Physiologically Based Pharmacokinetic Modelling 2: Predicting The Tissue Distribution of Acids, Very Weak Bases, Neutrals and Zwitterions. *J Pharm Sci.* 2006/06/01/ 2006;95(6):1238-1257. doi:10.1002/jps.20502
6. Poulin P, Theil FP. Prediction of Pharmacokinetics Prior to In vivo Studies. 1. Mechanism-based Prediction of Volume of Distribution. *J Pharm Sci*. 2002;91(1):129-56. doi:10.1002/jps.10005
7. Berezhkovskiy LM. Determination of Volume of Distribution at Steady State with Complete Consideration of The Kinetics of Protein and Tissue Binding in Linear Pharmacokinetics. *J Pharm Sci*. 2004;93(2):364-74. doi:10.1002/jps.10539
8. Glaenzel U, Jin Y, Hansen R, et al. Absorption, Distribution, Metabolism, and Excretion of Capmatinib (INC280) in Healthy Male Volunteers and In Vitro Aldehyde Oxidase Phenotyping of the Major Metabolite. *Drug Metab Dispos*. 2020;48(10):873-885. doi:10.1124/dmd.119.090324
9. Jin F, Robeson M, Zhou H, Kwan E, Ramanathan S. Pharmacokinetics, Metabolism and Excretion Of Idelalisib. *Blood*. 2013;122(21):5570-5570. doi:10.1182/blood.V122.21.5570.5570
10. FDA. Zydelig, idelalisib, Clinical Pharmacology NDA Review, https://www.accessdata.fda.gov/drugsatfda_docs/nda/2014/206545Orig1s000ClinPharmR.pdf. 2014
11. FDA. Lenvima, lenvatinib, Clinical Pharmacology NDA Review, https://www.accessdata.fda.gov/drugsatfda_docs/nda/2015/206947orig1s000clinpharmr.pdf. 2015
12. Prakash C, Kamel A, Gummerus J, Wilner K. Metabolism and Excretion of a New Antipsychotic Drug, Ziprasidone, in Humans. *Drug Metab Dispos*. 1997;25(7):863-72.
13. Dalvie D, Zhang C, Chen W, Smolarek T, Obach RS, Loi CM. Cross-species Comparison of The Metabolism and Excretion of Zoniporide: Contribution of Aldehyde Oxidase to Interspecies Differences. *Drug Metab Dispos*. 2010;38(4):641-54. doi:10.1124/dmd.109.030783
14. Chen X, Cui X, Pognan N, et al. Pharmacokinetics of Capmatinib in Participants with Hepatic Impairment: A Phase 1, Open-label, Single-dose, Parallel-group Study. *Br J Clin Pharmacol*. 2022;88(1):91-102. doi:10.1111/bcp.14929
15. Cui X, Chen X, Pognan N, et al. Evaluation of the Pharmacokinetic Drug Interaction of Capmatinib With Itraconazole and Rifampicin and Potential Impact on Renal Transporters in Healthy Subjects. *J Clin Pharmacol*. 2023;63(2):228-238. doi:10.1002/jcph.2153
16. Jin F, Robeson M, Zhou H, Hisoire G, Ramanathan S. The Pharmacokinetics and Safety of Idelalisib in Subjects with Moderate or Severe Hepatic Impairment. *J Clin Pharmacol*. 2015;55(8):944-52. doi:10.1002/jcph.504
17. Webb HK, Chen H, Yu AS, et al. Clinical Pharmacokinetics of CAL-101, a p110δ Isoform-Selective PI3K Inhibitor, Following Single- and Multiple-Dose Administration In Healthy Volunteers and Patients with Hematological Malignancies. *Blood*. 2010;116(21):1774-1774. doi:10.1182/blood.V116.21.1774.1774
18. Jin F, Robeson M, Zhou H, et al. Clinical Drug Interaction Profile of Idelalisib in Healthy Subjects. *J Clin Pharmacol*. 2015;55(8):909-19. doi:10.1002/jcph.495

19. Shumaker R, Aluri J, Fan J, Martinez G, Pentikis H, Ren M. Influence of Hepatic Impairment on Lenvatinib Pharmacokinetics Following Single-dose Oral Administration. *J Clin Pharmacol*. 2015;55(3):317-27. doi:10.1002/jcph.398
20. Shumaker R, Aluri J, Fan J, Martinez G, Thompson GA, Ren M. Effects of Ketoconazole on the Pharmacokinetics of Lenvatinib (E7080) in Healthy Participants. *Clin Pharmacol Drug Dev*. 2015;4(2):155-160. doi:10.1002/cpdd.140
21. Shumaker RC, Aluri J, Fan J, Martinez G, Thompson GA, Ren M. Effect of Rifampicin on The Pharmacokinetics of Lenvatinib in Healthy Adults. *Clin Drug Investig*. 2014;34(9):651-9. doi:10.1007/s40261-014-0217-y
22. Rosen AS, Fournié P, Darwish M, Danjou P, Troy SM. Zaleplon Pharmacokinetics and Absolute Bioavailability. *Biopharm Drug Dispos* 1999;20(3):171-175. doi:10.1002/(SICI)1099-081X(199904)20:3<171::AID-BDD169>3.0.CO;2-K
23. Miceli JJ, Wilner KD, Swan SK, Tensfeldt TG. Pharmacokinetics, Safety, and Tolerability of Intramuscular Ziprasidone in Healthy Volunteers. *J Clin Pharmacol*. 2005;45(6):620-30. doi:10.1177/0091270005276485
24. Miceli JJ, Smith M, Robarge L, Morse T, Laurent A. The Effects of Ketoconazole on Ziprasidone Pharmacokinetics--A Placebo-controlled Crossover Study in Healthy Volunteers. *Br J Clin Pharmacol*. 2000;49 (Suppl 1):71s-76s. doi:10.1046/j.1365-2125.2000.00156.x
25. Miceli JJA, R.J. Robarge, L. Hansen, R.A., Laurent, A. The Effect of Carbamazepine on The Steady-state Pharmacokinetics of Ziprasidone in Healthy Volunteers. *Br J Clin Pharmacol*. 2000;49 (Suppl. 1):65S-70S.
26. Fahmi OA, Hurst S, Plowchalk D, et al. Comparison of different algorithms for predicting clinical drug-drug interactions, based on the use of CYP3A4 in vitro data: predictions of compounds as precipitants of interaction. *Drug Metab Dispos*. 2009;37(8):1658-66. doi:10.1124/dmd.108.026252
27. Kirby BJ, Unadkat JD. Impact of Ignoring Extraction Ratio When Predicting Drug-Drug Interactions, Fraction Metabolized, and Intestinal First-Pass Contribution. *Drug Metab Dispos*. 2010;38(11):1926-1933. doi:10.1124/dmd.110.034736
28. Yang J, Liao M, Shou M, et al. Cytochrome p450 turnover: regulation of synthesis and degradation, methods for determining rates, and implications for the prediction of drug interactions. *Curr Drug Metab*. 2008;9(5):384-94. doi:10.2174/138920008784746382
29. Tang LWT, Shi Y, Sharma R, Obach RS. The Drug-Drug Interaction Between Erlotinib and OSI-930 is Mediated Through Aldehyde Oxidase Inhibition. *Drug Metab Dispos*. 2024;doi:10.1124/dmd.124.001802
30. Renwick AB, Ball SE, Tredger JM, et al. Inhibition of zaleplon metabolism by cimetidine in the human liver: in vitro studies with subcellular fractions and precision-cut liver slices. *Xenobiotica*. 2002;32(10):849-62. doi:10.1080/00498250210158221
31. Barr JT, Jones JP. Inhibition of human liver aldehyde oxidase: implications for potential drug-drug interactions. *Drug Metab Dispos*. Dec 2011;39(12):2381-6. doi:10.1124/dmd.111.041806
32. Tang LWT, DaSilva E, Lapham K, Obach RS. Evaluation of Icotinib as a Potent and Selective Inhibitor of Aldehyde Oxidase for Reaction Phenotyping in Human Hepatocytes. *Drug Metab Dispos*. 2024:DMD-AR-2024-001693. doi:10.1124/dmd.124.001693
33. Wishart DS, Feunang YD, Guo AC, et al. DrugBank 5.0: A Major Update to The DrugBank Database for 2018. *Nucleic Acids Res*. 2018;46(D1):D1074-d1082. doi:10.1093/nar/gkx1037
34. Hallifax D, Houston JB. Binding of drugs to hepatic microsomes: comment and assessment of current prediction methodology with recommendation for improvement. *Drug Metab Dispos*. 2006;34(4):724-6; author reply 727. doi:10.1124/dmd.105.007658
35. Barr JT, Jones JP. Evidence for Substrate-dependent Inhibition Profiles for Human Liver Aldehyde Oxidase. *Drug Metab Dispos*. 2013;41(1):24-9. doi:10.1124/dmd.112.048546

36. Cubitt HE, Houston JB, Galetin A. Prediction of Human Drug Clearance by Multiple Metabolic Pathways: Integration of Hepatic and Intestinal Microsomal and Cytosolic Data. *Drug Metab Dispos*. 2011;39(5):864-73. doi:10.1124/dmd.110.036566
37. Walkenstein SS, Dubb JW, Randolph WC, Westlake WJ, Stote RM, Intoccia AP. Bioavailability of cimetidine in man. *Gastroenterology*. 1978;74(2, Part 2):360-365. doi:https://doi.org/10.1016/0016-5085(78)90762-X
38. Fattore C, Cipolla G, Gatti G, et al. Induction of ethinylestradiol and levonorgestrel metabolism by oxcarbazepine in healthy women. *Epilepsia*. 1999;40(6):783-7. doi:10.1111/j.1528-1157.1999.tb00779.x
39. Traina TA, Poggesi I, Robson M, et al. Pharmacokinetics and Tolerability of Exemestane in Combination with Raloxifene in Postmenopausal Women with A History of Breast Cancer. *Breast Cancer Res Treat*. 2008;111(2):377-88. doi:10.1007/s10549-007-9787-1
40. Yamamoto N, Horiike A, Fujisaka Y, et al. Phase I dose-finding and pharmacokinetic study of the oral epidermal growth factor receptor tyrosine kinase inhibitor Ro50-8231 (erlotinib) in Japanese patients with solid tumors. *Cancer Chemother Pharmacol*. 2008;61(3):489-96. doi:10.1007/s00280-007-0494-8
41. Ni J, Liu D-Y, Hu B, et al. Relationship between icotinib hydrochloride exposure and clinical outcome in Chinese patients with advanced non–small cell lung cancer. *Cancer*. 2015;121(S17):3146-3156. doi:https://doi.org/10.1002/cncr.29568
42. Bayer. Yasmin, drospirenone/ethinyl estradiol, US Prescribing Information, https://www.accessdata.fda.gov/drugsatfda_docs/label/2012/021098s022lbl.pdf. 2001;
43. Eli Lilly and Company, Raloxifene United States Prescription Information, https://pi.lilly.com/us/evista-pi.pdf. 2022
44. Cheng X, Lv X, Qu H, et al. Comparison of the inhibition potentials of icotinib and erlotinib against human UDP-glucuronosyltransferase 1A1. *Acta Pharm Sin B*. 2017;7(6):657-664. doi:10.1016/j.apsb.2017.07.004
45. Obach RS, Prakash C, Kamel AM. Reduction and Methylation of Ziprasidone by Glutathione, Aldehyde Oxidase, and Thiol S-methyltransferase In Humans: An In vitro Study. *Xenobiotica*. 2012;42(11):1049-1057. doi:10.3109/00498254.2012.683203
46. Subash S, Singh DK, Ahire DS, et al. Dissecting Parameters Contributing to the Underprediction of Aldehyde Oxidase-Mediated Metabolic Clearance of Drugs. *Drug Metab Dispos*. 2023;51(10):1362-1371. doi:10.1124/dmd.123.001379
47. Izat N, Bolleddula J, Abbasi A, et al. Challenges and Opportunities for In Vitro-In Vivo Extrapolation of Aldehyde Oxidase-Mediated Clearance: Toward a Roadmap for Quantitative Translation. *Drug Metab Dispos*. 2023;51(12):1591-1606. doi:10.1124/dmd.123.001436
48. Wood FL, Houston JB, Hallifax D. Clearance Prediction Methodology Needs Fundamental Improvement: Trends Common to Rat and Human Hepatocytes/Microsomes and Implications for Experimental Methodology. *Drug Metab Dispos*. 2017;45(11):1178-1188. doi:10.1124/dmd.117.077040
49. Guest EJ, Aarons L, Houston JB, Rostami-Hodjegan A, Galetin A. Critique of The Two-Fold Measure of Prediction Success for Ratios: Application for The Assessment of Drug-Drug Interactions. *Drug Metab Dispos*. 2011;39(2):170-3. doi:10.1124/dmd.110.036103
50. Buckley DB, Ogilvie BW, Yerino P. The In vitro Evaluation of Ketoconazole and Its Alternative Clinical CYP3A4/5 Inhibitors (ritonavir, clarithromycin and itraconazole) as Inhibitors of non-CYP Enzymes. *Drug Metab Pharmacokinet*. 2017;1(32):S54.
51. Obach RS, Walsky RL. Drugs That Inhibit Oxidation Reactions Catalyzed by Aldehyde Oxidase Do Not Inhibit The Reductive Metabolism of Ziprasidone to Its Major Metabolite, S-methyldihydroziprasidone: An In vitro Study. *J Clin Psychopharmacol*. 2005;25(6):605-8. doi:10.1097/01.jcp.0000186740.22395.50
52. Gardner I, Xu M, Han C, et al. Non-specific Binding of Compounds in In vitro Metabolism Assays: A Comparison of Microsomal and Hepatocyte Binding in Different Species and An

Assessment of The Accuracy of Prediction Models. *Xenobiotica*. 2022;52(8):943-956. doi:10.1080/00498254.2022.2132426
53. Wilner KD, Hansen RA, Folger CJ, Geoffroy P. The Pharmacokinetics of Ziprasidone in Healthy Volunteers Treated with Cimetidine or Antacid. *Br J Clin Pharmacol*. 2000;49 (Suppl 1):57s-60s. doi:10.1046/j.1365-2125.2000.00154.x
54. Pfizer. Sonata (zaleplon) US Prescribing Information, https://labeling.pfizer.com/ShowLabeling.aspx?id=710. 2023
55. Akiyoshi T, Saito T, Murase S, et al. Comparison of the inhibitory profiles of itraconazole and cimetidine in cytochrome P450 3A4 genetic variants. *Drug Metab Dispos*. 2011;39(4):724-8. doi:10.1124/dmd.110.036780
56. Macpherson IR, Poondru S, Simon GR, et al. A phase 1 study of OSI-930 in combination with erlotinib in patients with advanced solid tumours. *Eur J Cancer*. 2013;49(4):782-9. doi:10.1016/j.ejca.2012.09.036
57. Ling J, Fettner S, Lum BL, Riek M, Rakhit A. Effect of food on the pharmacokinetics of erlotinib, an orally active epidermal growth factor receptor tyrosine-kinase inhibitor, in healthy individuals. *Anticancer Drugs*. 2008;19(2):209-16. doi:10.1097/CAD.0b013e3282f2d8e4
58. Liu J, Wu L, Wu G, et al. A Phase I Study of the Safety and Pharmacokinetics of Higher-Dose Icotinib in Patients With Advanced Non-Small Cell Lung Cancer. *Oncologist*. 2016;21(11):1294-1295d. doi:10.1634/theoncologist.2016-0256
59. Obach RS. Potent Inhibition of Human Liver Aldehyde oxidase by Raloxifene. *Drug Metab Dispos*. 2004;32(1):89-97. doi:10.1124/dmd.32.1.89
60. Fujifilm. Favipiravir, Investigator's Brochure, https://www.principletrial.org/files/trial-documents/faviib_v3-0003.pdf. 2022
